# The effect of climate extremes on disability, depression, and cognitive decline in middle-aged and older Chinese adults under different healthy lifestyle

**DOI:** 10.7189/jogh.15.04266

**Published:** 2025-10-10

**Authors:** Yao Li, Hui Liu, Yijun Xie, Hengjing Wu, Jianke Jiang, Longbing Ren, Jing Wu

**Affiliations:** 1 Clinical Center for Intelligent Rehabilitation Research, Shanghai YangZhi Rehabilitation Hospital (Shanghai Sunshine Rehabilitation Center), School of Medicine, Tongji University, Shanghai, China; 2 School of Nursing, Wannan Medical College, Wuhu, China; 3 School of Public Health, Peking University, Beijing, China; 4 China Center for Health Development Studies, Peking University, Beijing, China

## Abstract

**Background:**

The health risks from climate change are increasing, but the benefits of healthy lifestyles are evident. We aimed to examine the complex relationship between climate change and lifestyles on disability, depression, and cognitive decline in middle-aged and older Chinese adults.

**Methods:**

We used a national cohort from the China Health and Retirement Longitudinal Study from 2011–2018 and constructed 27 core extreme climate indices using daily temperature and precipitation. Participants were categorised into the favourable group (4–5 healthy lifestyle factors), the average group (2–3 factors), and the unfavourable group (0–1 factor). The time-dependent Cox regression and linear mixed-effects models were applied to explore associations and further stratified the analysis by lifestyles.

**Results:**

A total of 5161 participants were included (19.53% were unfavourable, and 57.70% were average lifestyles). During average 51.76 and 52.79 months follow-up, 1097 of 5161 participants suffered disability, and 1181 developed depression. We observed a significant interaction between lifestyle and specific climate extreme indexes in three outcomes, respectively (all *P* < 0.05). Compared to the average and favourable lifestyle groups, the corresponding associations for some extreme heat and temperature indexes were more pronounced for disability (all *P* < 0.05), for some extreme precipitation indexes for depression, and for some extreme heat, cold, and precipitation indexes were much more pronounced (all *P* < 0.05) for cognitive decline in the unfavourable lifestyle group. Similar associations were found in sensitivity analyses.

**Conclusions:**

A healthy lifestyle may attenuate the adverse impacts of frequent climate extremes on disability, depression, and cognitive function among middle-aged and older adults. This study may have important implications for making policies and adaptive strategies to reduce relevant risks to health, especially in low- and middle-income countries.

Global population aging is on the rise. The world's population aged 65 years and over is expected to reach 1.6 billion in 2050, roughly doubling the increase from 2022 [1], with low- and middle-income countries (LMICs) accounting for 80% by then [2]. With an extended lifespan, older adults will be prone to the risk of disability, depression, and cognitive decline, which imposes a considerable burden of diseases on family and nation [3–5]. Expanding public health assessments to include unconventional risk factors, such as environmental exposures, may provide fresh insights into disease prevention.

More frequent climate change has resulted in substantial fluctuations in extreme temperature and precipitation [6]. It has a direct or indirect impact on multiple health outcomes through different physiological mechanisms at the same time, including excess mortality and morbidity associated with non-communicable chronic disease (NCD) [7,8], as well as increased disability burden through both NCD progression and climate-related injuries [9–11]. Notably, accumulating research evidence reveals the particularly severe consequences of extreme climate events on psychological well-being and cognitive function in vulnerable older populations [12–15], with potential mechanisms including thermal stress-induced neuroinflammation and weather-related social isolation. Despite these advances, significant research gaps still remain. First, existing research has predominantly examined high-income settings, while impacts in LMICs – where populations frequently lack the infrastructure and health care capacity to buffer climate impacts – remain markedly understudied. Second, growing evidence suggests that lifestyle modifications may delay cognitive decline [16,17] and potentially reduce risks of disability and depression [18,19]. Preliminary evidences suggest that adopting a healthy lifestyle could reduce the health disparities caused by socioeconomic factors [20–22], and offset adverse environmental effects (for example, plant-based diet reduces the impact of fine particulate matter (PM_2.5_) on cognitive function) in older adults [23]. Although lifestyle factors have been identified as important regulators of various environmental health risks, there is no comprehensive study to test whether and how healthy lifestyles change the relationship between extreme climate and key aging related outcomes (including disability, depression and cognitive decline).

Given the established protective effects of healthy lifestyles against disability, depression, and cognitive decline, coupled with the potential adverse impacts of climate extremes on these health outcomes, we hypothesised that healthy lifestyles may modify the association between climate extremes and these diseases. Therefore, our study aimed to explore their multiplicative interaction between climate extremes and healthy lifestyles and to compare whether climate extremes (extreme temperatures and precipitation) have the same independent effect on these diseases under different lifestyle groups (unfavourable, average, and favourable) among middle-aged and older adults.

## METHODS

### Study design and participants

The China Health and Retirement Longitudinal Study (CHARLS) is a nationwide, population-based cohort study that recruits residents aged 45 and older from 28 provinces using multistage stratified probability-proportionate-to-size sampling. More details about the study can be found elsewhere [24]. Written informed consent was provided by all participants in the baseline and follow-up surveys, and the study was approved by the Biomedical Ethics Committee of Peking University (IRB00001052-11015). We used data from the four waves of the CHARLS run in 2011, 2013, 2015, and 2018. Finally, a total of 5161 participants were included in the analysis. The exclusion criteria are the following:

(i) under 45 years old

(ii) with any missing values in depression, disability, and cognition

(iii) with poor cognitive function (Mini-Mental State Examination (MMSE)<6) or depression or disability at baseline

(iv) die or lost follow-up in the second survey (Figure S1 in the **Online Supplementary Document**).

### Assessments of three outcomes

The 10-item Center for Epidemiological Studies Depression Scale (CESD-10) was used to evaluate depressive symptoms in each wave. Each question was used to measure the frequency of some negative emotion during the last week, where 0 indicated ‘rarely or none’ (<1 day), 1 indicated ‘some days’ (1–2 days), 2 indicated ‘occasionally’ (3–4 days), and 3 indicated ‘most of the times’ (5–7 days). This study reverse-coded questions five and eight; the total score ranges from 0–30, with a score ≥10 considered having depression [25].

Disability was assessed using two scales at each wave. The basic activities of daily living (BADL) scale was measured with six items: dressing, bathing, feeding, moving from bed to chair, using the toilet, and maintaining continence. The instrumental activities of daily living (IADL) scale was measured with five items: doing housework, cooking, shopping, managing money, and taking medication. Each answer was divided into four response options (1 means ‘No, I do not have any difficulty’; 2 means ‘I have difficulty but still can do it’; 3 means ‘Yes, I have difficulty and need help’; 4 means ‘I cannot do it’) with the first response scoring one and the rest scoring zero. The score of ADL is the sum of the calculated BADL and IADL items on a scale of 0–11, and participants who scored more than zero were defined as having a disability [26].

The standardised z-scores of raw MMSE scores at each wave were used to evaluate the rate of cognitive decline, raw MMSE scores were represented by the sum of the Word Recall Test (0–10 points) and the Mental Status Test (0–11 points) (Table S1 in the **Online Supplementary Document**) [25]. First, we calculated the standardised z-score of each domain score by subtracting the baseline mean from the row score and dividing it by the baseline standard deviation (SD). Then, the global z-score of an individual at each wave was calculated by averaging the z-scores of the two domains. A standardised z-score of −1 at any given wave indicated that the score was one SD lower than the mean global z-score at baseline [27].

### Assessment of climate extremes

Prefecture-level cities or other types of prefecture units are included in the publicly released CHARLS data, and the daily meteorological information for all selected cities comes from the China Meteorological Data Service Centre [28]. A more detailed daily meteorological information can be found elsewhere [29]. According to the 27 core extreme climate indices for integrated extreme weather intensity and duration proposed by the World Meteorological Organization [30], we constructed these indices using the daily temperature and precipitation over the period 2008–2018 for each city, which were mainly categorised into four types (extreme heat, extreme cold, other temperature, and extreme precipitation), with the detailed descriptions and classifications of these indices as shown in Table S2 in the **Online Supplementary Document**.

We averaged exposure to climate extremes over the 36 months before enrolment to represent baseline cumulative exposure. For disability and depression, we averaged the estimated exposures over the 36 months prior to the outcome diagnosis, loss to follow-up, death, or the last follow-up to denote recent cumulative exposures. For cognitive decline, we averaged the estimated exposures for 36 months before the loss to follow-up, death, or the last follow-up to indicate recent accumulated exposures. The standardised z-score for recent accumulated exposures was calculated by subtracting the baseline cumulative exposure mean from the recent cumulative exposures and dividing it by the baseline SD. A standardised z-score of one indicates that recent cumulative exposure is one SD above the mean value at baseline for all climate extremes.

### Assessment of healthy lifestyles

Lifestyle information was collected at each wave through a healthy behaviour questionnaire. Healthy lifestyle status was determined by considering five factors: smoking, drinking, physical activity, social contact, and sleep [17,31]. For smoking, participants were classified as: used to smoke, never smoked, or current smokers, and never smoking was deemed a healthy lifestyle factor. Similar classifications were used for drinking. For physical activity, moderate or vigorous activity for at least 150-minute per week was considered a healthy factor (Table S3 in the **Online Supplementary Document**). For social contact (playing mahjong/chess/cards, interacting with friends, going to a sport/social/club, taking part in a community-related organisation, caring for a sick or disabled adult freely, doing voluntary/charity work, attending an educational/training course, using the Internet, stocking investment, and others), participation in at least once a week was considered healthy. Healthy sleep was defined as having 7–9 hours. Ultimately, all participants were divided into unfavourable (0–1 healthy factors), average (2–3), and favourable (4–5) groups based on the previous study [17].

### Covariates

Potential confounders were selected based on relevant references [32,33]. Sociodemographic information obtained in each wave included age, gender, area of residence (urban community or rural village), educational attainment (less than lower secondary, upper secondary & vocational training, or tertiary), marital status (separated/divorced/widowed/never married, or married/partnered), and annual *per capita* household consumption level (based on quintile). Each participant self-reported their disease status (yes or no) for 12 NCDs in each wave, including hypertension, diabetes, dyslipidaemia, stroke, heart disease, chronic lung disease, asthma, liver disease, cancer, digestive disease, kidney disease, and arthritis. Environmental variables collected at the prefecture-level included air pollution (PM_2.5_), green space, and weather conditions (annual cumulative sunshine and air humidity). The ground-level concentrations of PM_2.5_ were calculated from the Atmospheric Composition Analysis Group, providing an annual time series of PM_2.5_ concentrations with approximately 1km^2^ resolution. Green space data was extracted from the China City Statistical Yearbook. Weather conditions were collected from the China Meteorological Data Service Centre.

### Statistical analysis

Continuous variables were presented as means and SDs if distributed normally by the Kolmogorov-Smirnov test; otherwise, medians and interquartile ranges were used. Numbers and proportions were presented for categorical variables. Baseline characteristics were compared between three lifestyle groups using an analysis of variance for normally distributed continuous variables, the Kruskal-Wallis test for non-normally distributed variables, and χ^2^ tests or Fisher exact tests for categorical variables. Missing data was imputed using a multiple imputation chain equation, and five imputed data sets were analysed separately, with their results combined using Rubin's method.

Additionally, time-dependent Cox regression models, which do not require the proportional hazards assumption, were employed to calculate hazard ratios and 95% confidence intervals for lifestyle and climate extremes (a standardised z-score) concerning the progression of depression and disability, considering changes in lifestyle and certain covariates over time. For depression, CESD-10 score at baseline and other covariates at each wave (age, gender, area of residence, educational attainment, marital status, number of chronic diseases, annual *per capita* household consumption level, and other environmental indices) were adjusted in this model. For disability, the model adjusted for the covariates mentioned above except for the CESD-10 score. To test whether the association between climate extremes and disability risk varied by lifestyle, we added a multiplicative interaction term between lifestyle and climate extremes to the model and assessed its significance using a likelihood ratio test. The analyses were repeated for the three groups stratified by lifestyle if a correspondence interaction existed. Linear mixed effects models (LMMs) were used to assess how healthy lifestyle and climate extremes are associated with cognitive decline over time. Before interpreting the results, the standardised z-score of MMSE scores satisfied the normality assumption before implementing the model. The dependent variable was the standardised z-score of MMSE scores. The fixed effects included lifestyle, a standardised z-score of climate extremes, a follow-up year from baseline (time), the multiplicative interaction of lifestyle and time (lifestyle group × time), and the random effects included intercept and time. We also adjusted for the MMSE score at baseline and other covariates at each wave (age, gender, area of residence, educational attainment, marital status, number of chronic diseases, annual *per capita* household consumption level, and other environmental indices) in this model. Additionally, we included healthy lifestyle group  × climate extreme as a multiplicative interaction term in the fixed effect and compared the model with and without the interaction term via likelihood ratio test. The analysis was stratified by three lifestyle groups if an interaction was present. All variables were included in the first step, except for the lifestyle group × time in fixed effects.

Several sensitivity analyses were conducted to ensure the reliability of the above findings. First, the three raw scale scores for depression, disability, and cognitive decline were each included as continuous variables in the LMM dependent variables. Second, quintiles of standardised z-scores for climate extremes were also evaluated as grouping variables in the Cox models and LMMs, respectively. Third, primary analyses were repeated by different age groups (<60 and ≥60), genders (male and female), and areas of residence (urban community and rural village) to test the robustness and potential differences within subgroups. Finally, due to the high number of deaths and drop-out visits among some elderly participants, we used an inverse probability weighting model to assess whether withdrawal from the study impacted the relationship between lifestyle and climate extremes on depression/ disability, and a competing risk model was built to account for bias caused by competing risk from death.

All statistical analysis was carried out using *R* software, version 4.3.1 (*R* Core Team, Vienna, Austria). Some specific packages included mice for imputing, RClimDex for climate extremes indices, survival for time-dependent Cox regression models, lmerTest for linear mixed effects models, ANOVA for likelihood ratio test, IPW for inverse probability weighting, and cmprsk for competing risk models. Statistical significance was determined using a *P*-value less than 0.05 in two-sided testing.

## RESULTS

### The characteristics of study participants

A total of 5161 CHARLS participants were enrolled after applying the study population restriction criteria, and the baseline characteristics according to the three lifestyle groups were summarised in **Table 1**. Among them, 2207 (42.76%) participants were female, with a mean age of 56.64 years (SD = 8.68). More than half (52.35%) of participants were rural residents, 80.84% had less than lower secondary, and most (92.99%) were married/partnered. Additionally, 1008 participants were enrolled in the unfavourable lifestyle group, 2978 in the average group, and 1175 in the favourable group.

**Table 1:** Baseline characteristics of the healthy lifestyle groups

Characteristics	Total (n = 5161)	Unfavourable group (n = 1008)	Average group (n = 2978)	Favourable group (n = 1175)	*P*-value
Age (years)	56.64 ± 8.68	56.94 ± 8.95	56.49 ± 8.84	56.78 ± 8.00	0.299
Gender	2954 (57.24%)				
*Male*	2207 (42.76%)	746 (74.01%)	1599 (53.69%)	609 (51.83%)	<0.001
*Female*		262 (25.99%)	1379 (46.31%)	566 (48.17%)	
Area of residence	2459 (47.65%)				
*Urban community*	2702 (52.35%)	527 (52.28%)	1408 (47.28%)	524 (44.60%)	0.001
*Rural village*		481 (47.72%)	1570 (52.72%)	651 (55.40%)	
Educational attainment	4172 (80.84%)				
*Less than lower secondary*	836 (16.20%)	880 (87.30%)	2384 (80.05%)	908 (77.28%)	<0.001
*Upper secondary & vocational training*	153 (2.96%)	111 (11.01%)	507 (17.02%)	218 (18.55%)	
*Tertiary*		17 (1.69%)	87 (2.92%)	49 (4.17%)	
Marital status	362 (7.01%)				
*Separated/divorced/widowed/never married*	4799 (92.99%)	100 (9.92%)	193 (6.48%)	69 (5.87%)	<0.001
*Married/partnered*		908 (90.08%)	2785 (93.52%)	1106 (94.13%)	
Number of chronic diseases	2071 (40.13%)				
*0*	1553 (30.09%)	360 (35.71%)	1234 (41.44%)	477 (40.60%)	0.021
*1*	1537 (29.78%)	323 (32.04%)	867 (29.11%)	363 (30.89%)	
*≥2*		325 (32.24%)	877 (29.45%)	335 (28.51%)	
Annual *per capita* household consumption level	1033 (20.02%)				
*Quintile 1*	1032 (20.00%)	211 (20.93%)	622 (20.89%)	200 (17.02%)	0.041
*Quintile 2*	1033 (20.02%)	194 (19.25%)	620 (20.82%)	218 (18.55%)	
*Quintile 3*	1031 (19.98%)	205 (20.34%)	578 (19.41%)	250 (21.28%)	
*Quintile 4*	1032 (20.00%)	198 (19.64%)	590 (19.81%)	243 (20.68%)	
*Quintile 5*		200 (19.84%)	568 (19.07%)	264 (22.47%)	
Healthy lifestyle factors	2272(44.02%)				
*PA*	2817 (54.58%)	326 (32.34%)	1254 (42.11%)	693 (58.98%)	<0.001
*Healthy sleep*	2215 (42.92%)	206 (20.44%)	1682 (56.48%)	929 (79.06%)	<0.001
*Never drinking*	2452 (47.51%)	37 (3.67%)	1108 (37.21%)	1070 (91.06%)	<0.001
*Active social contact*	2257 (43.73%)	136 (13.49%)	1437 (48.25%)	879 (74.81%)	<0.001
*Never smoking*	2954 (57.24%)	80 (7.94%)	1132 (38.01%)	1045 (88.94%)	<0.001

PA – physical activity

### Climate extremes and depression/disability in middle-aged and older people

**Figure 1:**
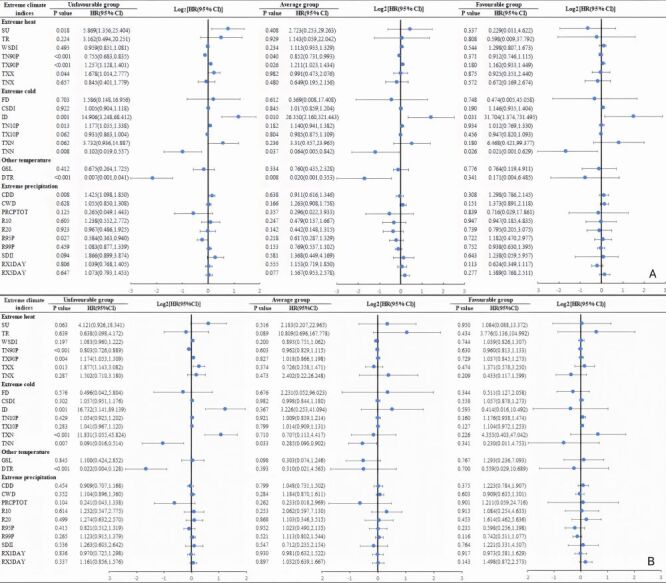
Associations with climate extremes and disability (model 1) and depression (model 2) in a population stratified by healthy lifestyle using Rubin’s method. **Panel A.** Model 1 adjusted covariates of the time-dependent Cox regression model included age, gender, area of residence, educational attainment, marital status, number of chronic diseases, annual *per capita* household consumption level, and other environmental indices. **Panel B.** Model 2 adjusted covariates of the time-dependent Cox regression model included depression score at baseline, age, gender, area of residence, educational attainment, marital status, number of chronic diseases, annual per capita household consumption level, and other environmental indices.

After an average of 51.76 and 52.79 months follow-up, 1097 of 5161 participants (21.26%) suffered disability, and 1181 (22.88%) suffered depression, Kaplan-Meier curves for the overall cumulative incidence and number at risk stratified by lifestyle group were displayed in Figure S2 in the **Online Supplementary Document**, and the analysis showed significant differences in the above cumulative incidence curves (all *P* < 0.05). The Time-dependent Cox regression model suggested that both lifestyles and some climate extremes were associated with disability and depression among all participants over follow-up, respectively (**Table 2**). Furthermore, significant interactions were observed between lifestyle groups and some climate-extreme indexes for both disability and depression (both *P* < 0.05) (Table S4 in the **Online Supplementary Document**). Likelihood ratio tests were performed on models with and without multiplicative interaction terms, and the results for disability and depression indicated significant modification, respectively (χ^2^ = 26.435, *P* = 0.039; χ^2^ = 34.101, *P* = 0.025). We explored the relationship between climate extremes and disability as well as depression by lifestyle subgroups. For disability, there was no association for extreme precipitation in any of the three groups. The corresponding associations for extreme heat (warm nights and warm days) and temperature (mean diurnal temperature range) were more pronounced in the unfavourable lifestyle group (all *P* < 0.05) compared with the average and favourable lifestyle groups (all *P* > 0.05), as well as in the average and unfavourable lifestyle group for extreme cold (icing days and minimum value of daily maximum temperature (TX), all *P* < 0.05) compared with the favourable lifestyle groups (both *P* > 0.05) (**Figure 1**, Panel A), but only the extreme cold indices of minimum value of daily minimum temperature correlated (*P* = 0.033) in the average lifestyle group. For depression, the corresponding associations for extreme cold were similar in all three groups. Notably, the corresponding associations for extreme heat (warm nights and warm days) and temperature (mean diurnal temperature range) were more pronounced in the unfavourable and average lifestyles groups compared with the favourable group (all *P* > 0.05), and for extreme precipitation (consecutive dry days and Avery high rainfall) in unfavourable lifestyles group compared with the average and favourable groups (all *P* > 0.05) (**Figure 1**, Panel B).

**Table 2:** Associations of healthy lifestyle and climate extremes with disability and depression in middle-aged and older adults using Rubin’s method

Characteristics	Disability*		Depression†	
	**HR (95% CI)**	***P*-value**	**HR (95% CI)**	***P*-value**
Lifestyle groups				
*Unfavourable group*	ref.		ref.	
*Average group*	0.756 (0.654, 0.874)	<0.001	0.839 (0.721, 0.975)	0.022
*Favourable group*	0.768 (0.631, 0.935)	0.008	0.652 (0.531, 0.801)	<0.001
Extreme heat				
*SU*	2.426 (0.812, 7.248)	0.113	2.612 (0.845, 8.071)	0.095
*TR*	1.723 (0.44, 6.745)	0.435	1.444 (0.344, 6.068)	0.616
*WSDI*	1.025 (0.938, 1.121)	0.580	1.049 (0.958, 1.149)	0.299
*TN90P*	0.877 (0.815, 0.943)	<0.001	0.812 (0.752, 0.876)	<0.001
*TX90P*	1.12 (1.033, 1.214)	0.006	1.232 (1.134, 1.338)	<0.001
*TXX*	1.408 (0.984, 2.014)	0.061	1.389 (0.956, 2.019)	0.085
*TNX*	0.803 (0.461, 1.397)	0.437	0.761 (0.436, 1.33)	0.338
Extreme cold				
*FD*	0.312 (0.051, 1.925)	0.210	0.841 (0.139, 5.071)	0.850
*CSDI*	1.038 (0.959, 1.123)	0.358	1.036 (0.955, 1.123)	0.395
*ID*	7.017 (1.964, 25.067)	0.003	21.697 (6.684, 70.436)	<0.001
*TN10P*	1.074 (0.977, 1.18)	0.141	1.142 (1.036, 1.258)	0.007
*TX10P*	1.045 (0.99, 1.103)	0.110	0.94 (0.888, 1.995)	0.132
*TXN*	4.07 (1.565, 10.587)	0.004	4.014 (1.458, 11.055)	0.007
*TNN*	0.238 (0.068, 0.829)	0.024	0.073 (0.02, 0.261)	<0.001
Other temperature				
*GSL*	0.788 (0.388, 1.601)	0.511	0.494 (0.242, 1.009)	0.053
*DTR*	0.084 (0.024, 0.301)	<0.001	0.015 (0.004, 0.059)	<0.001
Extreme precipitation				
*CDD*	0.975 (0.811, 1.174)	0.791	1.246 (1.023, 1.517)	0.029
*CWD*	1.071 (0.919, 1.247)	0.380	1.172 (0.996, 1.379)	0.056
*PRCPTOT*	0.319 (0.09, 1.126)	0.076	0.284 (0.079, 1.02)	0.054
*R10*	1.324 (0.726, 2.413)	0.360	0.943 (0.511, 1.74)	0.851
*R20*	1.309 (0.77, 2.225)	0.320	0.870 (0.513, 1.477)	0.607
*R95P*	0.821 (0.575, 1.17)	0.275	0.65 (0.451, 0.937)	0.021
*R99P*	1.026 (0.878, 1.199)	0.745	0.982 (0.834, 1.157)	0.832
SD*II*	1.090 (0.634, 1.872)	0.756	1.241 (0.720, 2.139)	0.437
*RX1DAY*	0.949 (0.764, 1.179)	0.638	0.967 (0.769, 1.216)	0.776
*RX5DAY*	1.196 (0.953, 1.501)	0.123	1.255 (0.995, 1.583)	0.055

CDD – Consecutive dry days, CI – confidence interval, CSDI – Cold spell duration index, CWD – Consecutive wet days, DTR – Mean diurnal temperature range, FD – Frost days, GSL – Growing season length, HR – hazard ratio, ID – icing days, PRCPTOT – Total daily precipitation, R10 – Number of heavy precipitation days, R20 – Number of extremely heavy precipitation days, R95P – Avery high rainfall, R99P – Extremely high rainfall, ref. –reference, RX1DAY – Max 1-d precipitation amount, RX5DAY – Max 5-d precipitation amount, SDII – Simple precipitation intensity index, SU – Summer days, TN10P – Cold nights, TN90P – Warm nights, TNN – Min TN, TNX – Max TN, TR – Tropical nights, TX10P – Cold days, TX90P – Warm days, TXN – Min TX, TXX – Max TX, WSDI – Warm spell duration index

*Adjusted covariates of the time-dependent Cox regression model included age, gender, area of residence, educational attainment, marital status, number of chronic diseases, annual per capita household consumption level, and other environmental indices.

†Adjusted covariates of the time-dependent Cox regression model included depression score at baseline, age, gender, area of residence, educational attainment, marital status, number of chronic diseases, annual per capita household consumption level, and other environmental indices.

Sensitivity analyses were conducted using ADL and depression scores as continuous variables, and the results were consistent with those of the primary analyses (Figure S3 and Table S5–8 in the **Online Supplementary Document**). Then, the correlations were also assessed by quintiles of climate extremes, and the associations are almost similar to those of the primary analysis with continuous variables of climate extremes (Tables S9–10 in the **Online Supplementary Document**). In addition, the results were further stratified by age group, gender, and area of residence (Table S11–16 in the **Online Supplementary Document**). The results for all subgroups were mainly similar to the results of the primary analyses, but for disability, no association was found for extreme cold in the average lifestyle group for the female, older, urban, and rural subgroups; for depression, no association was found for extreme cold in the favourable lifestyle group for the female, middle, old and urban subgroups. Finally, the results were also mostly identical when considering the competing risk of death or participants who withdrew from the study (Table S17–18 in the **Online Supplementary Document**).

### Climate extremes and cognitive decline in middle-aged and older people

**Figure 2:**
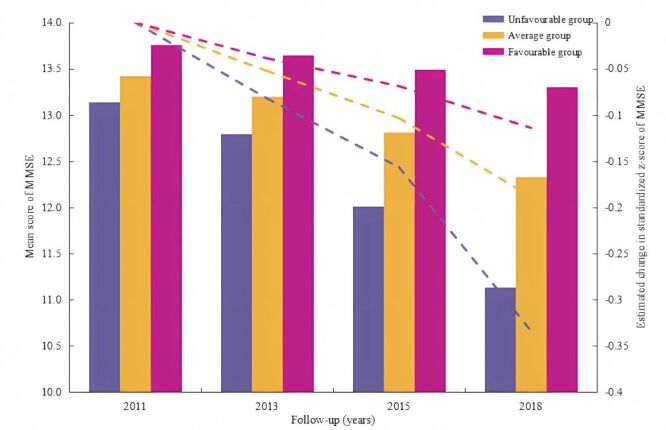
Longitudinal change in mean scores among lifestyle groups over seven years.

The mean raw MMSE scores and standardised z-scores exhibited a continuous decline over the 7-year period (**Figure 2**); the favourable lifestyle group observed the highest raw MMSE scores and standardised z-scores. We used LMMs to examine the influence of healthy lifestyles and climate extremes on the annual cognitive function change rate during the follow-up. As shown in Table S19 in the **Online Supplementary Document**, lifestyle and some climate extremes indices were associated with the standardised z-scores. Compared with the unfavourable group, participants had decelerated cognitive decline (a standardised z-score) of 0.001 and 0.002 SD/y. There was also a significant interaction between lifestyle group and climate extremes for cognitive decline (*P* < 0.05) (Table S20 in the **Online Supplementary Document**). A likelihood ratio test comparing models with and without the multiplicative interaction term indicated significant modification (χ^2^ = 30.369, *P* = 0.031). Additionally, we examined the relationship between climate extremes and cognitive decline in three lifestyle groups, respectively. Corresponding associations for extreme heat (summer days and maximum value of TX), cold (minimum value of TX), and precipitation were much more pronounced for participants with unfavourable lifestyles (all *P* < 0.05) compared to those with average and favourable lifestyles (all *P* > 0.05). However, there was no association for other temperatures in the three groups (**Table 3**).

**Table 3:** Associations with climate extremes and cognitive decline in a population stratified by healthy lifestyle using Rubin’s method

Characteristics	Unfavourable group		Average group		Favourable group	
	Estimate*	***P*-value**	Estimate*	***P*-value**	Estimate*	***P*-value**
Extreme heat						
*SU*	−0.291	0.025	−0.289	0.252	−0.270	0.240
*TR*	0.211	0.203	0.160	0.587	0.261	0.401
*WSDI*	0.004	0.690	−0.007	0.698	−0.035	0.061
*TN90P*	0.008	0.362	0.022	0.176	0.006	0.698
*TX90P*	0.010	0.303	−0.009	0.646	0.007	0.698
*TXX*	−0.108	0.017	−0.054	0.506	−0.066	0.413
*TNX*	−0.111	0.097	−0.062	0.623	−0.008	0.943
Extreme cold						
*FD*	−0.034	0.870	−0.406	0.310	−0.252	0.498
*CSDI*	−0.001	0.925	0.008	0.650	−0.011	0.498
*ID*	−0.204	0.143	−0.152	0.557	−0.077	0.772
*TN10P*	0.010	0.391	−0.025	0.235	−0.007	0.751
*TX10P*	−0.012	0.058	0.007	0.552	0.003	0.768
*TXN*	−0.262	0.028	−0.028	0.897	−0.047	0.821
*TNN*	0.223	0.143	0.065	0.807	0.351	0.189
Other temperature						
*GSL*	0.085	0.304	0.103	0.514	0.073	0.614
*DTR*	0.004	0.979	0.534	0.080	0.344	0.243
Extreme precipitation						
*CDD*	0.005	0.817	0.033	0.417	0.040	0.347
*CWD*	−0.006	0.758	0.014	0.686	0.013	0.700
*PRCPTOT*	0.325	0.029	0.109	0.694	−0.127	0.634
*R10*	−0.185	0.012	−0.205	0.125	−0.149	0.273
*R20*	−0.119	0.051	0.005	0.967	0.098	0.363
*R95P*	−0.058	0.176	−0.034	0.685	0.030	0.690
*R99P*	−0.011	0.542	−0.006	0.877	0.003	0.919
SD*II*	−0.036	0.579	0.000	0.997	0.003	0.983
*RX1DAY*	0.000	0.988	0.015	0.747	0.028	0.559
*RX5DAY*	0.035	0.189	0.004	0.932	0.002	0.966

CDD – Consecutive dry days, CSDI – Cold spell duration index, CWD – Consecutive wet days, DTR – Mean diurnal temperature range, FD – Frost days, GSL – Growing season length, ID – icing days, PRCPTOT – Total daily precipitation, R10 – Number of heavy precipitation days, R20 – Number of extremely heavy precipitation days, R95P – Avery high rainfall, R99P – Extremely high rainfall, RX1DAY – Max 1-d precipitation amount, RX5DAY – Max 5-d precipitation amount, SDII – Simple precipitation intensity index, SU – Summer days, TN10P – Cold nights, TN90P – Warm nights, TNN – Min TN, TNX – Max TN, TR – Tropical nights, TX10P – Cold days, TX90P – Warm days, TXN – Min TX, TXX – Max TX, WSDI – Warm spell duration index

*Adjusted covariates of linear mixed effect model included Mini-Mental State Examination score at baseline, age, gender, area of residence, educational attainment, marital status, number of chronic diseases, annual per capita household consumption level, and other environmental indices.

The relationship between climate extremes and raw MMSE scores for three lifestyle groups was identical to the primary analysis (Table S20–21 in the **Online Supplementary Document**). Corresponding associations for the quintiles of climate extremes were similar to the primary analysis results (Table S22 in the **Online Supplementary Document**). The difference was that extreme cold was not associated with cognitive decline in the three lifestyle groups. We also conducted the primary analysis stratified by age groups, gender, and area of residence (Table S23–25 in the **Online Supplementary Document**). All subgroups' results were the same as the primary analysis. However, the association of extreme cold was not identified in the unfavourable lifestyle group for the female, older, and rural subgroups.

## DISCUSSION

This study elucidates the complex effects of climate extremes on disability, depression, and cognitive decline in different lifestyle groups of Chinese adults aged 45 years and older, respectively. Our study extends beyond the traditional view of lifestyle as a risk factor and emphasises the higher risk of unfavourable lifestyle groups during more frequent climate extremes. Compared to the average and favourable lifestyle groups, the corresponding associations for some extreme heat and temperature indexes were more pronounced for disability (all *P* < 0.05), for some extreme precipitation indexes for depression, and for some extreme heat, cold, and precipitation indexes were much more pronounced (all *P* < 0.05) for cognitive decline in the unfavourable lifestyle group.

Regarding the correlation of temperature with depression and cognition, previous studies have reported J- or U-shaped associations, indicating the possible increased risk of depression and a substantial cognitive decline under extremely hot or cold conditions [14,15,34,35]. Our study has illustrated the impact of climate extremes on health outcomes and strengthened the evidence by considering more indicators. Extreme temperatures can affect the brain's neurotransmitter environment, which may influence mood and cognitive function. Both heat and cold stress may lead to changes in autonomic function, and prolonged imbalance in this system could trigger a chronic low-grade inflammatory response, which is a potential cause of depression [36,37]. Physiological changes caused by heat in the brain include increased focus on specific areas and decreased activity in others, which may lead to cognitive decline in older adults in high-temperature [38]. On the other hand, excessive cold exposure leads to vasoconstriction, which reduces blood circulation capacity and oxygen supply in the brain; this can decrease mitochondrial DNA copy number, mediating the relationship between temperature and cognitive function [39]. While these physiological pathways are biologically plausible, our observational design cannot establish causality, and these mechanisms require verification in experimental studies. Heat action plans require public health or other departments to provide public cooling shelters, health screenings, and water supplies. Indeed, people use either central heating provided by local systems or domestic stoves burning subsidised bulk coal for heating in colder northern China. Promoting more efficient and cleaner heating strategies is essential, especially in constructing future heating systems in low-temperature cities.

Furthermore, precipitation has been found to impact cognition and mental health in older adults by causing them to reduce outdoor interactions and communication, decrease physical activity, and increase the sense of isolation [13]. The correlation between extreme temperatures and disability found in our study may be because the body cools itself by sweating and evaporation at high temperatures, leading to a hypercoagulable blood state and promoting thrombosis. The cold temperature may activate the sympathetic nervous system and promote vasoconstriction, increasing blood pressure and viscosity [9,10]. Heat or cold extremes may contribute to the development of ischemic adverse events, the adverse prognosis of which ultimately leads to disability. However, the biological mechanism of how extreme precipitation affects disability, depressive symptoms, and cognitive function needs further investigation. In addition, climate and public health sectors should collaborate to establish meteorology-informed early warning systems for health. The 2024 report of the Lancet Countdown on health and climate action highlights the escalating health costs of climate change. Paradoxically, LMICs that have historically contributed the least to climate change are bearing the brunt of its health impacts, as they often constrained by scarce financial resources and limited technical and human capacity. Beyond advancing health-centred energy transitions, reducing greenhouse gas emissions, and strengthening national climate monitoring systems, the report emphasises the urgent need to pursue additional health-promoting solutions, which includes addressing structural inequalities [40].

Lifestyle factors are potentially modifiable, and lifestyle-based interventions may effectively reduce the risk of three outcomes in middle-aged and older individuals, similar to the previous studies [16–19]. The annual deceleration rate of cognitive decline in this study was 0.001–0.002 SD for those over 45 years old, although the effect size is modest, even small changes can have meaningful cumulative public health implications over time when scaled across a large aging population. Nevertheless, future research should consider the minimal clinically important difference. Notably, there was a significant interaction between lifestyle and certain climate extremes for three outcomes, with healthier lifestyles could offset some health risks associated with certain climate extremes. To reduce the climate-related health risks described above at limited cost and with limited technology, it is essential for public policies in LMICs to support and create environments that encourage healthy habits, such as implementing smoking bans in public places and providing spaces for exercise and recreation. The ‘Healthy China 2030’ Planning has set requirements to improve national health literacy and support the promotion of traditional sports such as Tai Chi and health qigong. By 2030, the goals include establishing a three-tier (county-township-village) public sports facility network and reducing the smoking rate among people aged 15 and above to 20%. Future research could explore the practical implications of how this policy intervention moderates the interaction between extreme climates and health outcomes. Similarly, primary subgroup analyses demonstrated the diluting effect of healthier lifestyles on certain climate extremes. However, climate extreme vulnerability differs among females, rural residents, and older adults. One possible explanation for this difference is that female's physiological and psychological conditions are different from those of males, but the exact reason needs further investigation. In addition, rural residents may have limited heating in winter, fewer double/triple panes of glass in their dwellings, and lower air conditioning usage in the summer than their urban counterparts, whose living environments and construction facilities households are susceptible to disturbances in external climates [41,42]. Other research has also suggested that older adults may be the most vulnerable to extreme temperatures [43]. As the world's largest developing nation with a rapidly aging population, one third of China’s population living in rural areas, our study can inform policymakers about the health benefits of improving health lifestyles in mitigating climate-related risks. This evidence can ensure the well-being and resilience of different subgroups of the middle-aged and older population in the face of extreme climate change.

The main strength of our results lies in a nationwide representative longitudinal cohort of middle-aged and older adults in China. The large sample size can allow for detailed analyses with sufficient statistical power. Then, the research minimised the risk of ecological fallacies by using high-resolution satellite data and a time-varying method to capture the dynamic nature of climate extremes at the individual level of each participant. Finally, some sensitivity analyses were conducted to demonstrate the robustness of the findings.

Our study also has some limitations. First, the lifestyle assessment relied on self-reporting and was subject to measurement error despite strict collection criteria. Second, although longitudinal designs using time-dependent cox regressions and LMMs of all wave variables prior to disease onset are helpful in determining temporality, the possibility of reverse causality between certain lifestyles (such as sleep and social contact) and outcomes cannot be completely ruled out in observational studies, even if disease populations are excluded at baseline. Third, excluding some subjects due to deaths, lost visits, and missing data may have introduced selection bias. Nevertheless, the corresponding sensitivity analyses produced consistent results. Fourth, it was impossible to eliminate all residual confounding factors due to limited covariates (such as lack of genotype information), but the findings were adjusted for individual characteristics, significant comorbidities, and other environmental variables. Fifth, while the climate indicators were derived from authoritative China Meteorological Administration data using 36-month cumulative exposure to reduce short-term fluctuations, city-level meteorological data may still lead to exposure misclassification by failing to account for localised microclimates or individual behavioural adaptations (such as air conditioning or heating use). Finally, our study encompassed middle-aged and older individuals in a broad range of locations within China. Although the results need to be more generalisable to other populations, this allows for national and international comparisons. Future research should validate and extend these findings in various regions and contexts.

## CONCLUSIONS

Our findings indicated that adherence to favourable lifestyles may benefit middle-aged and older adults with disability, depression, and cognitive decline induced by long-term cumulative exposure to certain climate extremes. To promote healthy aging and lessen the burden on caregivers, policymakers should implement policies and regulations to improve the physical environment conducive to healthy lifestyles and develop community-based response strategies for climate extremes to reduce health risks, especially in LMICs.

## Additional material


Online Supplementary Document

## References

[1] Nations tPDotDoEaSAotU. World Population Prospects 2022. 2022. Available: https://population.un.org/wpp/. Accessed: 10 July 2024.

[2] World Health Organization. World report on ageing and health. Geneva, Switzerland: World Health Organization; 2015. Available: https://www.who.int/publications/i/item/9789241565042. Accessed: 10 July 2024.

[3] Rajan KB Weuve J Barnes LL McAninch EA Wilson RS Evans DA Population estimate of people with clinical Alzheimer’s disease and mild cognitive impairment in the United States (2020-2060). Alzheimers Dement. 2021;17:1966–75. 10.1002/alz.12362 34043283 PMC9013315

[4] Collaborators GBDMD Global, regional, and national burden of 12 mental disorders in 204 countries and territories, 1990-2019: a systematic analysis for the Global Burden of Disease Study 2019. Lancet Psychiatry. 2022;9:137–50. 10.1016/S2215-0366(21)00395-3 35026139 PMC8776563

[5] Cieza A Causey K Kamenov K Hanson SW Chatterji S Vos T Global estimates of the need for rehabilitation based on the Global Burden of Disease study 2019: a systematic analysis for the Global Burden of Disease Study 2019. Lancet. 2021;396:2006–17. 10.1016/S0140-6736(20)32340-0 33275908 PMC7811204

[6] Burrows K Fussell E A life course epidemiology approach to climate extremes and human health. Lancet Planet Health. 2022;6:e549–50. 10.1016/S2542-5196(22)00146-2 35809583 PMC9773632

[7] Arisco NJ Sewe MO Barnighausen T Sie A Zabre P Bunker A The effect of extreme temperature and precipitation on cause-specific deaths in rural Burkina Faso: a longitudinal study. Lancet Planet Health. 2023;7:e478–89. 10.1016/S2542-5196(23)00027-X 37286245

[8] Alahmad B Khraishah H Roye D Vicedo-Cabrera AM Guo Y Papatheodorou SI Associations Between Extreme Temperatures and Cardiovascular Cause-Specific Mortality: Results From 27 Countries. Circulation. 2023;147:35–46. 10.1161/CIRCULATIONAHA.122.061832 36503273 PMC9794133

[9] Cramer MN Gagnon D Laitano O Crandall CG Human temperature regulation under heat stress in health, disease, and injury. Physiol Rev. 2022;102:1907–89. 10.1152/physrev.00047.2021 35679471 PMC9394784

[10] Kranc H Novack V Shtein A Sherman R Novack L Extreme temperature and out-of-hospital-cardiac-arrest. Nationwide study in a hot climate country. Environ Health. 2021;20:38. 10.1186/s12940-021-00722-1 33820550 PMC8022396

[11] Sayem Ahmed MZH Montira J Pongsiri, Mohammad Wahid Ahmed, Sylvia Szabo. Effect of extreme weather events on injury, disability, and death in Bangladesh. Clim Dev. 2020;13:306–17. 10.1080/17565529.2020.1772705

[12] Wahid SS Raza WA Mahmud I Kohrt BA Climate-related shocks and other stressors associated with depression and anxiety in Bangladesh: a nationally representative panel study. Lancet Planet Health. 2023;7:e137–46. 10.1016/S2542-5196(22)00315-1 36754469

[13] Hou K Xu X Ambient temperatures associated with reduced cognitive function in older adults in China. Sci Rep. 2023;13:17414. 10.1038/s41598-023-44776-2 37833389 PMC10575877

[14] Zhao Q Wigmann C Areal AT Altug H Schikowski T Effect of non-optimum ambient temperature on cognitive function of elderly women in Germany. Environ Pollut. 2021;285:117474. 10.1016/j.envpol.2021.117474 34087635

[15] Jin J Xu Z Cao R Wang Y Zeng Q Pan X Long-Term Apparent Temperature, Extreme Temperature Exposure, and Depressive Symptoms: A Longitudinal Study in China. Int J Environ Res Public Health. 2023;20:3229. 10.3390/ijerph20043229 36833923 PMC9962105

[16] Jia J Zhao T Liu Z Liang Y Li F Li Y Association between healthy lifestyle and memory decline in older adults: 10 year, population based, prospective cohort study. BMJ. 2023;380:e072691. 10.1136/bmj-2022-072691 36696990 PMC9872850

[17] Dhana K Evans DA Rajan KB Bennett DA Morris MC Healthy lifestyle and the risk of Alzheimer dementia: Findings from 2 longitudinal studies. Neurology. 2020;95:e374–83. 10.1212/WNL.0000000000009816 32554763 PMC7455318

[18] Xu C Cao Z Huang X Wang X Associations of healthy lifestyle with depression and post-depression dementia: A prospective cohort study. J Affect Disord. 2023;327:87–92. 10.1016/j.jad.2023.01.111 36736794

[19] Abe T Seino S Nofuji Y Yokoyama Y Amano H Yamashita M Modifiable healthy behaviours and incident disability in older adults: Analysis of combined data from two cohort studies in Japan. Exp Gerontol. 2023;173:112094. 10.1016/j.exger.2023.112094 36681130

[20] Foster HME Ho FK Mair FS Jani BD Sattar N Katikireddi SV The association between a lifestyle score, socioeconomic status, and COVID-19 outcomes within the UK Biobank cohort. BMC Infect Dis. 2022;22:273. 10.1186/s12879-022-07132-9 35351028 PMC8964028

[21] Li L Ouyang F He J Qiu D Luo D Xiao S Associations of Socioeconomic Status and Healthy Lifestyle With Incidence of Dyslipidemia: A Prospective Chinese Governmental Employee Cohort Study. Front Public Health. 2022;10:878126. 10.3389/fpubh.2022.878126 35757615 PMC9218108

[22] Li Y Tang Y Lu J Wu H Ren L The dilution effect of healthy lifestyles on the risk of cognitive function attributed to socioeconomic status among Chinese older adults: A national wide prospective cohort study. J Glob Health. 2024;14:04010. 10.7189/jogh-14-04010 38304974 PMC10835516

[23] Zhu A Chen H Shen J Wang X Li Z Zhao A Interaction between plant-based dietary pattern and air pollution on cognitive function: a prospective cohort analysis of Chinese older adults. Lancet Reg Health West Pac. 2022;20:100372. 10.1016/j.lanwpc.2021.100372 35028630 PMC8741490

[24] Zhao Y Hu Y Smith JP Strauss J Yang G Cohort profile: the China Health and Retirement Longitudinal Study (CHARLS). Int J Epidemiol. 2014;43:61–8. 10.1093/ije/dys203 23243115 PMC3937970

[25] Rong H Lai X Jing R Wang X Fang H Mahmoudi E Association of Sensory Impairments With Cognitive Decline and Depression Among Older Adults in China. JAMA Netw Open. 2020;3:e2014186. 10.1001/jamanetworkopen.2020.14186 32990739 PMC7525357

[26] Gong J Wang G Wang Y Chen X Chen Y Meng Q Nowcasting and forecasting the care needs of the older population in China: analysis of data from the China Health and Retirement Longitudinal Study (CHARLS). Lancet Public Health. 2022;7:e1005–13. 10.1016/S2468-2667(22)00203-1 36423656 PMC9741660

[27] Li C Zhu Y Ma Y Hua R Zhong B Xie W Association of Cumulative Blood Pressure With Cognitive Decline, Dementia, and Mortality. J Am Coll Cardiol. 2022;79:1321–35. 10.1016/j.jacc.2022.01.045 35393012

[28] National Meteorological Information Center. China Meteorological Data Service Centre. 2023. Available: http://data.cma.cn/. Accessed: 5 May 2023.

[29] Jin J Wang Y Xu Z Cao R Zhang H Zeng Q Long-Term Temperature Variability and Risk of Dyslipidemia Among Middle-Aged and Elderly Adults: A Prospective Cohort Study - China, 2011-2018. China CDC Wkly. 2022;4:561–4. 10.46234/ccdcw2022.122 35919457 PMC9339356

[30] Peterson TC, Folland C, Gruza G, Hogg W, Mokssit A, Plummer N. Report on the Activities of the Working Group on Climate Change Detection and Related Rapporteurs 1998-2001. Geneva, Switzerland: World Meteorological Organization; 2001.

[31] Sabia S Dugravot A Leger D Ben Hassen C Kivimaki M Singh-Manoux A Association of sleep duration at age 50, 60, and 70 years with risk of multimorbidity in the UK: 25-year follow-up of the Whitehall II cohort study. PLoS Med. 2022;19:e1004109. 10.1371/journal.pmed.1004109 36256607 PMC9578599

[32] Qiu X Shi L Kubzansky LD Wei Y Castro E Li H Association of Long-term Exposure to Air Pollution With Late-Life Depression in Older Adults in the US. JAMA Netw Open. 2023;6:e2253668. 10.1001/jamanetworkopen.2022.53668 36763364 PMC9918878

[33] Zhang L Luo Y Zhang Y Pan X Zhao D Wang Q Green Space, Air Pollution, Weather, and Cognitive Function in Middle and Old Age in China. Front Public Health. 2022;10:871104. 10.3389/fpubh.2022.871104 35586008 PMC9108722

[34] Dai L Kloog I Coull BA Sparrow D Spiro A III Vokonas PS Cognitive function and short-term exposure to residential air temperature: A repeated measures study based on spatiotemporal estimates of temperature. Environ Res. 2016;150:446–51. 10.1016/j.envres.2016.06.036 27391696 PMC5003630

[35] Liu J Varghese BM Hansen A Xiang J Zhang Y Dear K Is there an association between hot weather and poor mental health outcomes? A systematic review and meta-analysis. Environ Int. 2021;153:106533. 10.1016/j.envint.2021.106533 33799230

[36] Halaris A Inflammation and depression but where does the inflammation come from? Curr Opin Psychiatry. 2019;32:422–8. 10.1097/YCO.0000000000000531 31192815

[37] Greaney JL Kenney WL Alexander LM Sympathetic regulation during thermal stress in human aging and disease. Auton Neurosci. 2016;196:81–90. 10.1016/j.autneu.2015.11.002 26627337 PMC4846507

[38] Walter EJ Carraretto M The neurological and cognitive consequences of hyperthermia. Crit Care. 2016;20:199. 10.1186/s13054-016-1376-4 27411704 PMC4944502

[39] Dolcini J Kioumourtzoglou MA Cayir A Sanchez-Guerra M Brennan KJ Dereix AE Age and mitochondrial DNA copy number influence the association between outdoor temperature and cognitive function: Insights from the VA Normative Aging Study. Environ Epidemiol. 2020;4:e0108. 10.1097/EE9.0000000000000108 32832843 PMC7423527

[40] Romanello M Walawender M Hsu SC Moskeland A Palmeiro-Silva Y Scamman D The 2024 report of the Lancet Countdown on health and climate change: facing record-breaking threats from delayed action. Lancet. 2024;404:1847–96. 10.1016/S0140-6736(24)01822-1 39488222 PMC7616816

[41] Khan AM Finlay JM Clarke P Sol K Melendez R Judd S Association between temperature exposure and cognition: a cross-sectional analysis of 20,687 aging adults in the United States. BMC Public Health. 2021;21:1484. 10.1186/s12889-021-11533-x 34325692 PMC8323228

[42] Wang YJ Chen XP Chen WJ Zhang ZL Zhou YP Jia Z Ethnicity and health inequalities: an empirical study based on the 2010 China survey of social change (CSSC) in Western China. BMC Public Health. 2020;20:637. 10.1186/s12889-020-08579-8 32380963 PMC7204236

[43] Xi D Liu L Zhang M Huang C Burkart KG Ebi K Risk factors associated with heatwave mortality in Chinese adults over 65 years. Nat Med. 2024;30:1489–98. 10.1038/s41591-024-02880-4 38528168

