# Supplementary material for: The effect of climate extremes on disability, depression, and cognitive decline in middle-aged and older Chinese adults under different healthy lifestyle
Source: J Glob Health. 2025 Oct 10;15:04266. doi: 10.7189/jogh.15.04266 (PMC12512054; doi:10.7189/jogh.15.04266)
Supplement: Online Supplementary Document [file jogh-15-04266-s001.pdf]

Supplement to: Li Y, Liu H, Xie Y, Wu H, Jiang J, Ren L, Wu J. The effect of climate extremes on disability, depression, and cognitive decline in middle-aged and older Chinese adults under different healthy lifestyle. J Glob Health. 2025;15:04266.

Supplementary table 1. The Chinese version of the mini-mental state examination adopted in the CHARLS. 1

Supplementary table 2. Definition of extreme climate indices. 2

Supplementary table 3. The amount of time spending on different types of physical activities in a usual week in the CHARLS. 4

Supplementary table 4. Interaction between healthy lifestyle and climate extremes with disability and depression in middle-aged and older adults using Rubin's method. 5

Supplementary table 5. Associations of healthy lifestyle and climate extremes with ADL scale and depression scale scores in middle-aged and older adults using Rubin's method. 7

Supplementary table 6. Interaction between healthy lifestyle and climate extremes with ADL scale and depression scale scores in middle-aged and older adults using Rubin's method. 9

Supplementary table 7. Associations with climate extremes and ADL scale scores in a population stratified by healthy lifestyle using Rubin's method. 11

Supplementary table 8. Associations with climate extremes and depression scale scores in a population stratified by healthy lifestyle using Rubin's method. 12

Supplementary table 9. Associations with quintiles of climate extremes and disability in a population stratified by healthy lifestyle using Rubin's method. 13

Supplementary table 10. Associations with quintiles of climate extremes and depression in a population stratified by healthy lifestyle using Rubin's method. 17

Supplementary table 11. Associations with climate extremes and disability in a population stratified by healthy lifestyle using Rubin's method: subgroup analyses by age 21

Supplementary table 12. Associations with climate extremes and disability in a population stratified by healthy lifestyle using Rubin's method: subgroup analyses by gender 23

Supplementary table 13. Associations with climate extremes and disability in a population stratified by healthy lifestyle using Rubin's method: subgroup analyses by area of residence 25

Supplementary table 14. Associations with climate extremes and depression in a population stratified by healthy lifestyle using Rubin's method: subgroup analyses by age 27

Supplementary table 15. Associations with climate extremes and depression in a population stratified by healthy lifestyle using Rubin's method: subgroup analyses by gender 29

Supplementary table 16. Associations with climate extremes and depression in a population stratified by healthy lifestyle using Rubin's method: subgroup analyses by area of residence 31

Supplementary table 17. Associations with climate extremes and disability in a population stratified by healthy lifestyle using Rubin's method by adjusting with the death as the competing risk and using the inverse probability weighting method. 33

Supplementary table 18. Associations with climate extremes and depression in a population stratified by healthy lifestyle using Rubin's method by adjusting with the death as the competing risk and using the inverse probability weighting method. 35

Supplementary table 19. Associations of healthy lifestyle and climate extremes with cognitive decline in middle-aged and older adults using Rubin's method. 37

Supplementary table 20. Interaction between healthy lifestyle and climate extremes with cognitive decline in middle-aged and older adults using Rubin's method.. 39

Supplementary table 21. Associations with climate extremes and MMSE scores in a population stratified by healthy lifestyle using Rubin's method. 41
Supplementary table 22. Associations with quintiles of climate extremes and cognitive decline in a population stratified by healthy lifestyle using Rubin's method. 42
Supplementary table 23. Associations with climate extremes and cognitive decline in a population stratified by healthy lifestyle using Rubin's method: subgroup analyses by age 46
Supplementary table 24. Associations with climate extremes and cognitive decline in a population stratified by healthy lifestyle using Rubin's method: subgroup analyses by gender 48
Supplementary table 25. Associations with climate extremes and cognitive decline in a population stratified by healthy lifestyle using Rubin's method: subgroup analyses by area of residence 50
Supplementary figure 1. Flow chart of the included CHARLS individuals. 52
Supplementary figure 2. Kaplan-Meier curves for the overall cumulative incidence of outcome and number at risk. 53
Supplementary figure 3. Longitudinal change in mean scores among different lifestyle groups over 7 years. A: mean score of ADL scale; B: mean score of depression scale. 54
STROBE Statement—Checklist of items that should be included in reports of cohort studies. 54

Supplementary table 1. The Chinese version of the mini-mental state examination adopted in the CHARLS.

Domain	Question	Score (Total = 21)
Word Recall Test (the mean of the immediate and delayed recalls)	Immediate recall, participants were asked to immediately repeat 10 Chinese nouns that had just been read to them in any order.	10
	Delayed recall, participants were asked to recall the same word list 4 minutes later	10
Mental Status Test (the sum of these questions)	Participants were asked to complete 7 serial subtractions from 100 (up to 5 times, 1 point for each calculation)	5
	Participants were answered what day it was (month, day, year) .	3
	Participants were answered what week it was (Monday, Tuesday, Wednesday, Thursday, Friday, Saturday, or Sunday) .	1
	Participants were answered what what season of the year it was (Spring, Summer, Fall, or Winter) .	1
	Participants were asked to draw assign picture.	1

Note: The total scores ranged between 0 and 21, with a higher score implying better cognitive performance.

Supplementary table 2. Definition of extreme climate indices.

Code	Index	Description	Unit
Extreme heat indices			
SU	Summer days	Annual count of days when TX > 25°C	Days
TR	Tropical nights	Annual count of days when TN > 20°C	Days
WSDI	Warm spell duration index	Annual count of days with at least 6 consecutive days when TX > 90th percentile	Days
TN90P	Warm nights	Number of days when TN < 90th percentile	Days
TX90P	Warm days	Number of days when TX < 90th percentile	Days
TXX	Max TX	Annual maximum value of TX	°C
TNX	Max TN	Annual maximum value of TN	°C
Extreme cold indices			
FD	Frost days	Annual count of days when TN < 0°C	Days
CSDI	Cold spell duration index	Annual count of days with at least 6 consecutive days when TN < 10th percentile	Days
ID	lcing days	Annual count of days when TX < 0°C	Days
TN10P	Cold nights	Number of days when TN < 10th percentile	Days
TX10P	Cold days	Number of days when TX < 10th percentile	Days
TXN	Min TX	Annual minimum value of TX	°C
TNN	Min TN	Annual minimum value of TN	°C
Other temperature indices			
GSL	Growing season length	Time span between dates with 6 consecutive days of average temperature > 5°C after January 1 and 6 consecutive days of average temperature <5°C after July 1	Days
DTR	Mean diurnal temperature range	Difference between annual average TX and TN	°C
Extreme precipitation indices			
CDD	Consecutive dry days	Maximum number of consecutive days with daily rainfall < 1.0 mm	Days
CWD	Consecutive wet days	Maximum number of consecutive days with daily rainfall ≥ 1.0 mm	Days
PRCPTOT	Total daily precipitation	Annual total precipitation of wet days (defined as PRCP ≥ 1.0 mm) in the year	mm
R10	Number of heavy precipitation days	Annual count of days when PRCP ≥ 10 mm	Days
R20	Number of extremely heavy precipitation days	Annual count of days when PRCP ≥ 20 mm	Days
R95P	Avery high rainfall	Annual total PRCP when daily rainfall > 95th percentile	mm
R99P	Extremely high rainfall	Annual total PRCP when daily rainfall > 99th percentile	mm

SDII	Simple precipitation intensity index	Annual total precipitation divided by the number of wet days (defined as PRCP ≥ 1.0 mm) in the year	mm/day
RX1DAY	Max 1-day precipitation amount	Monthly maximum 1-day precipitation	mm
RX5DAY	Max 5-day precipitation amount	Monthly maximum consecutive 5-day precipitation	mm

*TX and TN represent the daily maximum temperature and daily minimum temperature, respectively.
PRCP: precipitation

Supplementary table 3. The amount of time spending on different types of physical activities in a usual week in the CHARLS.

Physical activities	During a usual week, did you do any [….] for at least 10 minutes continuously?	During a usual week, on how many days did you do [….] for at least 10 minutes?	How much time did you usually spend doing [….] on one of those days?
Now think about all the vigorous activities requiring hard/high-intensity physical effort that you do in a usual week. Vigorous activities make you breathe much harder than normal and may include heavy lifting, digging, plowing, aerobics, fast bicycling, and cycling with a heavy load. Think only about those physical activities that you did for at least 10 minutes at a time.	1. Yes;<br>2. No.	① 1 day<br>② 2 days<br>③ 3 days<br>④ 4 days<br>⑤ 5 days<br>⑥ 6 days<br>⑦ 7 days	1) < 30 minutes<br>2) ≥30 minutes and < 2 hours<br>3) ≥ 2 hours and < 4 hours<br>4) ≥4 hours
Now think about activities which take moderate physical effort that you do in a usual week. Moderate physical activities make you breathe somewhat harder than normal and may include carrying light loads, bicycling at a regular pace, or mopping the floor. Again, think about only those physical activities that you did for at least 10 minutes at a time.	1. Yes;<br>2. No.	① 1 day<br>② 2 days<br>③ 3 days<br>④ 4 days<br>⑤ 5 days<br>⑥ 6 days<br>⑦ 7 days	1) < 30 minutes<br>2) ≥30 minutes and < 2 hours<br>3) ≥ 2 hours and < 4 hours<br>4) ≥4 hours
Now think about the time you spend walking in a usual week. This includes at work and at home, walking to travel from place to place, and any other walking that you might do solely for recreation, sport, exercise, or leisure	1. Yes;<br>2. No.	① 1 day<br>② 2 days<br>③ 3 days<br>④ 4 days<br>⑤ 5 days<br>⑥ 6 days<br>⑦ 7 days	1) < 30 minutes<br>2) ≥30 minutes and < 2 hours<br>3) ≥ 2 hours and < 4 hours<br>4) ≥4 hours

Note: Since no specific duration was mentioned in the questionnaire, drawing on the treatment of other scholars, we converted the time range and took the middle value. That is, "≥10 minutes and <30 minutes" was recorded as 20 minutes, "≥30 minutes and <2 hours" as 75 minutes, "≥2 hours and <4 hours" as 180 minutes, and "≥4 hours" as 240 minutes.That is "≥10 min and <30 min" was recorded as 20 min, "≥30 min and <2 h" was recorded as 75 min, "≥2 h and <4 h" was recorded as 180 min, and "≥4 h" was recorded as 240 min.

Supplementary table 4. Interaction between healthy lifestyle and climate extremes with disability and depression in middle-aged and older adults using Rubin's method.

Characteristics	Disability[a]		Depression[b]	
	HR(95%CI)	P value	HR(95%CI)	P value
**Extreme heat**				
Average group*SU	1.825(0.134,24.951)	0.652	2.464(0.187,32.472)	0.493
Favorable group*SU	0.874(0.031,24.877)	0.937	3.100(0.146,65.797)	0.468
Average group*TR	2.414(0.089,65.829)	0.601	0.106(0.005,2.340)	0.155
Favorable group*TR	0.165(0.002,17.225)	0.447	0.123(0.002,7.331)	0.315
Average group*WSDI	0.843(0.684,1.039)	0.109	1.017(0.829,1.249)	0.871
Favorable group*WSDI	1.074(0.809,1.427)	0.620	0.926(0.707,1.214)	0.580
Average group*TN90P	0.782(0.688,0.888)	<0.001	0.855(0.723,1.011)	0.067
Favorable group*TN90P	0.848(0.732,0.983)	0.028	0.624(0.555,0.931)	0.046
Average group*TX90P	1.108(1.009,1.350)	0.010	1.157(0.958,1.396)	0.129
Favorable group*TX90P	1.096(1.004,1.417)	0.023	1.143(1.008,1.439)	0.035
Average group*TXX	0.995(0.439,2.256)	0.991	1.398(0.641,3.052)	0.400
Favorable group*TXX	0.522(0.18,1.513)	0.231	0.85(0.323,2.239)	0.742
Average group*TNX	1.149(0.309,4.264)	0.836	3.645(0.987,13.468)	0.052
Favorable group*TNX	1.207(0.225,6.461)	0.826	2.836(0.564,14.264)	0.206
**Extreme cold**				
Average group*FD	1.311(0.016,109.411)	0.905	0.039(0,3.042)	0.144
Favorable group*FD	15.123(0.058,371.087)	0.339	0.035(0,8.952)	0.236
Average group*CSDI	0.943(0.777,1.145)	0.553	0.87(0.719,1.052)	0.150
Favorable group*CSDI	1.150(0.900,1.470)	0.263	0.987(0.783,1.243)	0.910
Average group*ID	2.088(0.864,5.749)	0.004	0.721(0.036,14.398)	0.830
Favorable group*ID	5.744(4.88,9.782)	0.001	0.085(0.002,3.911)	0.207
Average group*TN10P	1.16(0.939,1.433)	0.167	1.332(1.081,1.640)	0.007
Favorable group*TN10P	1.002(0.751,1.337)	0.987	1.219(1.038,1.582)	0.038
Average group*TX10P	0.927(0.812,1.058)	0.262	0.923(0.816,1.044)	0.203
Favorable group*TX10P	0.868(0.73,1.031)	0.106	1(0.858,1.166)	0.999
Average group*TXN	2.185(1.336,20.225)	0.041	7.579(0.869,66.109)	0.067
Favorable group*TXN	1.246(1.067,23.139)	0.033	5.191(0.344,78.316)	0.234
Average group*TNN	0.642(0.031,13.383)	0.775	0.061(0.003,1.181)	0.064
Favorable group*TNN	0.597(0.112,0.992)	0.045	0.199(0.005,8.545)	0.400
**Other temperature**				
Average group*GSL	3.711(0.679,20.281)	0.130	1.671(0.309,9.034)	0.551
Favorable group*GSL	10.335(0.820,89.010)	0.134	4.765(0.564,40.226)	0.151
Average group*DTR	0.339(0.015,0.783)	0.032	0.142(0.004,4.880)	0.279
Favorable group*DTR	0.574(0.111,0.968)	0.009	0.010(0.001,0.190)	0.002
**Extreme precipitation**				
Average group*CDD	1.166(0.744,1.827)	0.503	0.458(0.297,0.707)	<0.001

Favorable group*CDD	1.249(0.704,2.217)	0.447	0.697(0.527,0.925)	0.038
Average group*CWD	0.693(0.47,1.021)	0.064	0.842(0.581,1.22)	0.364
Favorable group*CWD	0.855(0.516,1.417)	0.543	0.662(0.425,1.033)	0.069
Average group*PRCPTOT	2.708(0.14,52.535)	0.510	0.104(0.006,1.819)	0.121
Favorable group*PRCPTOT	8.506(0.185,390.089)	0.273	0.531(0.014,19.857)	0.732
Average group*R10	2.449(0.571,10.495)	0.228	2.068(0.499,8.568)	0.316
Favorable group*R10	2.392(0.357,16.05)	0.369	2.831(0.467,17.142)	0.257
Average group*R20	1.168(0.346,3.946)	0.802	1.909(0.561,6.493)	0.300
Favorable group*R20	0.615(0.131,2.886)	0.538	0.936(0.211,4.152)	0.930
Average group*R95P	0.477(0.212,1.071)	0.073	0.685(0.435,0.955)	0.019
Favorable group*R95P	0.78(0.283,2.147)	0.630	0.745(0.67,0.997)	0.039
Average group*R99P	1.418(0.947,2.123)	0.090	1.239(0.849,1.808)	0.267
Favorable group*R99P	1.272(0.763,2.122)	0.356	0.868(0.541,1.391)	0.556
Average group*SDII	1.624(0.458,5.764)	0.453	1.636(0.456,5.865)	0.450
Favorable group*SDII	0.709(0.135,3.739)	0.686	0.731(0.151,3.533)	0.697
Average group*RX1DAY	0.858(0.508,1.451)	0.568	0.778(0.472,1.282)	0.324
Favorable group*RX1DAY	0.73(0.368,1.448)	0.367	0.982(0.527,1.832)	0.956
Average group*RX5DAY	0.68(0.393,1.177)	0.168	1.732(0.909,2.975)	0.146
Favorable group*RX5DAY	0.683(0.335,1.392)	0.294	1.474(0.75,2.896)	0.260

[a]Adjusted covariates of the time-dependent Cox regression model included lifestyle groups, climate extremes indexes, age, gender, area of residence, educational attainment, marital status, number of chronic diseases, annual per capita household consumption level, and other environmental indices.

[b]Adjusted covariates of the time-dependent Cox regression model included lifestyle groups, climate extremes indexes, depression score at baseline, age, gender, area of residence, educational attainment, marital status, number of chronic diseases, annual per capita household consumption level, and other environmental indices.

Supplementary table 5. Associations of healthy lifestyle and climate extremes with ADL scale and depression scale scores in middle-aged and older adults using Rubin's method.

Characteristics	ADL scale		Depression scale	
	Estimate	P value	Estimate	P value
Age	0.001	0.155	-0.012	0.001
Gender (Female)	-0.002	0.733	0.191	0.001
Area of residence (Rural Village)	-0.004	0.547	0.122	0.033
Educational attainment				
Less than lower secondary	Reference		Reference	
Upper secondary & vocational training	-0.001	0.912	-0.051	0.475
Tertiary	0.000	0.995	-0.018	0.908
Marital status (Married/partnered)	-0.005	0.630	-0.400	<0.001
Number of chronic diseases				
0	Reference		Reference	
1	0.000	0.938	0.038	0.510
≥2	-0.003	0.680	0.279	<0.001
Annual per capita household consumption level				
Quintile 1	Reference		Reference	
Quintile 2	-0.001	0.838	0.025	0.718
Quintile 3	-0.009	0.234	0.070	0.326
Quintile 4	0.002	0.759	0.004	0.958
Quintile 5	0.003	0.707	0.155	0.040
**Lifestyle groups**				
Unfavorable group	Reference		Reference	
Average group	0.003	0.699	0.094	0.218
Favorable group	0.006	0.566	0.054	0.573
**Time**	0.012	0.000	0.037	<0.001
**Lifestyle groups*Time**				
Unfavorable*Time	Reference		Reference	
Average*Time	-0.001	0.001	-0.016	<0.001
Favorable*Time	-0.002	<0.001	-0.026	<0.001
**Depression scores at baseline**	-	-	0.763	<0.001
**Extreme heat**				
SU	0.039	0.411	0.772	0.182
TR	-0.054	0.375	0.525	0.477
WSDI	0.007	0.069	-0.047	0.309
TN90P	-0.003	0.294	0.039	0.319
TX90P	0.000	0.964	-0.043	0.324
TXX	0.016	0.324	0.413	0.038
TNX	0.010	0.687	-0.738	0.013

**Extreme cold**				
FD	0.036	0.644	-1.112	0.233
CSDI	0.004	0.190	0.089	0.030
ID	0.145	0.006	0.929	0.140
TN10P	-0.003	0.485	0.086	0.105
TX10P	-0.001	0.628	-0.014	0.616
TXN	0.085	0.050	1.702	0.001
TNN	-0.143	0.009	-1.242	0.062
**Other temperature**				
GSL	-0.020	0.519	-0.450	0.221
DTR	-0.091	0.121	-1.071	0.137
**Extreme precipitation**				
CDD	-0.002	0.825	-0.189	0.065
CWD	0.006	0.404	0.030	0.723
PRCPTOT	-0.080	0.144	-1.306	0.049
R10	0.035	0.197	0.552	0.093
R20	0.014	0.523	0.535	0.050
R95P	0.023	0.138	0.250	0.193
R99P	-0.004	0.589	0.118	0.156
SDII	-0.023	0.328	-0.756	0.009
RX1DAY	-0.011	0.256	-0.078	0.504
RX5DAY	0.016	0.100	0.255	0.035
**Other environmental indices**				
Cumulative sunshine	-0.001	0.966	-0.335	0.295
Air humidity	-0.006	0.776	0.245	0.334
Green space	0.000	0.508	-0.027	0.001
$PM_{2.5}$	0.000	0.048	-0.008	<0.001

Supplementary table 6. Interaction between healthy lifestyle and climate extremes with ADL scale and depression scale scores in middle-aged and older adults using Rubin's method.

Characteristics	ADL scale[a]		Depression scale[b]	
	Estimate	P value	Estimate	P value
**Extreme heat**				
Average group*SU	-0.118	0.289	0.413	0.696
Favorable group*SU	-0.043	0.743	0.079	0.950
Average group*TR	-0.102	0.457	1.393	0.289
Favorable group*TR	-0.122	0.475	-0.106	0.948
Average group*WSDI	0.001	0.896	-0.059	0.488
Favorable group*WSDI	0.006	0.604	-0.027	0.791
Average group*TN90P	0.000	0.959	0.043	0.545
Favorable group*TN90P	-0.004	0.619	0.013	0.875
Average group*TX90P	0.002	0.816	-0.029	0.711
Favorable group*TX90P	0.000	0.962	0.008	0.931
Average group*TXX	-0.003	0.927	0.082	0.002
Favorable group*TXX	-0.008	0.839	0.156	0.034
Average group*TNX	0.022	0.694	0.362	0.503
Favorable group*TNX	-0.001	0.992	0.578	0.365
**Extreme cold**				
Average group*FD	0.085	0.649	-0.698	0.693
Favorable group*FD	-0.081	0.714	0.625	0.763
Average group*CSDI	-0.001	0.934	0.012	0.042
Favorable group*CSDI	0.000	0.979	0.036	0.028
Average group*ID	0.099	0.020	0.052	0.964
Favorable group*ID	0.048	0.038	-0.845	0.540
Average group*TN10P	0.000	0.959	0.055	0.542
Favorable group*TN10P	0.003	0.783	0.013	0.904
Average group*TX10P	0.000	0.959	0.029	0.579
Favorable group*TX10P	0.003	0.783	0.013	0.833
Average group*TXN	-0.191	0.046	0.761	0.009
Favorable group*TXN	-0.252	0.029	1.985	0.030
Average group*TNN	-0.015	0.007	1.423	0.240
Favorable group*TNN	-0.013	0.043	2.076	0.151
**Other temperature**				
Average group*GSL	-0.052	0.474	-0.946	0.168
Favorable group*GSL	-0.070	0.412	-0.585	0.464
Average group*DTR	-0.134	0.305	1.078	0.393
Favorable group*DTR	-0.049	0.753	1.513	0.313
**Extreme precipitation**				
Average group*CDD	-0.010	0.579	-0.014	0.936

Favorable group*CDD	-0.019	0.397	0.089	0.680
Average group*CWD	0.038	0.121	-0.118	0.449
Favorable group*CWD	0.057	0.303	-0.011	0.951
Average group*PRCPTOT	-0.005	0.968	0.873	0.454
Favorable group*PRCPTOT	-0.033	0.819	0.472	0.733
Average group*R10	-0.085	0.166	-0.272	0.642
Favorable group*R10	-0.058	0.437	0.096	0.893
Average group*R20	-0.024	0.627	-0.367	0.439
Favorable group*R20	0.003	0.964	-0.272	0.627
Average group*R95P	0.013	0.708	-0.149	0.648
Favorable group*R95P	0.006	0.888	-0.250	0.516
Average group*R99P	-0.009	0.602	-0.041	0.794
Favorable group*R99P	-0.007	0.704	-0.033	0.859
Average group*SDII	0.042	0.441	-0.528	0.015
Favorable group*SDII	0.021	0.750	-0.872	0.023
Average group*RX1DAY	0.000	0.987	-0.024	0.905
Favorable group*RX1DAY	0.020	0.441	0.120	0.627
Average group*RX5DAY	-0.016	0.490	0.051	0.019
Favorable group*RX5DAY	-0.028	0.310	0.221	0.041

[a]Adjusted covariates of linear mixed effect model included lifestyle groups, climate extremes indexes, age, gender, area of residence, educational attainment, marital status, number of chronic diseases, annual per capita household consumption level, and other environmental indices.

[b]Adjusted covariates of linear mixed effect model included lifestyle groups, climate extremes indexes, depression score at baseline, age, gender, area of residence, educational attainment, marital status, number of chronic diseases, annual per capita household consumption level, and other environmental indices.

Supplementary table 7. Associations with climate extremes and ADL scale scores in a population stratified by healthy lifestyle using Rubin's method.

Characteristics	Unfavorable group		Average group		Favorable group	
	Estimate	P value	Estimate	P value	Estimate	P value
**Extreme heat**						
SU	0.034	0.791	0.034	0.580	0.109	0.263
TR	-0.227	0.127	-0.032	0.689	-0.132	0.313
WSDI	0.016	0.100	0.006	0.225	0.013	0.116
TN90P	-0.001	0.858	-0.002	0.602	-0.006	0.335
TX90P	-0.001	0.943	0.001	0.879	-0.002	0.811
TXX	0.042	0.310	0.013	0.533	0.036	0.292
TNX	0.054	0.394	0.005	0.876	0.047	0.346
**Extreme cold**						
FD	0.165	0.413	0.015	0.881	0.024	0.877
CSDI	0.009	0.314	0.004	0.416	0.004	0.552
ID	0.145	0.032	0.252	0.056	0.128	0.260
TN10P	-0.007	0.503	0.000	0.972	0.002	0.858
TX10P	-0.002	0.685	-0.001	0.784	-0.005	0.339
TXN	0.114	0.048	0.166	0.129	0.054	0.550
TNN	-0.177	0.016	-0.224	0.098	-0.110	0.331
**Other temperature**						
GSL	-0.043	0.594	-0.034	0.395	-0.027	0.657
DTR	-0.106	0.485	-0.092	0.225	-0.181	0.145
**Extreme precipitation**						
CDD	0.008	0.684	-0.005	0.640	-0.004	0.804
CWD	-0.011	0.547	0.007	0.461	0.011	0.455
PRCPTOT	0.022	0.873	-0.074	0.301	-0.072	0.530
R10	0.051	0.449	0.019	0.598	0.053	0.364
R20	0.000	0.999	0.016	0.582	0.003	0.947
R95P	0.046	0.273	0.016	0.435	0.035	0.282
R99P	-0.017	0.360	-0.001	0.901	-0.004	0.761
SDII	-0.056	0.356	-0.031	0.323	-0.023	0.634
RX1DAY	-0.013	0.604	-0.020	0.105	-0.014	0.509
RX5DAY	0.020	0.434	0.024	0.065	0.017	0.395

[a]Adjusted covariates of linear mixed effect model included age, gender, area of residence, educational attainment, marital status, number of chronic diseases, annual per capita household consumption level, and other environmental indices.

Supplementary table 8. Associations with climate extremes and depression scale scores in a population stratified by healthy lifestyle using Rubin's method.

Characteristics	Unfavorable group		Average group		Favorable group	
	Estimate[a]	P value	Estimate[a]	P value	Estimate[a]	P value
**Extreme heat**						
SU	0.963	0.204	0.945	0.410	0.120	0.893
TR	-0.142	0.883	-0.694	0.655	-0.441	0.715
WSDI	0.039	0.532	-0.129	0.173	-0.037	0.611
TN90P	0.004	0.944	0.095	0.201	-0.012	0.829
TX90P	-0.055	0.335	0.034	0.706	0.042	0.554
TXX	0.881	0.001	-0.100	0.804	0.183	0.565
TNX	-0.330	0.397	-1.443	0.013	0.326	0.474
**Extreme cold**						
FD	-1.128	0.356	-2.303	0.215	-0.517	0.720
CSDI	0.128	0.018	0.169	0.038	0.017	0.786
ID	2.262	0.006	-1.058	0.425	-1.052	0.306
TN10P	0.089	0.207	0.169	0.121	0.169	0.121
TX10P	-0.044	0.233	-0.019	0.748	-0.047	0.301
TXN	2.479	<0.001	1.736	0.097	0.785	0.337
TNN	-2.734	0.002	-0.672	0.613	-0.079	0.939
**Other temperature**						
GSL	-0.860	0.074	-0.394	0.583	0.622	0.269
DTR	-1.808	0.055	-1.071	0.468	-1.342	0.239
**Extreme precipitation**						
CDD	-0.197	0.143	-0.038	0.860	0.078	0.635
CWD	0.026	0.813	-0.204	0.219	-0.104	0.423
PRCPTOT	-0.385	0.657	0.253	0.850	0.582	0.577
R10	0.157	0.715	0.394	0.563	0.091	0.864
R20	0.554	0.122	0.863	0.111	0.095	0.821
R95P	0.271	0.278	-0.170	0.654	0.008	0.979
R99P	0.092	0.391	0.242	0.140	-0.045	0.729
SDII	-1.099	0.004	-0.604	0.297	-0.011	0.980
RX1DAY	-0.141	0.354	-0.313	0.191	-0.154	0.410
RX5DAY	0.540	0.001	-0.025	0.918	-0.151	0.416

[a]Adjusted covariates of linear mixed effect model included depression score at baseline, age, gender, area of residence, educational attainment, marital status, number of chronic diseases, annual per capita household consumption level, and other environmental indices.

Supplementary table 9. Associations with quintiles of climate extremes and disability in a population stratified by healthy lifestyle using Rubin's method.

Characteristics	Unfavorable group		Average group		Favorable group	
	HR(95%CI)[a]	P value	HR(95%CI)[a]	P value	HR(95%CI)[a]	P value
**Extreme heat**						
SU						
Quintile 5	Reference		Reference		Reference	
Quintile 4	0.675(0.402,1.136)	0.139	0.882(0.34,2.285)	0.796	0.966(0.316,2.954)	0.952
Quintile 3	0.555(0.302,1.021)	0.058	2.15(0.755,6.119)	0.152	1.304(0.369,4.609)	0.680
Quintile 2	0.683(0.337,1.383)	0.289	1.68(0.489,5.768)	0.410	2.389(0.533,10.704)	0.255
Quintile 1	0.566(0.247,1.298)	0.179	1.082(0.225,5.204)	0.921	0.899(0.15,5.392)	0.907
TR						
Quintile 5	Reference		Reference		Reference	
Quintile 4	0.949(0.554,1.625)	0.848	0.902(0.368,2.211)	0.821	1.199(0.424,3.393)	0.733
Quintile 3	1.581(0.963,2.596)	0.070	0.77(0.281,2.11)	0.612	1.227(0.384,3.919)	0.730
Quintile 2	1.2(0.654,2.201)	0.556	1.466(0.459,4.688)	0.519	2.121(0.539,8.35)	0.282
Quintile 1	0.994(0.5,1.978)	0.987	0.496(0.15,1.648)	0.253	0.996(0.2,4.956)	0.996
WSDI						
Quintile 5	Reference		Reference		Reference	
Quintile 4	0.869(0.522,1.445)	0.588	1.728(0.743,4.019)	0.204	1.907(0.606,6.001)	0.270
Quintile 3	0.837(0.503,1.394)	0.495	1.375(0.532,3.551)	0.510	1.86(0.5,6.924)	0.355
Quintile 2	0.869(0.51,1.481)	0.606	1.441(0.569,3.65)	0.441	2.279(0.667,7.79)	0.189
Quintile 1	0.546(0.303,0.983)	0.044	3.032(1.195,7.689)	0.020	0.949(0.254,3.547)	0.938
TN90P						
Quintile 5	Reference		Reference		Reference	
Quintile 4	1.168(0.681,2.003)	0.573	2.102(0.843,5.239)	0.111	1.022(0.327,3.196)	0.971
Quintile 3	1.313(0.662,2.604)	0.436	0.822(0.269,2.512)	0.731	1.523(0.386,6.003)	0.548
Quintile 2	0.996(0.464,2.137)	0.991	0.79(0.222,2.813)	0.717	0.842(0.174,4.063)	0.830
Quintile 1	2.119(0.885,5.076)	0.092	0.602(0.147,2.468)	0.481	0.973(0.175,5.398)	0.975
TX90P						
Quintile 5	Reference		Reference		Reference	
Quintile 4	0.895(0.516,1.552)	0.693	1.989(0.785,5.038)	0.147	1.094(0.323,3.712)	0.885
Quintile 3	1.052(0.575,1.925)	0.868	1.274(0.45,3.603)	0.649	0.778(0.23,2.638)	0.687
Quintile 2	1.017(0.529,1.954)	0.960	1.341(0.473,3.802)	0.581	1.082(0.274,4.267)	0.911
Quintile 1	0.88(0.385,2.01)	0.761	3.04(0.755,12.24)	0.118	0.976(0.159,5.994)	0.979
TXX						
Quintile 5	Reference		Reference		Reference	
Quintile 4	1.74(0.845,2.898)	0.133	1.846(0.757,4.503)	0.178	0.641(0.181,2.272)	0.491
Quintile 3	1.217(1.151,2.278)	0.038	2.17(0.772,6.1)	0.142	0.659(0.185,2.343)	0.519
Quintile 2	1.192(0.638,2.225)	0.582	3.037(1.06,8.7)	0.039	0.692(0.211,2.269)	0.543
Quintile 1	0.801(0.395,1.627)	0.540	1.514(0.433,5.297)	0.516	0.543(0.126,2.349)	0.414
TNX						
Quintile 5	Reference		Reference		Reference	

Quintile 4	0.865(0.533,1.403)	0.557	1.109(0.442,2.784)	0.825	1.394(0.488,3.985)	0.535
Quintile 3	0.668(0.388,1.148)	0.144	0.896(0.324,2.482)	0.833	0.326(0.095,1.111)	0.073
Quintile 2	0.655(0.362,1.184)	0.161	3.396(0.824,8.099)	0.106	0.783(0.209,2.942)	0.718
Quintile 1	0.806(0.43,1.511)	0.501	2.075(0.62,6.947)	0.236	2.847(0.769,10.54)	0.117
**Extreme cold**						
FD						
Quintile 5	Reference		Reference		Reference	
Quintile 4	0.606(0.323,1.135)	0.117	0.773(0.276,2.167)	0.625	1.14(0.324,4.013)	0.838
Quintile 3	0.703(0.338,1.465)	0.347	1.101(0.312,3.89)	0.881	1.261(0.319,4.982)	0.741
Quintile 2	0.961(0.427,2.16)	0.923	1.504(0.374,6.05)	0.566	2.241(0.555,9.045)	0.257
Quintile 1	0.936(0.403,2.174)	0.878	1.792(0.42,7.641)	0.431	1.05(0.232,4.755)	0.950
CSDI						
Quintile 5	Reference		Reference		Reference	
Quintile 4	1.549(0.896,2.677)	0.117	1.435(0.605,3.399)	0.412	1.447(0.446,4.691)	0.539
Quintile 3	1.309(0.717,2.389)	0.381	2.031(0.795,5.19)	0.139	1.147(0.338,3.899)	0.826
Quintile 2	1.181(0.609,2.29)	0.623	0.974(0.325,2.915)	0.962	0.307(0.071,1.328)	0.114
Quintile 1	1.24(0.657,2.338)	0.507	1.161(0.406,3.321)	0.781	0.472(0.117,1.9)	0.291
ID						
Quintile 5	Reference		Reference		Reference	
Quintile 4	0.636(0.276,1.465)	0.287	1.539(0.486,4.875)	0.463	1.569(0.349,7.055)	0.557
Quintile 3	1.031(0.444,2.393)	0.943	2.233(0.607,8.218)	0.227	0.69(0.159,2.992)	0.620
Quintile 2	0.631(0.301,1.325)	0.224	0.822(0.256,2.639)	0.742	0.497(0.122,2.024)	0.329
Quintile 1	0.751(0.315,1.79)	0.519	0.592(0.136,2.576)	0.484	0.295(0.055,1.585)	0.155
TN10P						
Quintile 5	Reference		Reference		Reference	
Quintile 4	0.502(0.286,1.181)	0.086	0.419(0.155,1.133)	0.087	0.335(0.114,1.381)	0.246
Quintile 3	0.986(0.508,1.912)	0.967	0.728(0.237,2.234)	0.579	0.545(0.131,2.269)	0.405
Quintile 2	1.201(0.547,2.635)	0.649	0.414(0.098,1.753)	0.231	0.325(0.061,1.727)	0.187
Quintile 1	0.275(0.101,0.746)	0.011	1.147(0.21,6.256)	0.874	0.213(0.022,2.077)	0.183
TX10P						
Quintile 5	Reference		Reference		Reference	
Quintile 4	0.456(0.274,1.159)	0.103	0.401(0.161,1.198)	0.149	0.698(0.238,2.043)	0.511
Quintile 3	0.659(0.384,1.133)	0.132	0.453(0.18,1.14)	0.093	0.599(0.163,2.21)	0.442
Quintile 2	0.748(0.384,1.457)	0.393	0.907(0.256,3.209)	0.879	1.207(0.233,6.243)	0.823
Quintile 1	0.584(0.309,1.103)	0.097	1.258(0.392,4.04)	0.700	0.656(0.17,2.533)	0.541
TXN						
Quintile 5	Reference		Reference		Reference	
Quintile 4	0.941(0.497,1.778)	0.850	0.691(0.234,2.041)	0.503	1.95(0.572,6.655)	0.286
Quintile 3	2.122(0.804,4.081)	0.124	0.703(0.232,2.13)	0.534	2.599(0.71,9.522)	0.149
Quintile 2	1.69(1.167,3.295)	0.023	3.208(1.118,9.209)	0.030	4.908(1.232,19.546)	0.024
Quintile 1	1.923(0.965,3.831)	0.063	2.496(0.806,7.725)	0.113	1.21(0.247,5.92)	0.814
TNN						
Quintile 5	Reference		Reference		Reference	
Quintile 4	0.914(0.537,1.556)	0.742	2.998(0.893,7.533)	0.120	0.553(0.187,1.632)	0.283

Quintile 3	0.571(0.305,1.066)	0.079	1.249(0.459,3.399)	0.663	0.345(0.094,1.258)	0.107
Quintile 2	0.407(0.2,0.831)	0.014	3.15(1.367,10.267)	0.037	0.273(0.059,1.261)	0.096
Quintile 1	0.905(0.447,1.831)	0.781	2.687(0.761,9.49)	0.125	0.64(0.131,3.124)	0.581
**Other temperature**						
GSL						
Quintile 5	Reference		Reference		Reference	
Quintile 4	0.722(0.383,1.365)	0.316	1.947(0.709,5.348)	0.196	1.603(0.462,5.56)	0.458
Quintile 3	0.356(0.178,0.712)	0.004	1.526(0.449,5.185)	0.499	1.171(0.243,5.637)	0.844
Quintile 2	0.423(0.203,0.883)	0.022	2.046(0.667,6.272)	0.210	0.33(0.085,1.289)	0.111
Quintile 1	0.602(0.284,1.279)	0.187	1.387(0.425,4.528)	0.588	0.59(0.147,2.367)	0.457
DTR						
Quintile 5	Reference		Reference		Reference	
Quintile 4	1.073(0.629,1.829)	0.796	1.004(0.372,2.71)	0.994	1.078(0.321,3.628)	0.903
Quintile 3	1.233(0.663,2.295)	0.508	0.802(0.251,2.567)	0.711	1.269(0.345,4.671)	0.720
Quintile 2	1.455(0.659,3.21)	0.353	0.797(0.191,3.33)	0.755	3.048(0.557,16.679)	0.199
Quintile 1	2.655(1.019,6.92)	0.046	0.739(0.131,4.176)	0.732	11.514(0.861,97.385)	0.125
**Extreme precipitation**						
CDD						
Quintile 5	Reference		Reference		Reference	
Quintile 4	0.819(0.451,1.489)	0.513	0.947(0.406,2.205)	0.899	0.321(0.099,1.043)	0.059
Quintile 3	1.062(0.575,1.963)	0.848	0.554(0.185,1.657)	0.291	0.306(0.094,0.998)	0.050
Quintile 2	1.339(0.708,2.532)	0.370	0.713(0.256,1.984)	0.517	0.432(0.123,1.508)	0.188
Quintile 1	1.728(0.828,3.606)	0.145	0.798(0.248,2.57)	0.705	0.289(0.057,1.466)	0.134
CWD						
Quintile 5	Reference		Reference		Reference	
Quintile 4	1.584(0.929,2.701)	0.091	1.506(0.539,4.206)	0.435	1.38(0.395,4.825)	0.614
Quintile 3	1.092(0.661,1.804)	0.732	1.122(0.449,2.806)	0.806	0.582(0.161,2.112)	0.411
Quintile 2	1.152(0.631,2.104)	0.645	1.173(0.437,3.146)	0.751	3.383(0.902,12.694)	0.071
Quintile 1	0.851(0.441,1.642)	0.630	0.875(0.288,2.66)	0.813	1.008(0.275,3.695)	0.990
PRCPTOT						
Quintile 5	Reference		Reference		Reference	
Quintile 4	1.133(0.61,2.103)	0.693	2.622(0.909,7.563)	0.074	1.048(0.284,3.865)	0.944
Quintile 3	1.394(0.632,3.075)	0.410	4.522(0.844,15.214)	0.115	2.452(0.394,15.246)	0.336
Quintile 2	1.359(0.56,3.298)	0.498	4.457(0.932,21.307)	0.061	5.534(0.708,43.269)	0.103
Quintile 1	3.499(0.788,10.954)	0.331	6.328(0.868,37.485)	0.442	7.621(0.491,118.281)	0.147
R10						
Quintile 5	Reference		Reference		Reference	
Quintile 4	2.476(0.815,4.66)	0.105	1.066(0.428,2.654)	0.891	0.868(0.241,3.12)	0.828
Quintile 3	1.178(0.593,2.341)	0.640	0.723(0.246,2.131)	0.557	0.339(0.077,1.489)	0.152
Quintile 2	0.809(0.378,1.73)	0.584	1.01(0.284,3.59)	0.988	0.136(0.022,1.363)	0.134
Quintile 1	0.735(0.34,1.59)	0.434	1.208(0.325,4.493)	0.778	0.197(0.034,1.132)	0.069

R20						
Quintile 5	Reference		Reference		Reference	
Quintile 4	1.139(0.662,1.96)	0.637	2.388(0.961,5.931)	0.061	0.426(0.136,1.339)	0.144
Quintile 3	1.042(0.59,1.839)	0.888	2.245(0.787,6.401)	0.130	0.718(0.174,2.959)	0.646
Quintile 2	0.864(0.433,1.723)	0.678	1.061(0.334,3.373)	0.920	0.617(0.137,2.785)	0.530
Quintile 1	0.92(0.436,1.939)	0.826	1.309(0.345,4.968)	0.692	0.289(0.065,1.292)	0.104
R95P						
Quintile 5	Reference		Reference		Reference	
Quintile 4	1.844(0.711,3.364)	0.146	0.615(0.247,1.533)	0.297	3.003(0.873,10.329)	0.081
Quintile 3	2.638(1.26,5.52)	0.010	0.212(0.075,0.6)	0.003	1.165(0.258,5.261)	0.842
Quintile 2	2.446(0.994,6.017)	0.051	0.515(0.152,1.743)	0.286	0.889(0.155,5.108)	0.896
Quintile 1	2.483(0.901,6.845)	0.079	0.559(0.118,2.656)	0.465	1.37(0.192,9.779)	0.754
R99P						
Quintile 5	Reference		Reference		Reference	
Quintile 4	0.691(0.368,1.295)	0.249	0.938(0.308,2.859)	0.910	0.826(0.231,2.948)	0.768
Quintile 3	1.263(0.653,2.443)	0.488	1.691(0.534,5.354)	0.372	0.508(0.12,2.156)	0.358
Quintile 2	0.69(0.332,1.437)	0.322	2.145(0.63,7.304)	0.222	1.595(0.426,5.968)	0.488
Quintile 1	0.945(0.437,2.045)	0.887	0.885(0.186,4.221)	0.878	0.836(0.192,3.637)	0.811
SDII						
Quintile 5	Reference		Reference		Reference	
Quintile 4	0.609(0.335,1.108)	0.104	0.958(0.342,2.685)	0.934	0.965(0.261,3.564)	0.957
Quintile 3	0.602(0.278,1.301)	0.197	0.689(0.218,2.181)	0.526	0.866(0.168,4.478)	0.864
Quintile 2	0.685(0.267,1.755)	0.430	0.392(0.089,1.738)	0.218	0.746(0.095,5.836)	0.780
Quintile 1	0.566(0.202,1.583)	0.278	0.184(0.031,1.092)	0.062	0.268(0.028,2.528)	0.250
RX1DAY						
Quintile 5	Reference		Reference		Reference	
Quintile 4	0.906(0.537,1.528)	0.710	1.24(0.493,3.119)	0.647	1.739(0.539,5.611)	0.355
Quintile 3	1.085(0.59,1.995)	0.792	1.764(0.641,4.856)	0.272	1.621(0.352,7.472)	0.535
Quintile 2	0.514(0.265,1.497)	0.149	1.202(0.354,4.077)	0.768	1.91(0.501,7.281)	0.343
Quintile 1	0.75(0.368,1.529)	0.429	2.166(0.559,8.401)	0.264	1.234(0.223,6.827)	0.809
RX5DAY						
Quintile 5	Reference		Reference		Reference	
Quintile 4	1.21(0.691,2.121)	0.505	1.438(0.546,3.784)	0.462	1.013(0.313,3.275)	0.983
Quintile 3	0.866(0.468,1.603)	0.647	1.057(0.391,2.854)	0.913	1.601(0.513,4.996)	0.417
Quintile 2	1.08(0.578,2.016)	0.810	1.144(0.386,3.388)	0.809	4.332(0.768,16.068)	0.328
Quintile 1	0.689(0.32,1.485)	0.342	0.599(0.179,2.007)	0.406	2.161(0.473,9.887)	0.320

[a]Adjusted covariates of the time-dependent Cox regression model included age, gender, area of residence, educational attainment, marital status, number of chronic diseases, annual per capita household consumption level, and other environmental indices.

Supplementary table 10. Associations with quintiles of climate extremes and depression in a population stratified by healthy lifestyle using Rubin's method.

Characteristics	Unfavorable group		Average group		Favorable group	
	HR(95%CI)[a]	P value	HR(95%CI)[a]	P value	HR(95%CI)[a]	P value
**Extreme heat**						
SU						
Quintile 5	Reference		Reference		Reference	
Quintile 4	0.749(0.457,1.227)	0.252	0.488(0.201,1.186)	0.113	1.068(0.354,3.226)	0.907
Quintile 3	0.863(0.491,1.516)	0.608	0.827(0.310,2.209)	0.705	0.631(0.151,2.638)	0.528
Quintile 2	0.557(0.287,1.082)	0.084	0.809(0.266,2.465)	0.710	0.797(0.146,4.335)	0.792
Quintile 1	0.192(0.044,0.833)	0.028	0.499(0.230,1.081)	0.078	0.238(0.03,1.876)	0.173
TR						
Quintile 5	Reference		Reference		Reference	
Quintile 4	0.909(0.561,1.471)	0.697	0.964(0.414,2.245)	0.933	0.961(0.283,3.271)	0.950
Quintile 3	1.871(1.181,2.964)	0.008	1.962(0.813,4.735)	0.134	4.474(0.846,14.865)	0.214
Quintile 2	0.924(0.527,1.622)	0.783	1.672(0.590,4.739)	0.334	3.131(0.717,13.671)	0.129
Quintile 1	0.999(0.541,1.844)	0.997	1.257(0.407,3.888)	0.691	3.816(0.722,20.178)	0.115
WSDI						
Quintile 5	Reference		Reference		Reference	
Quintile 4	1.135(0.703,1.833)	0.605	0.379(0.168,1.256)	0.220	1.028(0.307,3.451)	0.964
Quintile 3	0.697(0.425,1.143)	0.152	0.560(0.235,1.339)	0.193	0.730(0.210,2.537)	0.621
Quintile 2	1.338(0.796,2.247)	0.272	0.453(0.193,1.064)	0.069	0.736(0.208,2.603)	0.635
Quintile 1	0.908(0.53,1.555)	0.725	0.870(0.356,2.126)	0.761	0.641(0.164,2.499)	0.521
TN90P						
Quintile 5	Reference		Reference		Reference	
Quintile 4	1.89(1.137,3.143)	0.014	2.154(0.841,5.518)	0.110	1.352(0.385,4.746)	0.638
Quintile 3	2.264(1.198,4.279)	0.012	0.783(0.261,2.351)	0.663	0.539(0.104,2.788)	0.461
Quintile 2	2.122(1.057,4.263)	0.034	1.946(0.6,6.309)	0.267	0.418(0.063,2.762)	0.365
Quintile 1	3.058(1.391,6.72)	0.005	1.936(0.519,7.226)	0.325	0.223(0.027,1.815)	0.161
TX90P						
Quintile 5	Reference		Reference		Reference	
Quintile 4	0.775(0.436,1.376)	0.384	1.949(0.754,5.033)	0.168	3.345(0.953,11.736)	0.059
Quintile 3	0.584(0.315,1.083)	0.088	0.673(0.239,1.894)	0.453	2.384(0.611,9.306)	0.211
Quintile 2	0.547(0.323,0.926)	0.025	0.94(0.344,2.572)	0.905	5.765(0.877,28.232)	0.331
Quintile 1	0.341(0.16,0.728)	0.005	0.439(0.113,0.899)	0.033	6.681(0.97,45.995)	0.054
TXX						
Quintile 5	Reference		Reference		Reference	
Quintile 4	1.087(0.66,1.791)	0.743	3.188(0.925,7.669)	0.110	1.751(0.502,6.115)	0.380
Quintile 3	0.936(0.529,1.656)	0.819	3.037(0.87,8.623)	0.237	1.385(0.346,5.541)	0.645
Quintile 2	0.9(0.488,1.659)	0.735	1.21(0.413,3.545)	0.729	1.516(0.351,6.558)	0.577
Quintile 1	0.905(0.46,1.779)	0.772	1.596(0.479,5.314)	0.446	4.845(0.914,25.676)	0.064
TNX						
Quintile 5	Reference		Reference		Reference	

Quintile 4	1.156(0.734,1.823)	0.531	0.984(0.417,2.324)	0.971	0.385(0.126,1.172)	0.093
Quintile 3	0.786(0.477,1.296)	0.345	1.041(0.365,2.963)	0.941	1.311(0.399,4.311)	0.655
Quintile 2	0.771(0.458,1.298)	0.327	2.029(0.77,5.345)	0.152	0.801(0.208,3.089)	0.748
Quintile 1	1.375(0.754,2.509)	0.299	2.082(0.577,7.518)	0.263	1.162(0.284,4.758)	0.835
**Extreme cold**						
FD						
Quintile 5	Reference		Reference		Reference	
Quintile 4	1.212(0.69,2.128)	0.503	0.571(0.200,1.630)	0.295	0.339(0.081,1.421)	0.139
Quintile 3	0.819(0.414,1.621)	0.567	0.397(0.120,1.315)	0.131	0.480(0.105,2.185)	0.343
Quintile 2	1.148(0.524,2.513)	0.730	0.575(0.142,2.319)	0.436	0.606(0.111,3.320)	0.564
Quintile 1	0.729(0.323,1.646)	0.447	0.420(0.105,1.684)	0.221	0.208(0.036,1.203)	0.080
CSDI						
Quintile 5	Reference		Reference		Reference	
Quintile 4	1.442(0.877,2.371)	0.149	0.727(0.292,1.805)	0.491	0.864(0.233,3.211)	0.827
Quintile 3	1.125(0.662,1.91)	0.663	0.878(0.340,2.270)	0.788	1.189(0.334,4.238)	0.789
Quintile 2	1.027(0.572,1.847)	0.928	0.327(0.109,0.978)	0.046	0.596(0.112,3.177)	0.544
Quintile 1	1.39(0.762,2.535)	0.284	0.562(0.173,1.823)	0.337	0.240(0.047,1.233)	0.087
ID						
Quintile 5	Reference		Reference		Reference	
Quintile 4	0.588(0.294,1.176)	0.133	0.293(0.094,1.107)	0.133	2.176(0.422,11.215)	0.353
Quintile 3	0.672(0.334,1.353)	0.265	0.590(0.172,2.019)	0.400	7.362(0.988,49.823)	0.141
Quintile 2	0.596(0.307,1.157)	0.126	0.358(0.117,1.094)	0.072	1.747(0.324,9.419)	0.516
Quintile 1	0.228(0.102,0.506)	0.000	0.289(0.066,1.273)	0.101	1.503(0.203,11.157)	0.690
TN10P						
Quintile 5	Reference		Reference		Reference	
Quintile 4	0.507(0.203,1.268)	0.146	0.620(0.240,1.604)	0.324	0.331(0.100,1.097)	0.071
Quintile 3	0.688(0.371,1.277)	0.236	0.509(0.166,1.558)	0.237	0.544(0.129,2.302)	0.409
Quintile 2	0.897(0.444,1.812)	0.762	0.565(0.157,2.038)	0.383	0.491(0.095,2.549)	0.398
Quintile 1	0.585(0.36,0.951)	0.031	0.593(0.132,2.67)	0.496	0.25(0.027,2.292)	0.220
TX10P						
Quintile 5	Reference		Reference		Reference	
Quintile 4	1.166(0.725,1.876)	0.525	1.501(0.632,3.566)	0.358	1.34(0.346,5.197)	0.672
Quintile 3	0.996(0.598,1.659)	0.989	0.323(0.127,1.221)	0.118	0.602(0.142,2.548)	0.490
Quintile 2	0.634(0.321,1.255)	0.191	0.991(0.289,3.402)	0.989	1.541(0.258,9.204)	0.635
Quintile 1	1.228(0.665,2.267)	0.511	1.386(0.453,4.243)	0.568	1.007(0.212,4.774)	0.993
TXN						
Quintile 5	Reference		Reference		Reference	
Quintile 4	1.026(0.554,1.899)	0.935	1.025(0.363,2.900)	0.962	1.24(0.273,5.641)	0.781
Quintile 3	1.227(0.685,2.196)	0.492	0.444(0.147,1.342)	0.150	0.928(0.194,4.448)	0.925
Quintile 2	1.577(0.864,2.877)	0.138	1.028(0.377,2.798)	0.958	1.18(0.213,6.542)	0.849
Quintile 1	1.828(0.971,3.443)	0.062	1.958(0.675,5.675)	0.216	1.827(0.34,9.807)	0.482
TNN						
Quintile 5	Reference		Reference		Reference	
Quintile 4	0.791(0.487,1.286)	0.345	2.227(0.914,5.423)	0.078	0.416(0.123,1.408)	0.159

Quintile 3	0.625(0.361,1.083)	0.094	1.258(0.5,3.166)	0.626	0.495(0.127,1.939)	0.313
Quintile 2	0.872(0.448,1.697)	0.687	3.821(1.316,11.093)	0.014	0.72(0.148,3.499)	0.684
Quintile 1	1.497(0.751,2.986)	0.252	2.134(0.655,6.95)	0.208	0.525(0.093,2.972)	0.466
**Other temperature**						
GSL						
Quintile 5	Reference		Reference		Reference	
Quintile 4	1.003(0.566,1.778)	0.991	0.809(0.272,2.402)	0.702	0.474(0.11,2.041)	0.316
Quintile 3	0.918(0.487,1.73)	0.790	0.54(0.167,1.747)	0.304	0.376(0.074,1.908)	0.238
Quintile 2	0.671(0.349,1.289)	0.231	0.299(0.104,0.863)	0.026	0.103(0.022,0.494)	0.004
Quintile 1	0.661(0.337,1.297)	0.229	0.237(0.069,0.807)	0.021	0.125(0.025,0.625)	0.011
DTR						
Quintile 5	Reference		Reference		Reference	
Quintile 4	0.958(0.567,1.617)	0.871	1.265(0.546,2.93)	0.583	0.608(0.177,2.083)	0.428
Quintile 3	1.819(0.843,3.924)	0.127	2.593(0.913,7.368)	0.074	1.233(0.271,5.6)	0.786
Quintile 2	1.898(1.029,3.5)	0.040	2.576(0.688,9.649)	0.160	1.131(0.211,6.054)	0.886
Quintile 1	3.434(1.406,8.386)	0.007	8.768(1.654,46.487)	0.011	4.554(0.572,36.233)	0.152
**Extreme precipitation**						
CDD						
Quintile 5	Reference		Reference		Reference	
Quintile 4	0.493(0.293,0.827)	0.007	0.763(0.332,1.755)	0.525	0.813(0.224,2.954)	0.754
Quintile 3	0.416(0.237,0.733)	0.002	0.477(0.159,1.434)	0.188	1.055(0.267,4.166)	0.939
Quintile 2	0.234(0.13,0.42)	0.000	0.392(0.137,1.125)	0.082	0.786(0.212,2.91)	0.719
Quintile 1	0.177(0.091,0.345)	0.000	0.763(0.233,2.5)	0.655	1.16(0.235,5.722)	0.855
CWD						
Quintile 5	Reference		Reference		Reference	
Quintile 4	1.021(0.615,1.696)	0.935	3.287(0.816,8.88)	0.119	1.077(0.337,3.435)	0.901
Quintile 3	0.919(0.556,1.516)	0.740	0.907(0.376,2.187)	0.828	0.6(0.18,2.002)	0.406
Quintile 2	1.426(0.809,2.512)	0.219	1.596(0.545,4.672)	0.393	1.18(0.308,4.515)	0.809
Quintile 1	1.156(0.653,2.049)	0.618	1.472(0.492,4.41)	0.490	0.841(0.204,3.465)	0.811
PRCPTOT						
Quintile 5	Reference		Reference		Reference	
Quintile 4	0.463(0.257,1.134)	0.110	2.845(0.965,8.386)	0.058	0.642(0.154,2.669)	0.542
Quintile 3	0.803(0.376,1.717)	0.572	3.157(0.854,11.662)	0.085	1.346(0.179,10.124)	0.773
Quintile 2	0.952(0.385,2.355)	0.915	6.437(0.83,33.694)	0.127	1.39(0.14,13.846)	0.779
Quintile 1	1.592(0.538,4.715)	0.401	8.306(0.927,52.005)	0.224	0.983(0.071,13.633)	0.990
R10						
Quintile 5	Reference		Reference		Reference	
Quintile 4	1.403(0.816,2.413)	0.220	0.357(0.148,1.362)	0.222	0.748(0.203,2.755)	0.662
Quintile 3	1.115(0.591,2.104)	0.736	0.261(0.088,1.77)	0.315	1.299(0.307,5.492)	0.722
Quintile 2	0.525(0.256,1.076)	0.079	0.35(0.098,1.245)	0.105	0.518(0.092,2.919)	0.456
Quintile 1	0.819(0.404,1.659)	0.579	0.257(0.065,1.021)	0.054	1.719(0.302,9.787)	0.541
R20						

Quintile 5	Reference		Reference		Reference	
Quintile 4	0.912(0.558,1.49)	0.712	0.864(0.364,2.054)	0.741	0.863(0.21,3.545)	0.838
Quintile 3	1.72(0.961,3.077)	0.068	2.04(0.68,6.125)	0.204	2.263(0.564,9.071)	0.249
Quintile 2	1.033(0.524,2.038)	0.924	1.684(0.513,5.525)	0.390	0.861(0.175,4.24)	0.854
Quintile 1	0.807(0.371,1.753)	0.588	0.85(0.217,3.336)	0.816	1.769(0.307,10.202)	0.524
R95P						
Quintile 5	Reference		Reference		Reference	
Quintile 4	1.631(0.965,2.756)	0.068	1.32(0.499,3.493)	0.576	0.719(0.195,2.659)	0.621
Quintile 3	2.001(1.034,3.871)	0.039	0.645(0.217,1.92)	0.430	0.943(0.198,4.497)	0.942
Quintile 2	2.841(1.301,6.206)	0.009	0.648(0.191,2.193)	0.485	0.725(0.118,4.459)	0.728
Quintile 1	1.927(0.75,4.954)	0.173	1.001(0.216,4.637)	0.999	0.863(0.107,6.963)	0.890
R99P						
Quintile 5	Reference		Reference		Reference	
Quintile 4	0.846(0.472,1.518)	0.576	0.619(0.21,1.826)	0.385	0.311(0.082,1.183)	0.087
Quintile 3	0.973(0.533,1.775)	0.928	0.69(0.234,2.037)	0.502	0.983(0.244,3.964)	0.980
Quintile 2	1.338(0.687,2.606)	0.392	1.176(0.353,3.917)	0.792	0.673(0.171,2.657)	0.572
Quintile 1	1.08(0.559,2.088)	0.819	0.771(0.199,2.992)	0.708	0.484(0.109,2.149)	0.340
SDII						
Quintile 5	Reference		Reference		Reference	
Quintile 4	0.837(0.497,1.41)	0.504	2.585(0.928,7.195)	0.069	0.772(0.19,3.138)	0.717
Quintile 3	0.541(0.275,1.066)	0.076	2.035(0.642,6.446)	0.227	0.375(0.067,2.091)	0.263
Quintile 2	0.449(0.197,1.025)	0.057	2.102(0.509,8.687)	0.305	0.465(0.065,3.347)	0.447
Quintile 1	0.899(0.372,2.175)	0.814	1.013(0.192,5.341)	0.988	1.177(0.132,10.464)	0.884
RX1DAY						
Quintile 5	Reference		Reference		Reference	
Quintile 4	1.327(0.801,2.199)	0.272	1.966(0.778,4.966)	0.153	3.014(0.88,10.327)	0.079
Quintile 3	0.998(0.55,1.812)	0.995	0.732(0.264,2.03)	0.548	1.05(0.255,4.314)	0.946
Quintile 2	0.84(0.451,1.564)	0.583	1.554(0.477,5.061)	0.464	1.863(0.413,8.396)	0.418
Quintile 1	0.824(0.414,1.641)	0.582	0.773(0.204,2.933)	0.705	1.349(0.255,7.151)	0.725
RX5DAY						
Quintile 5	Reference		Reference		Reference	
Quintile 4	0.764(0.452,1.29)	0.313	0.682(0.264,1.761)	0.429	1.996(0.54,7.381)	0.300
Quintile 3	0.74(0.43,1.274)	0.277	0.903(0.384,2.122)	0.815	1.375(0.398,4.742)	0.615
Quintile 2	0.662(0.364,1.203)	0.176	0.704(0.265,1.871)	0.481	2.052(0.521,8.088)	0.304
Quintile 1	1.028(0.509,2.076)	0.938	1.2(0.417,3.458)	0.735	1.012(0.194,5.287)	0.989

[a]Adjusted covariates of the time-dependent Cox regression model included depression score at baseline, age, gender, area of residence, educational attainment, marital status, number of chronic diseases, annual per capita household consumption level, and other environmental indices.

Supplementary table 11. Associations with climate extremes and disability in a population stratified by healthy lifestyle using Rubin's method: subgroup analyses by age

Characteristics	＜60						≥60					
	Unfavorable group		Average group		Favorable group		Unfavorable group		Average group		Favorable group	
	HR(95%CI)[a]	P value	HR(95%CI)[a]	P value	HR(95%CI)[a]	P value	HR(95%CI)[a]	P value	HR(95%CI)[a]	P value	HR(95%CI)[a]	P value
**Extreme heat**												
SU	3.959(0.663,23.659)	0.131	0.531(0.024,11.959)	0.690	3.771(0.201,70.749)	0.375	10.312(0.55,193.256)	0.119	0.05(0,9.347)	0.262	57.347(0.372,131.946)	0.115
TR	1.918(0.211,17.422)	0.563	13.329(0.236,752.767)	0.208	18.685(0.56,623.694)	0.102	0.016(0,0.699)	0.032	58.284(0.137,234.344)	0.188	0.031(0,78.945)	0.385
WSDI	1.005(0.873,1.156)	0.949	1.063(0.797,1.42)	0.677	0.984(0.797,1.216)	0.883	1.418(1.107,1.817)	0.006	0.7(0.483,1.013)	0.058	0.726(0.457,1.155)	0.176
TN90P	0.851(0.753,0.961)	0.009	0.937(0.769,1.143)	0.522	0.945(0.788,1.133)	0.543	0.668(0.549,0.814)	0.000	1.07(0.793,1.445)	0.659	0.948(0.665,1.352)	0.770
TX90P	1.217(1.071,1.383)	0.003	0.903(0.703,1.161)	0.427	0.9(0.73,1.109)	0.322	1.06(0.843,1.333)	0.619	1.319(0.929,1.872)	0.122	1.436(0.97,2.124)	0.070
TXX	0.792(0.336,1.863)	0.593	1.694(0.95,3.023)	0.074	2.71(0.94,7.808)	0.065	2.9(1.013,8.3)	0.047	0.568(0.123,2.62)	0.469	0.485(0.082,2.86)	0.424
TNX	1.287(0.536,3.088)	0.573	1.2(0.222,6.479)	0.832	0.298(0.074,1.211)	0.091	3.02(0.645,14.15)	0.161	0.036(0.003,0.489)	0.012	0.064(0.006,0.727)	0.027
**Extreme cold**												
FD	0.126(0.006,2.574)	0.178	0.004(0,1.099)	0.054	1.209(0.011,135.162)	0.937	6.195(0.977,74.705)	0.051	4.33(0.003,15.479)	0.696	1.605(0,7.574)	0.909
CSDI	1.086(0.957,1.233)	0.202	1.057(0.838,1.333)	0.639	1.056(0.858,1.298)	0.608	1.012(0.825,1.241)	0.910	0.973(0.702,1.35)	0.872	1.154(0.808,1.648)	0.431
ID	7.843(1.071,57.439)	0.043	0.077(0.001,5.239)	0.233	4.149(0.163,105.53)	0.389	12.5(7.204,65.28)	0.001	0.615(0.002,170.805)	0.865	1.645(0.003,35.696)	0.880
TN10P	1.198(1.025,1.4)	0.023	1.257(0.931,1.695)	0.135	0.928(0.743,1.158)	0.507	0.749(0.579,0.97)	0.028	1.203(0.801,1.807)	0.372	1.073(0.726,1.587)	0.722
TX10P	1.022(0.935,1.118)	0.633	1.104(0.95,1.283)	0.198	1.047(0.915,1.198)	0.501	1.08(0.938,1.245)	0.284	1.005(0.796,1.268)	0.970	1.137(0.853,1.516)	0.380
TXN	8.185(1.655,40.477)	0.010	26.159(1.321,517.974)	0.032	0.415(0.043,4.017)	0.447	46.345(2.978,221.22)	0.006	0.3(0.005,17.594)	0.562	0.085(0.001,10.237)	0.313
TNN	0.327(0.04,2.666)	0.296	0.121(0.003,5.655)	0.281	1.906(0.109,33.429)	0.659	0.003(0,0.092)	0.001	7.056(0.03,33.306)	0.482	3.13(0.006,57.834)	0.721
**Other temperature**												
GSL	0.554(0.183,1.681)	0.297	8.208(0.797,84.51)	0.077	0.345(0.06,1.99)	0.234	7.811(1.058,57.672)	0.044	0.215(0.012,3.776)	0.293	0.294(0.016,5.292)	0.406
DTR	0.013(0.002,0.099)	0.000	1.052(0.031,35.665)	0.977	0.623(0.021,18.097)	0.783	0.057(0.002,1.965)	0.113	0.171(0.001,50.28)	0.542	0.038(0,19.871)	0.306

**Extreme precipitation**												
CDD	0.869(0.649,1.163)	0.345	0.933(0.559,1.558)	0.792	1.077(0.705,1.647)	0.731	1.03(0.601,1.764)	0.915	1.279(0.508,3.22)	0.602	3.177(0.864,9.486)	0.138
CWD	1.223(0.953,1.568)	0.113	0.717(0.453,1.134)	0.155	1.131(0.793,1.613)	0.496	0.81(0.541,1.213)	0.307	1.715(0.789,3.725)	0.173	1.135(0.577,2.235)	0.714
PRCPTOT	0.034(0.004,0.283)	0.002	6.485(0.151,277.913)	0.330	0.058(0.002,1.369)	0.078	11.507(0.448,295.782)	0.140	4.258(0.018,96.585)	0.603	0.531(0.001,198.324)	0.834
R10	2.584(0.956,6.987)	0.061	0.859(0.141,5.222)	0.869	3.951(0.871,17.917)	0.075	0.354(0.075,1.678)	0.191	0.187(0.012,2.952)	0.234	3.115(0.124,78.248)	0.490
R20	2.593(1.071,6.277)	0.035	2.096(0.439,10.009)	0.354	1.867(0.427,8.171)	0.407	0.266(0.08,0.885)	0.031	1.428(0.153,13.345)	0.755	0.949(0.083,10.91)	0.967
R95P	0.764(0.424,1.379)	0.372	0.413(0.147,1.156)	0.092	1.454(0.592,3.572)	0.414	0.861(0.38,1.949)	0.719	0.274(0.053,1.419)	0.123	0.901(0.15,5.43)	0.910
R99P	1.156(0.9,1.485)	0.256	0.723(0.462,1.133)	0.157	1.066(0.708,1.604)	0.761	1.204(0.831,1.744)	0.327	1.371(0.692,2.716)	0.366	0.592(0.274,1.278)	0.182
SDII	1.216(0.485,3.046)	0.677	0.952(0.184,4.931)	0.954	0.703(0.176,2.813)	0.618	1.869(0.494,7.07)	0.357	0.625(0.066,5.913)	0.682	1.269(0.089,18.067)	0.860
RX1DAY	0.993(0.697,1.416)	0.971	0.84(0.438,1.611)	0.600	1.261(0.724,2.194)	0.413	0.832(0.475,1.458)	0.520	0.441(0.187,1.038)	0.061	1.946(0.596,6.349)	0.270
RX5DAY	1.572(1.085,2.279)	0.017	1.808(0.921,3.551)	0.085	0.944(0.524,1.701)	0.849	0.559(0.311,1.005)	0.052	1.873(0.725,4.84)	0.195	1.013(0.33,3.105)	0.982

[a]Adjusted covariates of the time-dependent Cox regression model included age, gender, area of residence, educational attainment, marital status, number of chronic diseases, annual per capita household consumption level, and other environmental indices.

Supplementary table 12. Associations with climate extremes and disability in a population stratified by healthy lifestyle using Rubin's method: subgroup analyses by gender

Characteristics	Male						Female					
	Unfavorable group		Average group		Favorable group		Unfavorable group		Average group		Favorable group	
	HR(95%CI)[a]	P value	HR(95%CI)[a]	P value	HR(95%CI)[a]	P value	HR(95%CI)[a]	P value	HR(95%CI)[a]	P value	HR(95%CI)[a]	P value
**Extreme heat**												
SU	7.535(3.502,10.951)	0.007	2.566(0.346,19.009)	0.356	3.75(0.275,51.089)	0.321	8.629(0.835,89.142)	0.070	0.179(0,256.085)	0.643	0.477(0.033,6.962)	0.588
TR	17.092(3.009,45.255)	0.007	0.613(0.053,7.125)	0.696	11.825(0.479,291.692)	0.131	0.474(0.023,9.636)	0.627	10.805(0.002,86.577)	0.583	3.397(0.105,110.373)	0.491
WSDI	0.081(0.011,0.595)	0.013	1.097(0.934,1.289)	0.261	0.974(0.803,1.18)	0.785	1.026(0.849,1.24)	0.789	0.632(0.371,1.076)	0.091	1.064(0.841,1.345)	0.606
TN90P	0.062(0.012,0.331)	0.001	0.8(0.701,0.914)	0.001	0.888(0.749,1.053)	0.171	0.829(0.708,0.972)	0.021	1.407(0.846,2.339)	0.188	1.008(0.849,1.198)	0.925
TX90P	2.625(0.549,12.554)	0.227	1.224(1.052,1.425)	0.009	1.007(0.831,1.22)	0.946	1.115(0.945,1.316)	0.197	0.954(0.601,1.513)	0.840	0.981(0.795,1.211)	0.861
TXX	5.989(0.006,64.626)	0.608	1.79(0.902,3.555)	0.096	0.793(0.362,1.736)	0.562	2.095(0.99,4.432)	0.053	1.003(0.127,7.938)	0.998	1.255(0.518,3.038)	0.615
TNX	0.448(0.093,0.926)	0.048	1.251(0.461,3.395)	0.660	0.162(0.044,0.591)	0.006	1.933(0.602,6.207)	0.268	0.406(0.011,14.669)	0.623	0.437(0.11,1.735)	0.239
**Extreme cold**												
FD	0.002(0,0.008)	0.019	0.292(0.011,7.517)	0.458	0.997(0.014,69.812)	0.999	0.586(0.011,30.667)	0.791	1.493(0,77.67)	0.954	0.036(0,3.072)	0.143
CSDI	0.153(0.024,0.985)	0.048	1.082(0.94,1.245)	0.273	1.094(0.908,1.318)	0.346	1.033(0.872,1.223)	0.708	0.889(0.535,1.476)	0.648	1.111(0.914,1.35)	0.291
ID	2.292(1.597,6.736)	0.001	19.374(2.199,170.687)	0.008	4.849(0.271,86.721)	0.283	16.133(1.051,247.607)	0.046	0.031(0,120.756)	0.411	0.264(0.009,8.117)	0.446
TN10P	16.066(5.119,97.762)	0.003	1.035(0.863,1.242)	0.708	0.921(0.75,1.13)	0.431	1.109(0.911,1.351)	0.303	1.409(0.763,2.601)	0.273	1.076(0.85,1.362)	0.542
TX10P	0.384(0.125,1.183)	0.096	1.011(0.914,1.117)	0.836	1.059(0.937,1.196)	0.358	1.104(0.986,1.235)	0.085	0.884(0.62,1.262)	0.498	1.095(0.958,1.252)	0.181
TXN	13.492(0.001,126.725)	0.147	14.913(2.412,92.192)	0.004	0.319(0.045,2.27)	0.254	11.255(1.422,89.102)	0.022	21.754(0.033,116.268)	0.353	5.81(0.481,70.158)	0.166
TNN	3.656(0,42.512)	0.920	0.024(0.002,0.243)	0.002	2.695(0.187,38.793)	0.466	0.605(0.04,9.246)	0.718	0.257(0,521.1)	0.726	0.248(0.01,6.215)	0.396
**Other temperature**												
GSL	11.92(0.007,35.23)	0.168	0.58(0.162,2.07)	0.401	0.259(0.054,1.242)	0.091	2.163(0.487,9.614)	0.311	1.693(0.015,194.696)	0.828	1.202(0.212,6.8)	0.835

DTR	0.029(0,0.852)	0.003	0.025(0.002,0.286)	0.003	0.503(0.023,10.965)	0.662	0.019(0.001,0.243)	0.002	0.015(0,55.181)	0.317	1.086(0.048,24.403)	0.959
**Extreme precipitation**												
CDD	0.132(0.007,2.687)	0.188	0.891(0.638,1.245)	0.498	1.152(0.754,1.759)	0.513	0.876(0.586,1.312)	0.522	0.584(0.235,1.449)	0.246	1.163(0.744,1.817)	0.508
CWD	1.514(0.185,12.397)	0.699	1.106(0.84,1.457)	0.472	1.34(0.931,1.929)	0.115	1.058(0.766,1.462)	0.732	0.745(0.286,1.94)	0.546	1.023(0.71,1.473)	0.904
PRCPTOT	0(0,86.724)	0.130	0.085(0.009,0.846)	0.036	0.229(0.013,4.07)	0.315	0.675(0.044,10.456)	0.779	0.139(0,119.769)	0.567	0.511(0.023,11.559)	0.673
R10	7.427(2.965,96.466)	0.018	1.509(0.504,4.514)	0.462	1.425(0.356,5.707)	0.617	1.005(0.279,3.615)	0.994	3.427(0.095,123.924)	0.501	0.992(0.211,4.664)	0.992
R20	0.12(0,9.781)	0.700	1.171(0.447,3.066)	0.748	2.108(0.542,8.206)	0.282	1.399(0.475,4.12)	0.543	0.743(0.043,12.862)	0.838	1.996(0.522,7.637)	0.313
R95P	1.062(0.21,7.122)	0.161	0.796(0.404,1.569)	0.509	0.746(0.326,1.708)	0.488	0.801(0.398,1.613)	0.534	3.797(0.432,33.371)	0.229	0.723(0.298,1.759)	0.475
R99P	0.743(0.015,37.171)	0.882	1.297(0.974,1.727)	0.075	1.296(0.892,1.882)	0.174	1.056(0.778,1.433)	0.726	0.734(0.316,1.703)	0.472	0.745(0.507,1.095)	0.134
SDII	3.43(0.018,12.349)	0.367	1.803(0.626,5.193)	0.275	1.055(0.301,3.696)	0.933	0.962(0.323,2.865)	0.944	0.237(0.009,6.504)	0.394	0.905(0.23,3.559)	0.886
RX1DAY	0.106(0.001,9.01)	0.323	0.872(0.58,1.311)	0.510	0.872(0.536,1.418)	0.581	1.04(0.675,1.603)	0.857	1.496(0.455,4.922)	0.507	1.021(0.596,1.75)	0.939
RX5DAY	1.744(0.029,6.197)	0.791	1.127(0.741,1.716)	0.576	1.164(0.68,1.995)	0.579	1.141(0.721,1.804)	0.574	0.594(0.158,2.238)	0.442	1.606(0.908,2.84)	0.103

[a]Adjusted covariates of the time-dependent Cox regression model included age, area of residence, educational attainment, marital status, number of chronic diseases, annual per capita household consumption level, and other environmental indices.

Supplementary table 13. Associations with climate extremes and disability in a population stratified by healthy lifestyle using Rubin's method: subgroup analyses by area of residence

Characteristics	Urban community						Rural village					
	Unfavorable group		Average group		Favorable group		Unfavorable group		Average group		Favorable group	
	HR(95%CI)[a]	P value	HR(95%CI)[a]	P value	HR(95%CI)[a]	P value	HR(95%CI)[a]	P value	HR(95%CI)[a]	P value	HR(95%CI)[a]	P value
**Extreme heat**												
SU	35.681(2.727,466.838)	0.006	10.902(0.241,492.139)	0.219	1.965(0.025,157.458)	0.763	1.603(0.207,12.417)	0.651	1.234(0.039,39.194)	0.905	0.173(0.005,5.785)	0.327
TR	0.463(0.024,8.843)	0.609	79.62(0.848,181.085)	0.339	1.811(0.01,36.667)	0.824	0.226(0.015,3.408)	0.283	1.032(0.011,93.905)	0.989	5.228(0.024,13.04)	0.549
WSDI	0.984(0.794,1.218)	0.880	0.873(0.646,1.18)	0.377	0.957(0.631,1.451)	0.836	1.163(0.983,1.376)	0.078	0.922(0.711,1.196)	0.543	0.971(0.704,1.339)	0.859
TN90P	0.715(0.603,0.849)	0.000	0.857(0.666,1.103)	0.232	1.176(0.872,1.587)	0.288	0.818(0.706,0.947)	0.007	0.838(0.647,1.085)	0.179	0.895(0.707,1.133)	0.356
TX90P	1.063(0.864,1.306)	0.565	1.118(0.84,1.487)	0.445	0.836(0.582,1.201)	0.332	1.257(1.081,1.462)	0.003	1.177(0.905,1.532)	0.224	1.205(0.902,1.61)	0.207
TXX	0.68(0.218,2.12)	0.506	1.981(0.805,4.877)	0.137	2.475(0.502,12.208)	0.266	1.157(0.355,3.772)	0.809	1.822(0.903,3.677)	0.094	1.415(0.411,4.869)	0.582
TNX	2.203(0.535,9.066)	0.274	0.106(0.015,0.761)	0.026	0.115(0.008,1.64)	0.111	1.66(0.617,4.463)	0.316	0.786(0.124,4.971)	0.798	0.583(0.086,3.951)	0.581
**Extreme cold**												
FD	0.433(0.007,25.25)	0.687	0.455(0.001,200.25)	0.800	0.034(0,39.696)	0.348	8.435(0.22,23.322)	0.252	0.381(0,34.851)	0.788	0.022(0,19.966)	0.273
CSDI	1.012(0.858,1.193)	0.891	0.886(0.699,1.121)	0.313	1.017(0.756,1.368)	0.910	1.155(0.996,1.339)	0.056	1.244(0.941,1.645)	0.125	1.176(0.888,1.557)	0.257
ID	60.365(2.624,188.763)	0.010	2.909(0.046,184.701)	0.614	0.26(0,164.984)	0.682	41.114(4.82,90.715)	0.001	17.25(0.346,89.924)	0.153	0.253(0.003,24.515)	0.556
TN10P	0.918(0.714,1.18)	0.503	1.196(0.852,1.678)	0.300	1.252(0.83,1.887)	0.284	0.972(0.803,1.178)	0.774	0.919(0.683,1.237)	0.578	1.112(0.785,1.576)	0.551
TX10P	1.211(1.066,1.375)	0.003	0.969(0.797,1.178)	0.754	1.226(0.975,1.542)	0.081	0.981(0.885,1.086)	0.710	1.01(0.856,1.192)	0.905	1.013(0.846,1.214)	0.885
TXN	15.193(1.444,159.888)	0.023	0.431(0.021,9.01)	0.587	0.397(0.006,28.083)	0.671	18.696(2.925,119.486)	0.002	5.288(0.272,102.717)	0.271	9.229(0.337,52.955)	0.188
TNN	0.908(0.061,13.543)	0.944	1.671(0.045,61.388)	0.780	0.438(0.003,65.009)	0.746	0.016(0.001,0.253)	0.003	0.05(0.001,4.272)	0.187	0.646(0.004,99.334)	0.865
**Other temperature**												
GSL	0.629(0.116,3.401)	0.590	0.629(0.075,5.261)	0.669	14.275(0.486,49.393)	0.123	1.909(0.503,7.242)	0.342	0.172(0.016,1.836)	0.145	0.778(0.067,8.98)	0.841
DTR	0.128(0.003,4.728)	0.265	0.006(0,1.256)	0.061	0.369(0.001,14.376)	0.749	0.012(0.001,0.14)	0.000	0.16(0.003,9.457)	0.379	1.059(0.015,74.088)	0.979

**Extreme precipitation**												
CDD	0.427(0.256,1.212)	0.091	1.017(0.54,1.914)	0.959	0.899(0.401,2.019)	0.797	1.093(0.789,1.515)	0.593	1.089(0.637,1.86)	0.756	1.74(0.955,3.17)	0.070
CWD	1.347(0.874,2.076)	0.177	1.429(0.825,2.476)	0.203	0.576(0.277,1.197)	0.139	1.204(0.923,1.571)	0.172	1.068(0.665,1.715)	0.785	0.952(0.588,1.54)	0.842
PRCPTOT	0.010(0.000,1016)	0.086	0.073(0.001,7.009)	0.261	5.726(0.027,14)	0.524	0.264(0.029,2.429)	0.239	0.327(0.009,12.547)	0.548	2.385(0.039,146.219)	0.679
R10	5.231(0.943,24.008)	0.133	2.917(0.859,23.686)	0.116	0.944(0.076,11.656)	0.964	0.928(0.315,2.733)	0.892	2.985(0.424,20.989)	0.272	0.979(0.131,7.344)	0.984
R20	1.351(0.488,3.744)	0.563	0.901(0.167,4.853)	0.904	0.626(0.086,4.549)	0.644	1.009(0.352,2.892)	0.987	1.672(0.243,11.521)	0.602	2.166(0.346,13.547)	0.408
R95P	1.059(0.514,2.183)	0.877	1.708(0.499,5.844)	0.393	0.291(0.067,1.266)	0.100	0.582(0.288,1.174)	0.131	0.84(0.286,2.466)	0.751	0.707(0.213,2.345)	0.571
R99P	1.817(0.76,2.621)	0.101	1.391(0.798,2.425)	0.244	0.662(0.351,1.248)	0.202	0.944(0.715,1.248)	0.687	1.01(0.604,1.69)	0.970	0.706(0.432,1.155)	0.166
SDII	1.641(0.52,5.181)	0.398	0.436(0.082,2.327)	0.331	3.376(0.385,29.582)	0.272	3.106(0.969,9.956)	0.057	1.463(0.216,9.917)	0.697	0.969(0.136,6.917)	0.975
RX1DAY	0.689(0.429,1.107)	0.124	0.645(0.315,1.322)	0.231	0.92(0.371,2.285)	0.858	1.016(0.681,1.516)	0.937	1.071(0.543,2.113)	0.842	1.039(0.509,2.117)	0.917
RX5DAY	0.788(0.459,1.352)	0.387	1.701(0.727,3.979)	0.220	1.224(0.475,3.153)	0.676	1.434(0.938,2.192)	0.096	1.084(0.543,2.165)	0.819	1.564(0.719,3.401)	0.260

[a]Adjusted covariates of the time-dependent Cox regression model included age, gender, educational attainment, marital status, number of chronic diseases, annual per capita household consumption level, and other environmental indices.

Supplementary table 14. Associations with climate extremes and depression in a population stratified by healthy lifestyle using Rubin's method: subgroup analyses by age

Characteristics	＜60						≥60					
	Unfavorable group		Average group		Favorable group		Unfavorable group		Average group		Favorable group	
	HR(95%CI)[a]	P value	HR(95%CI)[a]	P value	HR(95%CI)[a]	P value	HR(95%CI)[a]	P value	HR(95%CI)[a]	P value	HR(95%CI)[a]	P value
**Extreme heat**												
SU	23.806(3.196,177.347)	0.002	14.083(0.476,416.847)	0.126	1.194(0.026,55.797)	0.375	0.463(0.01,22.523)	0.698	2.818(0.272,29.15)	0.385	0.016(0,4.493)	0.151
TR	13.28(1.056,166.949)	0.045	5.997(0.097,371.897)	0.395	0.797(0.004,7.073)	0.102	0.063(0,12.183)	0.304	0.44(0.024,7.91)	0.577	0.535(0,8.273)	0.866
WSDI	0.865(0.738,1.015)	0.075	1.205(0.955,1.52)	0.116	1.058(0.756,1.482)	0.883	0.926(0.675,1.272)	0.636	1.034(0.854,1.252)	0.732	1.835(0.188,2.836)	0.106
TN90P	0.759(0.662,0.87)	<0.001	0.907(0.722,1.14)	0.403	0.823(0.638,1.061)	0.543	0.832(0.66,1.05)	0.122	0.717(0.613,1.838)	0.500	1.043(0.727,1.495)	0.819
TX90P	1.306(1.133,1.505)	<0.001	1.165(0.913,1.487)	0.219	1.278(0.942,1.732)	0.322	1.435(1.068,1.929)	0.017	1.171(1.079,1.401)	0.004	1.039(0.728,1.485)	0.832
TXX	0.572(0.199,1.649)	0.301	1.154(0.602,2.211)	0.666	2.314(0.626,8.546)	0.065	4.376(1.894,10.112)	0.001	2.624(0.73,9.432)	0.139	0.267(0.054,1.313)	0.104
TNX	0.574(0.212,1.557)	0.276	0.464(0.09,2.405)	0.360	0.397(0.056,2.809)	0.091	0.564(0.071,4.465)	0.587	1.449(0.457,4.592)	0.528	0.984(0.107,9.067)	0.988
**Extreme cold**												
FD	2.779(0.107,72.111)	0.538	0.945(0.003,288.049)	0.984	0.027(0,20.228)	0.937	1.263(0.003,7.333)	0.941	3.227(0.079,131.03)	0.535	1.037(0.001,91.591)	0.992
CSDI	1.016(0.879,1.176)	0.827	1.048(0.817,1.343)	0.713	1.108(0.847,1.449)	0.608	1.149(0.879,1.502)	0.308	1.002(0.854,1.176)	0.982	1.201(0.855,1.688)	0.291
ID	2.707(0.334,21.909)	0.351	20.283(0.503,817.32)	0.110	67.703(0.848,104.395)	0.389	17.237(5.586,166.415)	0.006	20.099(7.073,46.474)	0.000	10.162(0.071,146.752)	0.359
TN10P	1.353(1.14,1.607)	0.001	1.169(0.893,1.53)	0.257	1.196(0.817,1.75)	0.507	0.957(0.7,1.308)	0.781	0.971(0.794,1.188)	0.776	0.875(0.555,1.379)	0.564
TX10P	0.943(0.851,1.044)	0.255	1.049(0.886,1.242)	0.582	0.948(0.787,1.143)	0.501	1.064(0.867,1.305)	0.553	0.934(0.831,1.051)	0.259	0.905(0.7,1.169)	0.445
TXN	7.502(1.186,47.474)	0.032	3.876(0.241,62.273)	0.339	23.469(0.656,89.684)	0.447	2.847(0.087,93.472)	0.557	1.283(0.138,11.973)	0.827	0.846(0.009,80.164)	0.943
TNN	0.043(0.004,0.439)	0.008	0.005(0,0.477)	0.023	0.069(0.002,2.546)	0.659	0.067(0.001,6.147)	0.241	0.621(0.045,8.652)	0.723	0.139(0,50.919)	0.512
**Other temperature**												
GSL	0.847(0.244,2.938)	0.794	0.076(0.008,0.734)	0.026	0.42(0.027,6.562)	0.234	1.076(0.12,9.608)	0.948	0.757(0.162,3.541)	0.723	0.498(0.031,8.025)	0.623
DTR	0.002(0,0.023)	<0.001	0.072(0.001,4.278)	0.207	0.016(0,1.896)	0.783	0.015(0,1.329)	0.066	0.019(0.001,0.346)	0.007	4.042(0.007,32.931)	0.667

**Extreme precipitation**												
CDD	1.255(0.898,1.756)	0.183	0.997(0.59,1.687)	0.992	1.273(0.673,2.411)	0.731	0.875(0.43,1.78)	0.712	1.574(0.911,2.451)	0.145	1.27(0.511,3.156)	0.607
CWD	0.885(0.661,1.186)	0.414	1.286(0.826,2.002)	0.266	0.874(0.49,1.559)	0.496	1.308(0.712,2.403)	0.387	1.331(0.941,1.884)	0.106	2.56(0.765,5.183)	0.109
PRCPTOT	0.317(0.028,3.541)	0.351	4.008(0.118,135.666)	0.440	5.445(0.064,63.389)	0.078	0.01(0,0.62)	0.029	0.176(0.015,2.085)	0.168	0.15(0.001,29.417)	0.481
R10	2.43(0.786,7.518)	0.123	0.469(0.083,2.635)	0.390	0.952(0.105,8.589)	0.075	0.338(0.043,2.641)	0.301	0.905(0.268,3.059)	0.873	1.067(0.066,17.189)	0.963
R20	0.548(0.213,1.41)	0.212	0.315(0.066,1.51)	0.149	1.589(0.258,9.78)	0.407	0.606(0.115,3.203)	0.556	1.662(0.581,4.757)	0.344	0.267(0.029,2.419)	0.240
R95P	0.571(0.297,1.1)	0.094	0.487(0.165,1.437)	0.192	1.006(0.298,3.391)	0.414	0.684(0.196,2.385)	0.551	0.64(0.313,1.305)	0.219	1.349(0.289,6.292)	0.703
R99P	1.019(0.764,1.359)	0.898	1.037(0.629,1.71)	0.887	1.065(0.628,1.808)	0.761	0.523(0.283,0.967)	0.039	1.282(0.927,1.773)	0.134	0.98(0.51,1.882)	0.952
SDII	1.075(0.399,2.899)	0.886	0.382(0.079,1.839)	0.230	0.131(0.021,0.841)	0.618	4.821(0.819,28.39)	0.082	2.519(0.818,7.763)	0.108	0.123(0.011,1.356)	0.087
RX1DAY	1.435(0.948,2.173)	0.088	0.68(0.355,1.305)	0.247	0.653(0.301,1.416)	0.413	1.417(0.658,3.048)	0.373	0.625(0.396,0.987)	0.044	0.514(0.189,1.396)	0.192
RX5DAY	1.022(0.677,1.543)	0.918	1.339(0.681,2.635)	0.398	1.4(0.634,3.091)	0.849	2.531(1.138,5.632)	0.023	1.233(0.773,1.966)	0.379	1.586(0.547,4.602)	0.396

[a]Adjusted covariates of the time-dependent Cox regression model included depression score at baseline, age, gender, area of residence, educational attainment, marital status, number of chronic diseases, annual per capita household consumption level, and other environmental indices.

Supplementary table 15. Associations with climate extremes and depression in a population stratified by healthy lifestyle using Rubin's method: subgroup analyses by gender

Characteristics	Male						Female					
	Unfavorable group		Average group		Favorable group		Unfavorable group		Average group		Favorable group	
	HR(95%CI)[a]	P value	HR(95%CI)[a]	P value	HR(95%CI)[a]	P value	HR(95%CI)[a]	P value	HR(95%CI)[a]	P value	HR(95%CI)[a]	P value
**Extreme heat**												
SU	0.903(0.053,15.246)	0.944	13.887(1.749,110.23)	0.013	6.922(0,75)	0.328	3.314(0.37,29.66)	0.284	0.099(0.004,2.292)	0.149	37.679(0.091,175.628)	0.238
TR	4.143(0.113,152.528)	0.440	5.426(0.421,69.894)	0.195	3.87(0,13.584)	0.157	1.92(0.118,31.206)	0.647	0.292(0.004,21.327)	0.574	0.013(0,14.45)	0.226
WSDI	1.181(0.961,1.45)	0.113	0.937(0.79,1.111)	0.453	0.044(0.002,1.923)	0.244	0.954(0.801,1.136)	0.599	1.345(1.031,1.754)	0.029	0.772(0.48,1.24)	0.284
TN90P	0.802(0.67,0.961)	0.017	0.742(0.643,0.856)	0.000	0.636(0.101,4)	0.630	0.765(0.661,0.885)	0.000	0.952(0.773,1.172)	0.642	1.099(0.904,1.624)	0.096
TX90P	1.277(1.043,1.564)	0.018	1.324(1.132,1.547)	0.000	3.11(0.279,34.629)	0.356	1.198(1.025,1.401)	0.023	1.198(0.952,1.508)	0.124	1.382(0.899,2.125)	0.140
TXX	0.93(0.392,2.207)	0.869	1.158(0.571,2.348)	0.685	0.597(0.002,26.89)	0.925	2.524(1.216,5.235)	0.013	0.781(0.281,2.168)	0.635	1.747(0.293,10.411)	0.540
TNX	0.331(0.079,1.378)	0.129	0.446(0.161,1.234)	0.120	1.527(0.002,13.567)	0.238	1.452(0.471,4.476)	0.516	0.759(0.183,3.157)	0.705	5.744(0.27,122.275)	0.263
**Extreme cold**												
FD	0.158(0.002,16.596)	0.437	5.105(0.17,153.255)	0.348	0(0,30.882)	0.284	0.695(0.021,22.608)	0.838	0.639(0.006,71.896)	0.853	0(0,21.712)	0.161
CSDI	1.108(0.898,1.367)	0.338	0.92(0.791,1.069)	0.276	1.025(0.128,8.206)	0.981	1.096(0.938,1.279)	0.249	1.123(0.91,1.385)	0.279	0.754(0.504,1.128)	0.169
ID	84.044(4.704,1501.467)	0.003	4.569(0.563,37.095)	0.155	2.047(0,10)	0.967	51.599(5.308,501.573)	0.001	31.731(1.237,813.907)	0.037	0.344(0,289.037)	0.756
TN10P	0.917(0.727,1.156)	0.463	1.384(1.146,1.671)	0.001	4.491(1.151,12.145)	0.040	1.031(0.857,1.24)	0.744	0.983(0.735,1.313)	0.905	2.61(0.469,4.64)	0.101
TX10P	1.045(0.911,1.199)	0.532	0.896(0.803,1)	0.050	0.125(0.016,1.949)	0.344	0.949(0.852,1.058)	0.347	1.011(0.87,1.175)	0.890	0.891(0.658,1.205)	0.452
TXN	3.323(0.364,30.361)	0.287	2.663(0.39,18.173)	0.317	3.288(0.092,13.182)	0.066	4.643(0.602,35.801)	0.141	6.188(0.356,107.696)	0.211	2.437(0.01,565.726)	0.749
TNN	0.033(0.001,0.732)	0.031	0.066(0.006,0.727)	0.026	6.837(0,40.855)	0.922	0.211(0.018,2.5)	0.217	0.011(0,1.215)	0.415	0.771(0.002,260.901)	0.930
**Other temperature**												
GSL	0.102(0.018,0.59)	0.011	0.636(0.167,2.419)	0.507	0(0,110.687)	0.324	0.642(0.163,2.525)	0.526	0.929(0.131,6.577)	0.941	0.881(0.024,32.385)	0.945
DTR	0.122(0.004,3.913)	0.235	0.001(0,0.018)	0.000	0(0,3.432)	0.060	0.029(0.002,0.343)	0.005	0(0,0.065)	0.006	0.323(0.007,15.153)	0.565

**Extreme precipitation**												
CDD	1.063(0.659,1.713)	0.803	1.27(0.884,1.823)	0.196	0.011(0,1.102)	0.055	1.609(1.091,2.372)	0.016	1.444(0.859,2.426)	0.166	0.492(0.194,1.249)	0.136
CWD	1.563(1.052,2.322)	0.027	1.053(0.772,1.437)	0.744	47.611(0.343,118.12)	0.125	0.999(0.737,1.354)	0.995	1.369(0.882,2.124)	0.161	0.811(0.323,2.041)	0.657
PRCPTOT	0.46(0.021,9.922)	0.620	0.143(0.013,1.567)	0.111	0.839(0.626,1.331)	0.144	0.666(0.055,8.112)	0.750	0.643(0.023,18.021)	0.795	0.07(0,17.56)	0.346
R10	0.257(0.057,1.154)	0.076	3.042(0.971,9.528)	0.056	2.582(0.899,4.166)	0.447	0.548(0.167,1.793)	0.320	0.724(0.134,3.904)	0.707	0.398(0.025,6.254)	0.512
R20	0.434(0.113,1.668)	0.224	1.188(0.452,3.125)	0.726	79.853(0.793,181.891)	0.602	0.842(0.298,2.382)	0.746	0.944(0.232,3.835)	0.936	0.48(0.047,4.899)	0.536
R95P	0.835(0.347,2.007)	0.687	0.603(0.303,1.2)	0.150	64.19(0.009,83.145)	0.277	0.533(0.27,1.053)	0.070	1.067(0.402,2.833)	0.896	0.112(0.016,1.275)	0.127
R99P	0.667(0.432,1.031)	0.068	1.162(0.85,1.587)	0.346	0.055(0.001,9.068)	0.265	1.047(0.775,1.413)	0.765	0.983(0.655,1.475)	0.933	1.054(0.49,2.268)	0.893
SDII	1.207(0.339,4.296)	0.772	0.711(0.246,2.054)	0.529	0.352(0,5.241)	0.895	4.709(1.646,13.468)	0.004	0.267(0.062,1.141)	0.075	2.634(0.168,41.406)	0.491
RX1DAY	1.143(0.653,2.001)	0.640	1.192(0.775,1.833)	0.425	17.522(0.399,76.927)	0.096	0.881(0.57,1.36)	0.567	0.555(0.305,1.012)	0.055	2.377(0.802,7.039)	0.118
RX5DAY	1.772(0.984,3.192)	0.056	1.218(0.793,1.87)	0.368	0.246(0.002,30.244)	0.568	0.994(0.641,1.541)	0.978	1.288(0.69,2.403)	0.427	1.302(0.44,3.852)	0.634

[a]Adjusted covariates of the time-dependent Cox regression model included depression score at baseline, age, area of residence, educational attainment, marital status, number of chronic diseases, annual per capita household consumption level, and other environmental indices.

Supplementary table 16. Associations with climate extremes and depression in a population stratified by healthy lifestyle using Rubin's method: subgroup analyses by area of residence

Characteristics	Urban community						Rural village					
	Unfavorable group		Average group		Favorable group		Unfavorable group		Average group		Favorable group	
	HR(95%CI)[a]	P value	HR(95%CI)[a]	P value	HR(95%CI)[a]	P value	HR(95%CI)[a]	P value	HR(95%CI)[a]	P value	HR(95%CI)[a]	P value
**Extreme heat**												
SU	17.58(1.722,179.528)	0.016	1.986(0.033,119.968)	0.743	0.168(0,73.064)	0.565	2.52(0.068,92.733)	0.615	1.147(0.143,9.215)	0.898	0.035(0.001,1.865)	0.098
TR	4.014(0.279,57.664)	0.307	0.028(0,4.342)	0.165	0.371(0,413.847)	0.782	8.993(0.095,47.266)	0.344	2.238(0.137,36.481)	0.572	0.237(0.001,64.82)	0.615
WSDI	0.955(0.784,1.163)	0.648	0.774(0.548,1.095)	0.148	1.08(0.669,1.743)	0.752	1.173(0.922,1.493)	0.194	1.017(0.863,1.198)	0.841	1.481(0.736,2.116)	0.231
TN90P	0.772(0.659,0.905)	0.001	0.953(0.734,1.238)	0.719	1.036(0.697,1.542)	0.861	0.75(0.578,0.973)	0.030	0.755(0.652,0.875)	0.000	0.832(0.64,1.082)	0.170
TX90P	1.105(0.914,1.336)	0.302	1.539(1.121,2.114)	0.008	1.203(0.747,0.997)	0.048	1.21(0.928,1.576)	0.159	1.4(0.897,1.623)	0.184	1.273(0.962,1.683)	0.091
TXX	3.537(1.398,8.946)	0.008	0.392(0.105,1.463)	0.163	0.664(0.083,5.296)	0.699	1.563(0.515,4.747)	0.431	1.261(0.627,2.533)	0.515	0.909(0.249,3.32)	0.885
TNX	0.78(0.229,2.652)	0.690	0.148(0.016,1.357)	0.091	0.082(0.004,1.685)	0.105	0.672(0.113,3.983)	0.661	1.064(0.386,2.935)	0.905	2.236(0.321,15.599)	0.417
**Extreme cold**												
FD	10.164(0.224,60.245)	0.233	11.659(0.195,28.144)	0.142	0.027(0,123.137)	0.402	0.044(0,30.743)	0.349	2.223(0.059,84.012)	0.666	2.484(0.002,19.834)	0.798
CSDI	0.903(0.768,1.061)	0.214	1.225(0.947,1.585)	0.121	1.279(0.91,1.797)	0.156	1.046(0.782,1.399)	0.763	1.058(0.913,1.226)	0.454	1.114(0.829,1.497)	0.473
ID	52.24(4.304,634.106)	0.002	60.055(0.628,140.585)	0.078	3.835(0.006,81.497)	0.680	35.292(1.074,159.773)	0.045	11.064(1.379,88.756)	0.024	12.173(1.233,123.162)	0.040
TN10P	1.116(0.894,1.392)	0.331	0.867(0.594,1.267)	0.462	1.197(0.724,1.98)	0.483	1.21(0.907,1.615)	0.195	1.148(0.951,1.385)	0.150	0.836(0.568,1.231)	0.366
TX10P	0.982(0.864,1.116)	0.784	1.185(0.973,1.444)	0.091	0.743(0.553,1.936)	0.447	0.919(0.771,1.095)	0.345	0.945(0.852,1.049)	0.291	1.049(0.862,1.276)	0.635
TXN	0.444(0.045,4.38)	0.487	3.249(0.09,116.718)	0.519	0.026(0,4.522)	0.166	9.365(0.45,195.054)	0.149	17.596(0.585,119.755)	0.203	17.839(0.866,91.095)	0.097
TNN	0.511(0.042,6.218)	0.599	0.161(0.003,9.439)	0.379	24.901(0.09,259.021)	0.262	0.004(0,0.543)	0.027	0.007(0,0.107)	0.000	0(0,0.006)	0.000
**Other temperature**												
GSL	0.293(0.057,1.509)	0.142	0.388(0.031,4.898)	0.464	1.294(0.035,47.299)	0.888	0.183(0.021,1.618)	0.127	1.778(0.482,6.555)	0.387	0.841(0.053,13.419)	0.903
DTR	0.018(0.001,0.452)	0.015	0.027(0,9.833)	0.230	0.016(0,37.818)	0.298	0.002(0,0.153)	0.005	0.004(0,0.055)	0.000	0.36(0.002,54.212)	0.690

**Extreme precipitation**												
CDD	1.946(1.226,3.09)	0.005	2.11(0.985,4.522)	0.055	0.998(0.346,2.878)	0.997	0.668(0.382,1.169)	0.158	1.107(0.785,1.56)	0.562	1.388(0.734,2.626)	0.313
CWD	0.715(0.497,1.031)	0.072	1.219(0.696,2.136)	0.488	2.083(0.724,5.997)	0.174	1.738(1.021,2.959)	0.042	1.339(0.997,1.797)	0.052	1.273(0.754,2.148)	0.366
PRCPTOT	0.692(0.041,11.577)	0.798	0.525(0.004,67.717)	0.795	0.223(0.001,77.819)	0.616	0.034(0.001,1.282)	0.068	0.031(0.003,1.309)	0.103	0.795(0.011,58.248)	0.917
R10	1.757(0.423,7.298)	0.438	0.65(0.077,5.523)	0.693	1.718(0.078,37.835)	0.732	1.053(0.142,7.779)	0.960	1.466(0.48,4.481)	0.502	0.588(0.066,5.204)	0.633
R20	0.36(0.133,0.971)	0.043	0.281(0.05,1.59)	0.151	0.9(0.088,9.251)	0.929	0.917(0.154,5.447)	0.924	2.668(0.918,7.758)	0.072	1.193(0.168,8.45)	0.860
R95P	0.577(0.268,1.241)	0.159	0.327(0.082,1.301)	0.113	1.148(0.194,6.8)	0.879	1.111(0.365,3.381)	0.853	0.685(0.338,1.389)	0.295	1.624(0.461,5.718)	0.450
R99P	1.176(0.802,1.723)	0.407	1.248(0.644,2.42)	0.512	0.975(0.459,2.072)	0.948	0.551(0.33,0.921)	0.023	1.058(0.801,1.398)	0.692	0.997(0.594,1.674)	0.992
SDII	3.212(1.059,9.74)	0.039	0.578(0.085,3.911)	0.574	0.313(0.02,4.85)	0.406	1.231(0.194,7.809)	0.825	2.207(0.697,6.988)	0.178	0.212(0.033,1.355)	0.101
RX1DAY	0.926(0.57,1.505)	0.757	0.862(0.387,1.922)	0.717	0.63(0.198,2.005)	0.434	2.128(1.078,4.201)	0.029	0.866(0.579,1.295)	0.482	0.452(0.214,1.255)	0.138
RX5DAY	0.964(0.583,1.596)	0.888	1.175(0.436,3.164)	0.749	1.056(0.338,3.304)	0.925	1.859(0.945,3.66)	0.073	1.29(0.839,1.984)	0.246	1.462(0.63,3.394)	0.376

[a]Adjusted covariates of the time-dependent Cox regression model included depression score at baseline, age, gender, educational attainment, marital status, number of chronic diseases, annual per capita household consumption level, and other environmental indices.

Supplementary table 17. Associations with climate extremes and disability in a population stratified by healthy lifestyle using Rubin's method by adjusting with the death as the competing risk and using the inverse probability weighting method.

Characteristics	Model1[a]						Model2[b]					
	Unfavorable group		Average group		Favorable group		Unfavorable group		Average group		Favorable group	
	HR(95%CI)[c]	P value	HR(95%CI)[c]	P value	HR(95%CI)[c]	P value	HR(95%CI)[c]	P value	HR(95%CI)[c]	P value	HR(95%CI)[c]	P value
**Extreme heat**												
SU	4.142(0.949,18.068)	0.059	1.413(0.127,15.69)	0.780	0.546(0.051,5.8)	0.620	3.039(0.366,25.226)	0.303	0.544(0.088,3.366)	0.512	2.836(0.293,27.448)	0.368
TR	0.57(0.084,3.852)	0.560	8.097(0.56,117.031)	0.120	4.852(0.153,153.823)	0.370	1.362(0.073,25.304)	0.836	0.373(0.036,3.903)	0.410	0.616(0.041,9.248)	0.726
WSDI	1.013(0.896,1.146)	0.830	0.929(0.775,1.112)	0.420	1.031(0.821,1.295)	0.790	1.075(0.907,1.273)	0.404	0.941(0.804,1.101)	0.448	1.079(0.897,1.298)	0.419
TN90P	0.869(0.788,0.958)	0.005	0.996(0.86,1.153)	0.960	1.06(0.913,1.23)	0.450	0.824(0.711,0.954)	0.010	0.979(0.877,0.984)	0.034	0.818(0.71,1.243)	0.055
TX90P	1.163(1.045,1.295)	0.006	1.019(0.871,1.191)	0.820	1.045(0.866,1.262)	0.650	1.18(1.004,1.386)	0.044	1.128(0.988,1.288)	0.074	1.005(0.863,1.17)	0.948
TXX	1.789(1.143,2.799)	0.011	0.961(0.514,1.796)	0.900	1.4(0.666,2.944)	0.370	1.061(0.545,2.067)	0.861	1.13(0.639,2)	0.674	1.453(0.684,3.088)	0.331
TNX	0.862(0.396,1.874)	0.710	0.312(0.096,0.81)	0.042	0.406(0.11,1.496)	0.180	0.29(0.106,0.794)	0.016	0.886(0.365,2.149)	0.788	1.46(0.411,5.177)	0.558
**Extreme cold**												
FD	0.358(0.03,4.315)	0.420	0.682(0.016,29.396)	0.840	0.017(0,1.129)	0.057	0.463(0.014,15.126)	0.665	0.158(0.006,4.091)	0.267	0.426(0.014,12.982)	0.624
CSDI	1.102(0.992,1.223)	0.071	1.002(0.842,1.194)	0.980	1.065(0.872,1.301)	0.530	1.182(1.005,1.39)	0.043	1.03(0.903,1.174)	0.663	0.971(0.822,1.147)	0.731
ID	6.153(0.866,43.693)	0.069	2.72(0.222,33.272)	0.430	0.282(0.011,7.417)	0.450	5.774(0.376,88.673)	0.208	6.209(0.669,57.584)	0.108	6.932(0.554,86.819)	0.133
TN10P	1.069(0.947,1.207)	0.280	1.05(0.886,1.244)	0.570	1.167(0.933,1.459)	0.180	0.942(0.78,1.138)	0.536	1.123(0.964,1.309)	0.137	1.024(0.842,1.246)	0.810
TX10P	1.057(0.984,1.135)	0.130	1.022(0.921,1.134)	0.680	1.125(0.999,1.266)	0.052	1.099(0.999,1.21)	0.053	0.971(0.884,1.065)	0.530	1.017(0.909,1.137)	0.768
TXN	14.57(3.508,60.505)	0.000	0.852(0.165,4.392)	0.850	4.326(0.547,34.209)	0.170	1.333(0.221,8.034)	0.754	3.475(0.749,16.124)	0.112	2.687(0.343,21.03)	0.346
TNN	0.126(0.023,0.709)	0.019	2.299(0.217,24.352)	0.490	0.381(0.019,7.517)	0.530	0.255(0.024,2.723)	0.258	0.161(0.022,1.157)	0.070	0.204(0.013,3.092)	0.252
**Other temperature**												
GSL	1.552(0.593,4.057)	0.370	0.416(0.102,1.698)	0.220	1.483(0.27,8.128)	0.650	0.346(0.092,1.305)	0.117	0.361(0.116,1.121)	0.078	0.359(0.085,1.524)	0.165
DTR	0.025(0.005,0.128)	0.000	0.288(0.023,3.53)	0.330	1.357(0.09,20.415)	0.830	0.353(0.026,4.739)	0.432	0.122(0.016,1.323)	0.142	0.118(0.006,2.326)	0.160

**Extreme precipitation**												
CDD	0.881(0.657,1.182)	0.400	1.062(0.758,1.489)	0.730	1.212(0.8,1.838)	0.360	1.477(0.992,2.2)	0.055	0.700(0.514,1.151)	0.123	0.937(0.593,1.482)	0.782
CWD	1.063(0.853,1.325)	0.580	1.12(0.826,1.518)	0.470	0.886(0.622,1.264)	0.500	0.966(0.732,1.275)	0.806	0.855(0.674,1.086)	0.199	0.973(0.719,1.316)	0.857
PRCPTOT	0.337(0.06,1.875)	0.210	0.552(0.055,5.579)	0.610	1.488(0.073,30.529)	0.800	0.938(0.076,11.617)	0.960	0.371(0.051,2.671)	0.325	0.462(0.027,7.841)	0.593
R10	1.002(0.439,2.288)	1.000	1.267(0.376,4.266)	0.700	0.991(0.248,3.965)	0.990	0.917(0.296,2.843)	0.880	0.913(0.356,2.343)	0.851	1.816(0.465,7.094)	0.391
R20	1.325(0.661,2.656)	0.430	1.013(0.327,3.141)	0.980	1.471(0.375,5.768)	0.580	2.171(0.739,6.38)	0.159	1.585(0.654,3.839)	0.308	1.077(0.36,3.218)	0.895
R95P	0.718(0.442,1.166)	0.180	1.051(0.511,2.165)	0.890	0.671(0.29,1.555)	0.350	0.886(0.426,1.841)	0.745	0.979(0.52,1.846)	0.949	1.395(0.679,2.868)	0.365
R99P	1.163(0.951,1.421)	0.140	1.075(0.799,1.446)	0.630	0.83(0.566,1.217)	0.340	1.119(0.829,1.511)	0.462	0.858(0.662,1.112)	0.246	0.988(0.714,1.368)	0.943
SDII	1.218(0.582,2.55)	0.600	0.709(0.254,1.975)	0.510	0.869(0.253,2.986)	0.820	0.958(0.334,2.748)	0.936	1.376(0.549,3.445)	0.496	0.732(0.249,2.153)	0.570
RX1DAY	0.927(0.697,1.232)	0.600	0.858(0.571,1.29)	0.460	0.833(0.534,1.298)	0.420	0.735(0.477,1.135)	0.165	1.003(0.717,1.403)	0.987	1.266(0.802,1.999)	0.310
RX5DAY	1.119(0.828,1.513)	0.460	1.122(0.715,1.761)	0.620	1.377(0.814,2.33)	0.230	1.125(0.728,1.739)	0.594	1.103(0.769,1.584)	0.594	0.891(0.565,1.405)	0.619

[a]Model 1 adjusted with the death as the competing risk.

[b]Model 2 using the inverse probability weighting method.

[c]Adjusted covariates of the time-dependent Cox regression model included age, gender, area of residence, educational attainment, marital status, number of chronic diseases, annual per capita household consumption level, and other environmental indices.

Supplementary table 18. Associations with climate extremes and depression in a population stratified by healthy lifestyle using Rubin's method by adjusting with the death as the competing risk and using the inverse probability weighting method.

Characteristics	Model1[a]						Model2[b]					
	Unfavorable group		Average group		Favorable group		Unfavorable group		Average group		Favorable group	
	HR(95%CI)[c]	P value	HR(95%CI)[c]	P value	HR(95%CI)[c]	P value	HR(95%CI)[c]	P value	HR(95%CI)[c]	P value	HR(95%CI)[c]	P value
**Extreme heat**												
SU	3.323(0.804,13.729)	0.097	0.775(0.059,10.174)	0.850	0.201(0.013,3.088)	0.250	0.481(0.095,2.435)	0.376	8.225(0.791,85.557)	0.078	17.563(0.653,472.69)	0.088
TR	1.581(0.239,10.467)	0.630	0.777(0.04,14.923)	0.870	0.18(0.003,12.515)	0.430	0.662(0.094,4.657)	0.679	2.408(0.116,50.081)	0.570	0.172(0.002,13.26)	0.427
WSDI	0.923(0.812,1.049)	0.220	1.081(0.899,1.3)	0.410	1.184(0.922,1.521)	0.190	1.123(0.983,1.284)	0.088	0.95(0.765,1.18)	0.645	0.977(0.774,1.234)	0.846
TN90P	0.798(0.725,0.878)	0.000	0.928(0.808,1.067)	0.290	0.979(0.823,1.165)	0.810	0.831(0.754,0.915)	0.000	0.619(0.528,0.726)	0.000	0.881(0.731,1.062)	0.183
TX90P	1.315(1.181,1.464)	0.000	1.22(1.037,1.436)	0.017	1.265(0.832,1.552)	0.124	1.299(1.165,1.449)	0.000	1.438(1.182,1.75)	0.000	1.026(0.835,1.26)	0.807
TXX	1.436(0.891,2.315)	0.140	1.243(0.631,2.449)	0.530	0.669(0.277,1.617)	0.370	1.181(0.711,1.962)	0.522	0.818(0.35,1.912)	0.642	1.556(0.52,4.661)	0.429
TNX	0.585(0.27,1.267)	0.170	0.699(0.217,2.256)	0.550	0.364(0.088,1.501)	0.160	1.012(0.468,2.187)	0.976	0.998(0.263,3.786)	0.997	0.708(0.132,3.799)	0.687
**Extreme cold**												
FD	1.378(0.138,13.72)	0.780	0.103(0.002,5.495)	0.260	0.405(0.004,41.103)	0.700	3.979(0.333,47.584)	0.275	5.548(0.125,247.095)	0.376	0.002(0.1,1.388)	0.121
CSDI	1.033(0.931,1.146)	0.540	1.019(0.86,1.208)	0.830	1.194(0.982,1.451)	0.075	1.136(1.013,1.272)	0.028	1.106(0.934,1.31)	0.243	1.05(0.837,1.318)	0.671
ID	6.7(1.288,34.84)	0.024	24.645(1.836,330.876)	0.016	21.512(0.797,580.571)	0.068	58.894(10.979,315.907)	0.000	18.197(0.879,306.804)	0.144	15.365(0.218,1081.994)	0.208
TN10P	1.155(1.024,1.302)	0.019	1.149(0.961,1.374)	0.130	0.995(0.789,1.256)	0.970	1.105(0.962,1.27)	0.158	1.042(0.854,1.27)	0.687	1.222(0.926,1.613)	0.156
TX10P	0.944(0.875,1.018)	0.140	0.984(0.876,1.106)	0.790	0.954(0.827,1.101)	0.520	0.912(0.843,0.987)	0.022	0.92(0.815,1.037)	0.173	0.909(0.768,1.075)	0.265
TXN	3.792(0.919,15.653)	0.065	3.284(0.469,22.985)	0.230	2.477(0.178,34.568)	0.500	6.496(1.545,27.313)	0.011	2.445(0.248,24.149)	0.444	1.899(0.101,35.521)	0.668
TNN	0.095(0.016,0.575)	0.010	0.074(0.005,1.053)	0.055	0.056(1.012,1.386)	0.011	0.106(0.017,0.679)	0.018	0.025(0.001,0.499)	0.016	2.141(0.054,85.287)	0.685
**Other temperature**												
GSL	1.027(0.419,2.517)	0.950	0.205(0.045,0.941)	0.041	0.994(0.136,7.284)	0.990	0.285(0.104,0.78)	0.014	0.848(0.197,3.658)	0.825	0.171(0.021,1.398)	0.100
DTR	0.011(0.002,0.056)	0.000	0.027(0.002,0.398)	0.008	0.203(0.009,4.802)	0.320	0.013(0.002,0.085)	0.000	0.002(0,0.042)	0.000	0.087(0.003,2.281)	0.143

**Extreme precipitation**												
CDD	1.507(1.131,2.008)	0.005	0.915(0.611,1.37)	0.670	1.212(0.737,1.993)	0.450	1.084(0.822,1.429)	0.567	1.697(0.976,2.846)	0.345	0.994(0.591,1.67)	0.981
CWD	1.042(0.827,1.314)	0.730	1.197(0.849,1.686)	0.310	1.261(0.795,2.001)	0.330	1.195(0.953,1.499)	0.122	1.198(0.842,1.706)	0.316	1.085(0.658,1.79)	0.748
PRCPTOT	0.565(0.107,2.97)	0.500	0.482(0.043,5.421)	0.550	1.339(0.063,28.663)	0.850	0.147(0.027,0.807)	0.027	0.044(0.003,0.718)	0.028	0.212(0.005,8.447)	0.410
R10	0.904(0.408,2.007)	0.810	0.328(0.099,1.089)	0.069	0.614(0.133,2.822)	0.530	0.682(0.305,1.522)	0.350	3.573(0.948,13.471)	0.060	0.626(0.088,4.438)	0.639
R20	1.068(0.551,2.071)	0.850	0.362(0.133,0.988)	0.047	0.772(0.222,2.687)	0.680	1.585(0.766,3.279)	0.215	1.031(0.303,3.516)	0.961	0.849(0.228,3.163)	0.807
R95P	0.617(0.384,0.992)	0.046	0.653(0.317,1.345)	0.250	0.91(0.37,2.238)	0.840	0.591(0.354,0.985)	0.044	0.402(0.19,0.853)	0.018	0.944(0.362,2.467)	0.907
R99P	1.111(0.9,1.372)	0.330	0.77(0.547,1.083)	0.130	0.908(0.605,1.363)	0.640	0.999(0.777,1.284)	0.994	0.963(0.663,1.401)	0.845	1.067(0.653,1.743)	0.796
SDII	1.568(0.755,3.258)	0.230	1.547(0.559,4.282)	0.400	0.27(0.07,1.035)	0.056	1.223(0.578,2.587)	0.598	1.496(0.484,4.622)	0.484	2.32(0.506,10.643)	0.279
RX1DAY	0.85(0.638,1.13)	0.260	0.983(0.629,1.537)	0.940	0.662(0.379,1.157)	0.150	0.652(0.461,0.923)	0.016	2.207(1.277,3.814)	0.005	0.709(0.348,1.446)	0.344
RX5DAY	1.012(0.756,1.354)	0.940	1.465(0.883,2.432)	0.140	1.403(0.799,2.462)	0.240	2.221(1.613,3.057)	0.000	1.033(0.633,1.687)	0.896	0.868(0.451,1.673)	0.673

[a]Model 1 adjusted with the death as the competing risk.

[b]Model 2 using the inverse probability weighting method.

[c]Adjusted covariates of the time-dependent Cox regression model included depression score at baseline, age, gender, area of residence, educational attainment, marital status, number of chronic diseases, annual per capita household consumption level, and other environmental indices.

Supplementary table 19. Associations of healthy lifestyle and climate extremes with cognitive decline in middle-aged and older adults using Rubin's method.

Characteristics	Estimate[a]	Standard Error	T value	P value
**Lifestyle groups**				
Unfavorable group	Reference			
Average group	0.018	0.019	0.944	0.345
Favorable group	0.038	0.024	1.557	0.119
**Time**	-0.002	0.001	-2.967	0.003
**Lifestyle groups*Time**				
Unfavorable*Time	Reference			
Average*Time	0.001	0.000	3.330	0.001
Favorable*Time	0.002	0.000	3.931	<0.001
**Extreme heat**				
SU	-0.237	0.102	-2.324	0.020
TR	0.177	0.130	1.360	0.174
WSDI	-0.005	0.008	-0.595	0.552
TN90P	0.012	0.007	1.755	0.079
TX90P	0.003	0.008	0.348	0.728
TXX	-0.081	0.035	-2.306	0.021
TNX	-0.071	0.052	-1.360	0.174
**Extreme cold**				
FD	-0.102	0.165	-0.622	0.534
CSDI	0.000	0.007	-0.034	0.973
ID	-0.184	0.111	-1.656	0.098
TN10P	0.000	0.009	0.015	0.988
TX10P	-0.006	0.005	-1.094	0.274
TXN	-0.148	0.092	-1.599	0.110
TNN	0.230	0.118	1.953	0.051
**Other temperature**				
GSL	0.021	0.018	1.143	0.253
DTR	0.001	0.015	0.048	0.962
**Extreme precipitation**				
CDD	0.080	0.065	1.231	0.218
CWD	0.219	0.118	1.862	0.063
PRCPTOT	0.518	0.255	2.034	0.042
R10	-0.060	0.048	-1.243	0.214
R20	-0.034	0.034	-1.012	0.312
R95P	-0.006	0.015	-0.425	0.671
R99P	-0.038	0.051	-0.744	0.457
SDII	0.157	0.127	1.235	0.217
RX1DAY	0.006	0.021	0.280	0.779
RX5DAY	0.022	0.021	1.027	0.305

[a]Adjusted covariates of linear mixed effect model included MMSE score at baseline, age, gender, area of residence, educational attainment, marital status, number of chronic diseases, annual per capita household consumption level, and other environmental indices.

Supplementary table 20. Interaction between healthy lifestyle and climate extremes with cognitive decline in middle-aged and older adults using Rubin's method..

Characteristics	MMSE score[a]		A standardized z-score[a]	
	Estimate	P value	Estimate	P value
**Extreme heat**				
Average group*SU	-1.213	0.011	-0.368	0.019
Favorable group*SU	-1.527	0.026	-0.448	0.038
Average group*TR	-1.050	0.330	-0.316	0.332
Favorable group*TR	-1.918	0.140	-0.582	0.138
Average group*WSDI	0.034	0.625	0.009	0.662
Favorable group*WSDI	-0.013	0.874	-0.005	0.844
Average group*TN90P	-0.003	0.964	-0.001	0.968
Favorable group*TN90P	0.021	0.744	0.007	0.740
Average group*TX90P	0.018	0.782	0.006	0.757
Favorable group*TX90P	-0.020	0.787	-0.006	0.799
Average group*TXX	-0.128	0.032	-0.039	0.029
Favorable group*TXX	-0.262	0.043	-0.080	0.039
Average group*TNX	0.215	0.627	0.065	0.627
Favorable group*TNX	0.386	0.450	0.109	0.479
**Extreme cold**				
Average group*FD	0.302	0.835	0.087	0.842
Favorable group*FD	0.195	0.907	0.065	0.896
Average group*CSDI	-0.032	0.602	-0.010	0.598
Favorable group*CSDI	-0.044	0.540	-0.013	0.553
Average group*ID	0.710	0.444	0.220	0.433
Favorable group*ID	0.220	0.841	0.073	0.825
Average group*TN10P	-0.005	0.947	-0.001	0.951
Favorable group*TN10P	0.014	0.872	0.004	0.864
Average group*TX10P	0.009	0.824	0.003	0.811
Favorable group*TX10P	0.034	0.486	0.010	0.488
Average group*TXN	-1.113	0.038	-0.342	0.013
Favorable group*TXN	-0.294	0.043	-0.096	0.046
Average group*TNN	0.590	0.550	0.190	0.525
Favorable group*TNN	0.284	0.806	0.093	0.791
**Other temperature**				
Average group*GSL	-0.332	0.554	-0.106	0.530
Favorable group*GSL	-0.209	0.742	-0.062	0.747
Average group*DTR	0.617	0.549	0.191	0.539
Favorable group*DTR	-0.034	0.977	-0.018	0.961
**Extreme precipitation**				
Average group*CDD	0.265	0.069	0.081	0.065

Favorable group*CDD	0.233	0.176	0.072	0.170
Average group*CWD	-0.034	0.793	-0.010	0.798
Favorable group*CWD	-0.108	0.461	-0.032	0.462
Average group*PRCPTOT	1.096	0.029	0.339	0.037
Favorable group*PRCPTOT	0.966	0.033	0.306	0.020
Average group*R10	-0.264	0.580	-0.077	0.595
Favorable group*R10	-0.203	0.721	-0.057	0.740
Average group*R20	0.519	0.181	0.162	0.168
Favorable group*R20	0.465	0.302	0.146	0.283
Average group*R95P	-0.091	0.733	-0.027	0.733
Favorable group*R95P	0.114	0.710	0.036	0.696
Average group*R99P	0.075	0.561	0.022	0.578
Favorable group*R99P	0.048	0.748	0.013	0.774
Average group*SDII	0.334	0.435	0.101	0.435
Favorable group*SDII	0.000	1.000	-0.002	0.990
Average group*RX1DAY	0.009	0.957	0.005	0.926
Favorable group*RX1DAY	0.056	0.777	0.019	0.750
Average group*RX5DAY	0.097	0.594	0.030	0.581
Favorable group*RX5DAY	0.039	0.853	0.015	0.817

[a]Adjusted covariates of linear mixed effect model included lifestyle groups, climate extremes indexes, MMSE score at baseline, age, gender, area of residence, educational attainment, marital status, number of chronic diseases, annual per capita household consumption level, and other environmental indices.

Supplementary table 21. Associations with climate extremes and MMSE scores in a population stratified by healthy lifestyle using Rubin's method.

Characteristics	Unfavorable group		Average group		Favorable group	
	Estimate[a]	P value	Estimate[a]	P value	Estimate[a]	P value
**Extreme heat**						
SU	-0.938	0.028	-0.963	0.248	-0.935	0.218
TR	0.707	0.197	0.546	0.575	0.892	0.384
WSDI	0.014	0.685	-0.023	0.712	-0.117	0.062
TN90P	0.026	0.377	0.073	0.177	0.018	0.713
TX90P	0.032	0.315	-0.029	0.641	0.024	0.694
TXX	-0.351	0.018	-0.171	0.525	-0.214	0.424
TNX	-0.361	0.102	-0.209	0.618	-0.016	0.966
**Extreme cold**						
FD	-0.099	0.886	-1.362	0.302	-0.842	0.492
CSDI	-0.004	0.894	0.025	0.666	-0.036	0.498
ID	-0.672	0.144	-0.480	0.574	-0.248	0.777
TN10P	0.034	0.389	-0.082	0.241	-0.023	0.747
TX10P	-0.039	0.061	0.023	0.574	0.011	0.782
TXN	-0.862	0.029	-0.094	0.895	-0.115	0.868
TNN	0.736	0.143	0.234	0.792	1.155	0.190
**Other temperature**						
GSL	0.268	0.325	0.333	0.523	0.215	0.650
DTR	0.015	0.978	1.759	0.081	1.149	0.238
**Extreme precipitation**						
CDD	0.013	0.860	0.102	0.449	0.122	0.382
CWD	-0.016	0.799	0.052	0.658	0.041	0.710
PRCPTOT	1.080	0.028	0.304	0.741	-0.408	0.644
R10	-0.607	0.013	-0.658	0.137	-0.491	0.275
R20	-0.393	0.052	0.019	0.961	0.326	0.361
R95P	-0.186	0.187	-0.107	0.697	0.101	0.687
R99P	-0.035	0.567	-0.013	0.915	0.009	0.932
SDII	-0.130	0.544	0.000	1.000	0.004	0.992
RX1DAY	-0.004	0.962	0.042	0.793	0.088	0.580
RX5DAY	0.117	0.188	0.021	0.902	0.006	0.970

[a]Adjusted covariates of linear mixed effect model included MMSE score at baseline, age, gender, area of residence, educational attainment, marital status, number of chronic diseases, annual per capita household consumption level, and other environmental indices.

Supplementary table 22. Associations with quintiles of climate extremes and cognitive decline in a population stratified by healthy lifestyle using Rubin's method.

Characteristics	Unfavorable group		Average group		Favorable group	
	Estimate[a]	P value	Estimate[a]	P value	Estimate[a]	P value
**Extreme heat**						
SU						
Quintile 5	Reference		Reference		Reference	
Quintile 4	-0.041	0.626	0.022	0.612	0.024	0.753
Quintile 3	0.044	0.440	0.076	0.113	0.129	0.145
Quintile 2	0.050	0.058	0.038	0.515	0.117	0.282
Quintile 1	0.070	0.022	0.083	0.194	0.118	0.337
TR						
Quintile 5	Reference		Reference		Reference	
Quintile 4	-0.010	0.905	-0.008	0.851	-0.039	0.638
Quintile 3	0.000	0.998	0.015	0.702	0.039	0.627
Quintile 2	-0.035	0.737	-0.009	0.848	0.024	0.799
Quintile 1	-0.043	0.679	0.003	0.958	-0.013	0.905
WSDI						
Quintile 5	Reference		Reference		Reference	
Quintile 4	0.064	0.412	0.009	0.816	0.037	0.585
Quintile 3	-0.028	0.732	-0.013	0.747	-0.022	0.777
Quintile 2	0.025	0.770	-0.031	0.458	0.029	0.702
Quintile 1	0.014	0.874	-0.003	0.948	0.072	0.366
TN90P						
Quintile 5	Reference		Reference		Reference	
Quintile 4	0.059	0.518	-0.036	0.413	-0.041	0.611
Quintile 3	0.094	0.369	-0.019	0.709	-0.030	0.756
Quintile 2	0.080	0.463	-0.101	0.072	-0.102	0.334
Quintile 1	0.095	0.432	-0.059	0.347	-0.080	0.468
TX90P						
Quintile 5	Reference		Reference		Reference	
Quintile 4	-0.038	0.677	-0.008	0.864	0.079	0.352
Quintile 3	-0.084	0.389	0.044	0.379	-0.014	0.881
Quintile 2	-0.027	0.781	0.062	0.238	0.028	0.769
Quintile 1	-0.043	0.730	-0.030	0.638	-0.052	0.669
TXX						
Quintile 5	Reference		Reference		Reference	
Quintile 4	0.017	0.832	-0.001	0.971	0.109	0.172
Quintile 3	-0.018	0.850	0.038	0.423	0.187	0.031
Quintile 2	0.011	0.011	-0.006	0.911	0.115	0.227
Quintile 1	0.024	0.004	0.097	0.087	0.066	0.535
TNX						
Quintile 5	Reference		Reference		Reference	

Quintile 4	-0.001	0.987	0.009	0.825	0.051	0.477
Quintile 3	0.057	0.520	-0.004	0.925	-0.021	0.794
Quintile 2	0.085	0.327	-0.003	0.953	0.017	0.848
Quintile 1	0.050	0.655	0.042	0.401	0.051	0.585
**Extreme cold**						
FD						
Quintile 5	Reference		Reference		Reference	
Quintile 4	-0.026	0.759	0.000	0.992	-0.042	0.599
Quintile 3	-0.008	0.940	0.021	0.677	-0.053	0.577
Quintile 2	-0.182	0.144	-0.005	0.932	-0.069	0.519
Quintile 1	0.020	0.866	0.035	0.559	0.018	0.867
CSDI						
Quintile 5	Reference		Reference		Reference	
Quintile 4	-0.048	0.559	0.022	0.602	-0.169	0.143
Quintile 3	-0.072	0.415	0.012	0.790	-0.153	0.167
Quintile 2	-0.081	0.421	0.016	0.753	-0.098	0.310
Quintile 1	-0.086	0.383	0.049	0.318	-0.061	0.522
ID						
Quintile 5	Reference		Reference		Reference	
Quintile 4	0.022	0.841	-0.028	0.637	-0.225	0.061
Quintile 3	0.069	0.567	-0.017	0.775	-0.196	0.111
Quintile 2	-0.008	0.940	0.011	0.843	-0.127	0.239
Quintile 1	0.124	0.371	-0.011	0.867	-0.162	0.202
TN10P						
Quintile 5	Reference		Reference		Reference	
Quintile 4	0.023	0.791	-0.009	0.826	0.073	0.347
Quintile 3	0.078	0.428	-0.032	0.501	0.083	0.351
Quintile 2	0.046	0.689	-0.033	0.569	0.071	0.499
Quintile 1	0.168	0.253	0.002	0.976	0.231	0.086
TX10P						
Quintile 5	Reference		Reference		Reference	
Quintile 4	-0.034	0.671	0.033	0.387	-0.047	0.543
Quintile 3	-0.068	0.445	0.050	0.245	-0.071	0.402
Quintile 2	-0.023	0.841	0.000	0.998	-0.086	0.392
Quintile 1	-0.061	0.567	0.019	0.710	-0.016	0.867
TXN						
Quintile 5	Reference		Reference		Reference	
Quintile 4	-0.012	0.900	0.010	0.831	0.036	0.667
Quintile 3	-0.033	0.745	-0.003	0.958	-0.104	0.257
Quintile 2	-0.108	0.257	0.011	0.820	-0.154	0.106
Quintile 1	-0.084	0.414	0.003	0.956	-0.121	0.223
TNN						
Quintile 5	Reference		Reference		Reference	
Quintile 4	0.043	0.603	-0.008	0.844	0.054	0.475

Quintile 3	0.085	0.339	0.022	0.636	0.091	0.312
Quintile 2	0.049	0.629	-0.031	0.568	0.091	0.351
Quintile 1	0.057	0.599	-0.029	0.615	0.143	0.175
**Other temperature**						
GSL						
Quintile 5	Reference		Reference		Reference	
Quintile 4	-0.018	0.857	-0.035	0.492	0.007	0.940
Quintile 3	-0.132	0.226	-0.061	0.267	-0.042	0.689
Quintile 2	-0.129	0.212	-0.056	0.308	-0.015	0.889
Quintile 1	-0.177	0.125	-0.046	0.415	-0.015	0.888
DTR						
Quintile 5	Reference		Reference		Reference	
Quintile 4	-0.053	0.525	-0.026	0.522	0.024	0.750
Quintile 3	0.036	0.707	0.010	0.851	0.079	0.384
Quintile 2	0.035	0.771	0.022	0.726	0.008	0.940
Quintile 1	0.009	0.952	0.025	0.736	0.045	0.758
**Extreme precipitation**						
CDD						
Quintile 5	Reference		Reference		Reference	
Quintile 4	0.070	0.378	0.050	0.261	0.089	0.248
Quintile 3	0.015	0.874	0.021	0.664	-0.034	0.696
Quintile 2	0.027	0.770	0.060	0.227	0.045	0.616
Quintile 1	-0.001	0.991	0.030	0.586	0.133	0.190
CWD						
Quintile 5	Reference		Reference		Reference	
Quintile 4	-0.056	0.559	-0.024	0.581	-0.093	0.267
Quintile 3	0.007	0.932	-0.027	0.505	-0.051	0.520
Quintile 2	0.052	0.598	-0.007	0.875	-0.166	0.058
Quintile 1	-0.061	0.581	-0.056	0.242	-0.089	0.335
PRCPTOT						
Quintile 5	Reference		Reference		Reference	
Quintile 4	0.033	0.722	-0.028	0.556	0.004	0.965
Quintile 3	0.063	0.601	-0.058	0.362	-0.170	0.202
Quintile 2	-0.088	0.535	-0.003	0.970	-0.080	0.569
Quintile 1	-0.059	0.732	-0.104	0.262	-0.155	0.387
R10						
Quintile 5	Reference		Reference		Reference	
Quintile 4	0.082	0.331	-0.019	0.674	0.054	0.546
Quintile 3	0.013	0.899	0.020	0.699	0.109	0.294
Quintile 2	0.051	0.055	0.040	0.487	0.181	0.114
Quintile 1	0.059	0.006	0.066	0.254	0.241	0.034
R20						

Quintile 5	Reference		Reference		Reference	
Quintile 4	0.042	0.611	0.000	0.992	-0.033	0.693
Quintile 3	-0.048	0.635	0.010	0.841	-0.061	0.541
Quintile 2	-0.002	0.989	0.018	0.751	-0.149	0.199
Quintile 1	0.007	0.957	0.078	0.214	-0.056	0.638
R95P						
Quintile 5	Reference		Reference		Reference	
Quintile 4	0.028	0.754	0.054	0.226	0.069	0.426
Quintile 3	0.000	0.997	0.082	0.117	-0.072	0.490
Quintile 2	0.023	0.843	0.068	0.253	-0.116	0.316
Quintile 1	-0.028	0.848	0.133	0.066	-0.070	0.603
R99P						
Quintile 5	Reference		Reference		Reference	
Quintile 4	-0.108	0.297	-0.051	0.312	-0.039	0.661
Quintile 3	-0.037	0.725	-0.023	0.676	-0.074	0.458
Quintile 2	0.061	0.595	-0.009	0.881	-0.119	0.243
Quintile 1	-0.011	0.932	-0.011	0.858	-0.085	0.431
SDII						
Quintile 5	Reference		Reference		Reference	
Quintile 4	0.029	0.759	0.034	0.462	0.119	0.204
Quintile 3	0.060	0.587	0.040	0.472	0.207	0.066
Quintile 2	0.069	0.606	-0.003	0.964	0.164	0.215
Quintile 1	0.087	0.576	0.023	0.765	0.289	0.048
RX1DAY						
Quintile 5	Reference		Reference		Reference	
Quintile 4	-0.050	0.546	-0.031	0.454	0.021	0.781
Quintile 3	-0.099	0.296	-0.049	0.298	-0.018	0.846
Quintile 2	-0.157	0.123	-0.043	0.406	0.108	0.256
Quintile 1	-0.051	0.672	-0.068	0.232	-0.032	0.770
RX5DAY						
Quintile 5	Reference		Reference		Reference	
Quintile 4	0.024	0.781	-0.091	0.140	-0.098	0.248
Quintile 3	0.014	0.865	-0.023	0.598	-0.014	0.861
Quintile 2	0.073	0.440	-0.054	0.257	0.043	0.637
Quintile 1	0.014	0.899	-0.056	0.318	-0.018	0.865

[a]Adjusted covariates of linear mixed effect model included MMSE score at baseline, age, gender, area of residence, educational attainment, marital status, number of chronic diseases, annual per capita household consumption level, and other environmental indices.

Supplementary table 23. Associations with climate extremes and cognitive decline in a population stratified by healthy lifestyle using Rubin's method: subgroup analyses by age

Characteristics	＜60						≥60					
	Unfavorable group		Average group		Favorable group		Unfavorable group		Average group		Favorable group	
	Estimate[a]	P value	Estimate[a]	P value	Estimate[a]	P value	Estimate[a]	P value	Estimate[a]	P value	Estimate[a]	P value
**Extreme heat**												
SU	-0.453	0.200	-0.227	0.179	-0.286	0.336	-0.282	0.468	-0.296	0.171	0.094	0.807
TR	0.091	0.819	0.291	0.168	0.306	0.435	0.469	0.302	-0.066	0.814	-0.016	0.976
WSDI	0.009	0.719	-0.002	0.885	-0.023	0.361	-0.039	0.221	0.001	0.935	-0.074	0.014
TN90P	0.030	0.171	0.008	0.504	0.008	0.653	0.015	0.546	0.005	0.756	0.003	0.892
TX90P	-0.010	0.692	0.010	0.412	0.005	0.832	0.000	0.992	0.014	0.398	0.006	0.835
TXX	-0.119	0.019	-0.106	0.060	-0.092	0.383	-0.232	0.023	-0.102	0.203	-0.106	0.422
TNX	0.020	0.906	-0.138	0.106	-0.174	0.265	-0.227	0.272	-0.042	0.715	0.114	0.505
**Extreme cold**												
FD	-0.633	0.248	0.128	0.633	-0.201	0.680	-0.131	0.838	-0.272	0.443	-0.204	0.731
CSDI	-0.002	0.928	-0.004	0.767	0.001	0.949	0.024	0.377	-0.002	0.884	-0.028	0.294
ID	-0.261	0.458	-0.216	0.228	-0.241	0.487	-0.054	0.896	-0.135	0.569	0.244	0.560
TN10P	-0.021	0.458	0.016	0.294	-0.008	0.785	-0.035	0.291	0.006	0.754	-0.020	0.546
TX10P	0.010	0.554	-0.014	0.082	-0.007	0.630	0.000	0.985	-0.010	0.329	0.023	0.234
TXN	-0.261	0.037	-0.321	0.337	-0.174	0.518	-0.204	0.582	-0.210	0.300	-0.057	0.873
TNN	-0.021	0.953	0.337	0.087	0.254	0.470	0.488	0.267	0.028	0.913	0.483	0.260
**Other temperature**												
GSL	0.106	0.618	0.077	0.465	0.085	0.666	0.085	0.736	0.091	0.520	0.011	0.960
DTR	0.470	0.264	-0.020	0.922	0.239	0.534	0.915	0.146	-0.072	0.797	0.415	0.378

**Extreme precipitation**												
CDD	0.010	0.852	0.018	0.536	0.028	0.588	0.064	0.374	-0.014	0.732	0.029	0.706
CWD	0.031	0.502	-0.018	0.451	0.031	0.466	-0.010	0.869	0.006	0.845	-0.003	0.954
PRCPTOT	0.425	0.030	-0.012	0.973	-0.058	0.869	0.191	0.035	0.148	0.539	-0.393	0.336
R10	-0.197	0.044	-0.249	0.159	-0.180	0.308	-0.133	0.548	-0.101	0.401	0.015	0.946
R20	-0.007	0.964	-0.113	0.161	0.111	0.437	0.081	0.656	-0.089	0.366	0.015	0.929
R95P	-0.019	0.861	-0.054	0.338	0.025	0.808	-0.072	0.587	-0.027	0.700	0.065	0.566
R99P	-0.011	0.821	-0.020	0.404	-0.008	0.857	-0.006	0.924	0.008	0.788	0.019	0.713
SDII	0.016	0.923	-0.048	0.567	0.030	0.842	-0.030	0.878	-0.013	0.900	0.031	0.869
RX1DAY	0.023	0.731	-0.011	0.756	0.025	0.687	0.023	0.753	-0.007	0.868	0.083	0.280
RX5DAY	0.030	0.667	0.024	0.489	0.037	0.541	-0.014	0.858	0.056	0.209	-0.131	0.095

[a]Adjusted covariates of linear mixed effect model included MMSE score at baseline, age, gender, area of residence, educational attainment, marital status, number of chronic diseases, annual per capita household consumption level, and other environmental indices.

Supplementary table 24. Associations with climate extremes and cognitive decline in a population stratified by healthy lifestyle using Rubin's method: subgroup analyses by gender

Characteristics	Male						Female					
	Unfavorable group		Average group		Favorable group		Unfavorable group		Average group		Favorable group	
	Estimate[a]	P value	Estimate[a]	P value	Estimate[a]	P value	Estimate[a]	P value	Estimate[a]	P value	Estimate[a]	P value
**Extreme heat**												
SU	-0.415	0.020	-0.339	0.089	-0.452	0.619	-0.162	0.032	-0.207	0.234	-0.187	0.446
TR	0.159	0.688	0.222	0.365	0.870	0.562	0.250	0.626	0.170	0.458	0.297	0.362
WSDI	0.001	0.974	-0.015	0.368	0.010	0.926	-0.041	0.265	0.020	0.160	-0.039	0.147
TN90P	0.022	0.299	0.014	0.311	0.090	0.304	0.012	0.708	0.002	0.892	0.001	0.933
TX90P	-0.004	0.883	0.019	0.216	-0.017	0.864	0.001	0.981	0.003	0.832	0.007	0.731
TXX	-0.060	0.029	-0.171	0.015	0.306	0.417	-0.034	0.818	-0.066	0.260	-0.065	0.442
TNX	-0.030	0.856	-0.068	0.500	-1.119	0.053	-0.132	0.579	-0.167	0.066	-0.002	0.986
**Extreme cold**												
FD	-0.494	0.340	-0.286	0.379	1.320	0.405	-0.297	0.694	0.270	0.335	-0.224	0.566
CSDI	0.003	0.907	-0.005	0.743	0.020	0.761	0.016	0.632	0.004	0.771	-0.013	0.461
ID	-0.159	0.634	-0.259	0.218	0.411	0.758	-0.097	0.844	-0.176	0.358	-0.107	0.700
TN10P	-0.026	0.338	0.024	0.209	0.025	0.778	-0.019	0.646	-0.003	0.842	-0.008	0.742
TX10P	0.005	0.729	-0.016	0.101	0.050	0.316	0.011	0.668	-0.009	0.295	0.001	0.951
TXN	-0.157	0.035	-0.304	0.093	-1.270	0.232	0.063	0.886	-0.233	0.146	-0.059	0.790
TNN	-0.190	0.585	0.396	0.086	0.290	0.791	0.486	0.312	0.092	0.655	0.420	0.135
**Other temperature**												
GSL	0.207	0.304	-0.037	0.774	0.630	0.352	-0.107	0.734	0.205	0.060	0.062	0.683
DTR	0.566	0.161	0.043	0.867	-0.647	0.573	0.306	0.583	-0.015	0.941	0.347	0.267

**Extreme precipitation**												
CDD	0.021	0.688	-0.004	0.906	0.276	0.181	0.040	0.587	0.023	0.441	0.039	0.383
CWD	-0.011	0.804	-0.015	0.619	0.116	0.641	0.085	0.238	0.000	0.984	0.009	0.787
PRCPTOT	0.323	0.039	0.293	0.198	1.431	0.222	0.414	0.037	0.436	0.329	-0.106	0.707
R10	-0.254	0.046	-0.166	0.155	-0.644	0.205	-0.176	0.020	-0.226	0.420	-0.132	0.362
R20	-0.068	0.666	-0.107	0.250	-0.303	0.565	0.031	0.878	-0.160	0.056	0.060	0.600
R95P	0.035	0.749	-0.101	0.134	0.040	0.906	-0.191	0.209	-0.042	0.454	0.041	0.607
R99P	-0.013	0.790	0.004	0.885	-0.334	0.043	0.011	0.874	-0.022	0.361	0.013	0.697
SDII	-0.043	0.787	0.006	0.952	-0.394	0.564	0.155	0.468	-0.061	0.474	-0.009	0.941
RX1DAY	-0.013	0.829	0.025	0.528	-0.116	0.611	0.087	0.320	-0.029	0.400	0.035	0.484
RX5DAY	0.027	0.689	0.019	0.655	0.491	0.023	0.001	0.989	0.046	0.196	-0.016	0.753

[a]Adjusted covariates of linear mixed effect model included MMSE score at baseline, age, area of residence, educational attainment, marital status, number of chronic diseases, annual per capita household consumption level, and other environmental indices.

Supplementary table 25. Associations with climate extremes and cognitive decline in a population stratified by healthy lifestyle using Rubin's method: subgroup analyses by area of residence

Characteristics	Urban community						Rural village					
	Unfavorable group		Average group		Favorable group		Unfavorable group		Average group		Favorable group	
	Estimate[a]	P value	Estimate[a]	P value	Estimate[a]	P value	Estimate[a]	P value	Estimate[a]	P value	Estimate[a]	P value
**Extreme heat**												
SU	-0.447	0.126	-0.223	0.167	-0.148	0.735	-0.161	0.015	-0.288	0.259	-0.166	0.549
TR	0.391	0.044	0.306	0.345	0.242	0.652	-0.218	0.700	-0.083	0.805	0.065	0.865
WSDI	0.015	0.535	0.002	0.878	-0.061	0.072	-0.028	0.382	-0.009	0.644	-0.018	0.427
TN90P	0.013	0.519	0.011	0.336	-0.001	0.984	0.009	0.781	-0.004	0.806	0.002	0.910
TX90P	-0.011	0.647	-0.005	0.723	0.013	0.716	-0.006	0.860	0.034	0.072	0.009	0.687
TXX	-0.018	0.855	-0.101	0.097	-0.142	0.391	-0.019	0.901	-0.113	0.176	-0.104	0.269
TNX	0.050	0.745	-0.159	0.072	0.097	0.681	-0.167	0.474	-0.058	0.633	-0.143	0.315
**Extreme cold**												
FD	-0.491	0.283	-0.038	0.876	-0.413	0.549	-0.410	0.651	-0.190	0.676	0.438	0.364
CSDI	0.004	0.818	-0.007	0.501	-0.019	0.518	-0.020	0.568	0.003	0.874	0.003	0.871
ID	-0.364	0.044	-0.157	0.613	-0.414	0.408	-0.023	0.962	-0.069	0.790	0.104	0.738
TN10P	-0.008	0.761	0.008	0.608	0.016	0.711	-0.034	0.398	0.023	0.312	-0.045	0.074
TX10P	0.002	0.882	-0.009	0.278	0.009	0.676	-0.003	0.910	-0.010	0.401	0.007	0.641
TXN	-0.385	0.013	-0.161	0.530	-0.004	0.992	-0.124	0.755	-0.091	0.682	0.038	0.873
TNN	0.014	0.961	0.220	0.200	0.269	0.562	0.695	0.227	0.371	0.254	0.315	0.365
**Other temperature**												
GSL	0.078	0.666	0.030	0.759	-0.005	0.985	0.116	0.714	0.120	0.477	0.225	0.194
DTR	0.404	0.330	0.231	0.330	0.300	0.647	0.594	0.270	-0.281	0.363	0.212	0.545

**Extreme precipitation**												
CDD	0.000	1.000	0.034	0.263	0.075	0.352	0.106	0.157	-0.016	0.720	0.003	0.958
CWD	0.015	0.734	-0.005	0.844	-0.008	0.906	-0.036	0.588	-0.025	0.478	0.028	0.453
PRCPTOT	-0.186	0.607	0.337	0.073	-0.250	0.613	0.784	0.105	0.221	0.429	0.175	0.570
R10	-0.160	0.047	-0.109	0.255	-0.065	0.802	-0.313	0.045	-0.211	0.127	-0.335	0.166
R20	0.089	0.480	-0.122	0.071	0.326	0.098	-0.231	0.349	-0.072	0.591	-0.073	0.580
R95P	0.012	0.905	-0.058	0.263	0.051	0.721	-0.016	0.919	-0.078	0.357	0.010	0.912
R99P	-0.029	0.518	-0.010	0.698	-0.009	0.886	0.059	0.393	-0.007	0.831	0.009	0.799
SDII	0.051	0.711	0.026	0.734	-0.160	0.481	-0.239	0.336	-0.046	0.732	-0.012	0.930
RX1DAY	0.004	0.939	-0.027	0.409	0.043	0.652	0.014	0.873	0.036	0.459	0.061	0.249
RX5DAY	0.026	0.694	0.036	0.289	0.036	0.700	-0.027	0.769	0.010	0.849	-0.023	0.690

[a]Adjusted covariates of linear mixed effect model included MMSE score at baseline, age, gender, educational attainment, marital status, number of chronic diseases, annual per capita household consumption level, and other environmental indices.

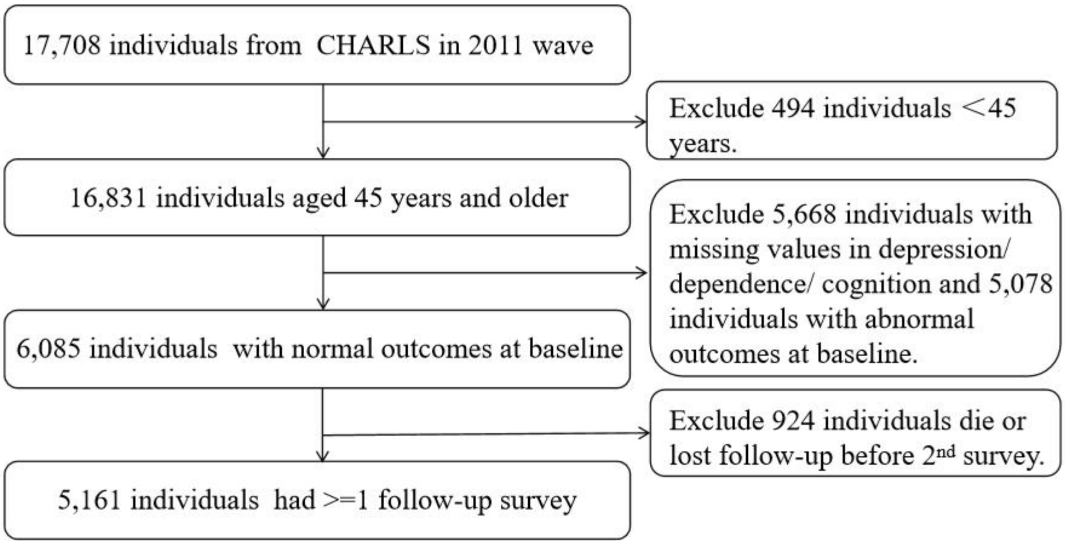

Supplementary figure 1. Flow chart of the included CHARLS individuals.

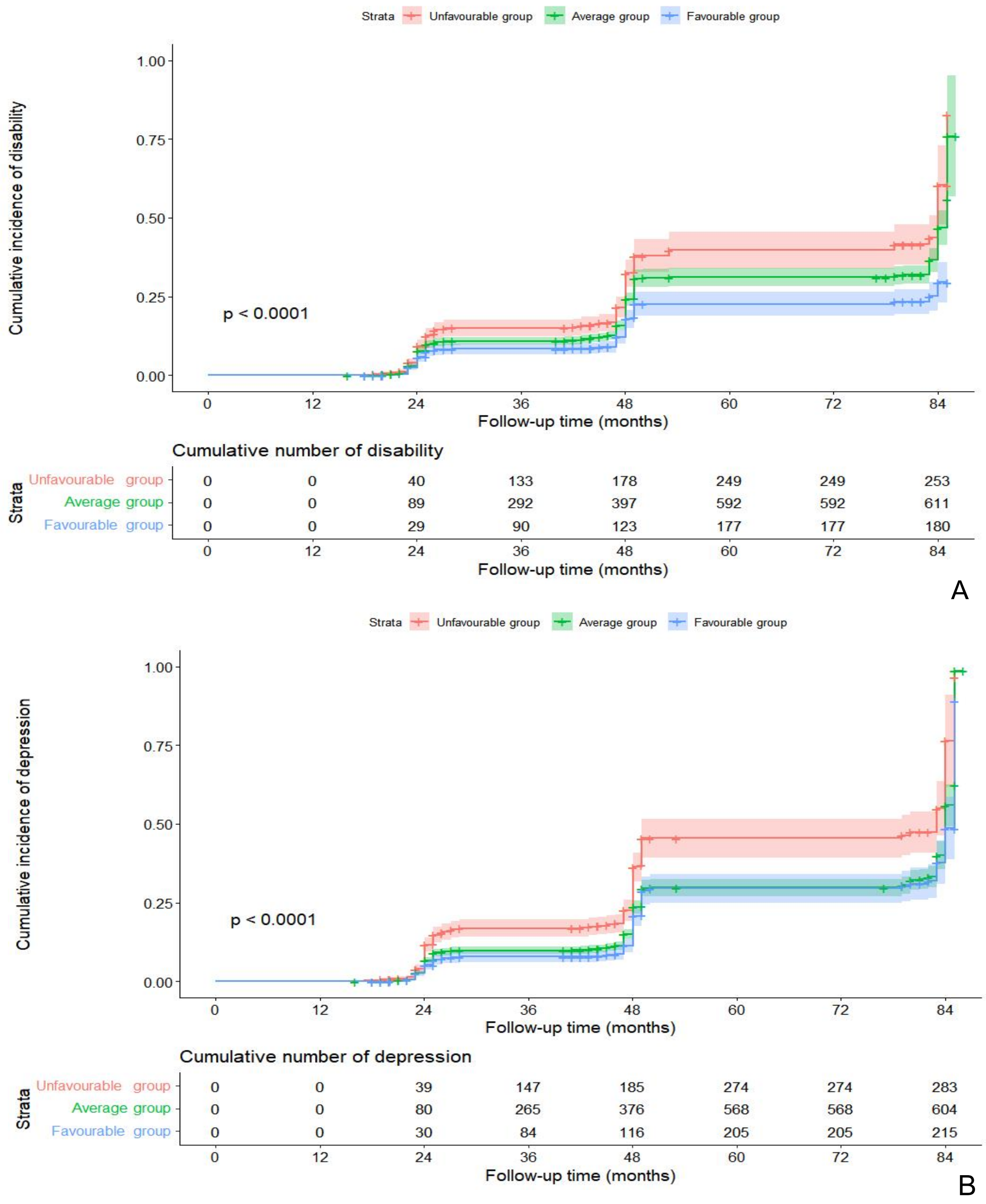

Supplementary figure 2. Kaplan-Meier curves for the overall cumulative incidence of outcome and number at risk. The shaded area indicates the range of 95% CIs for the corresponding cumulative incidence curve. P-value indicates the significance level from the comparison of incidence curves using the Log-rank test. A: disability; B: depression.

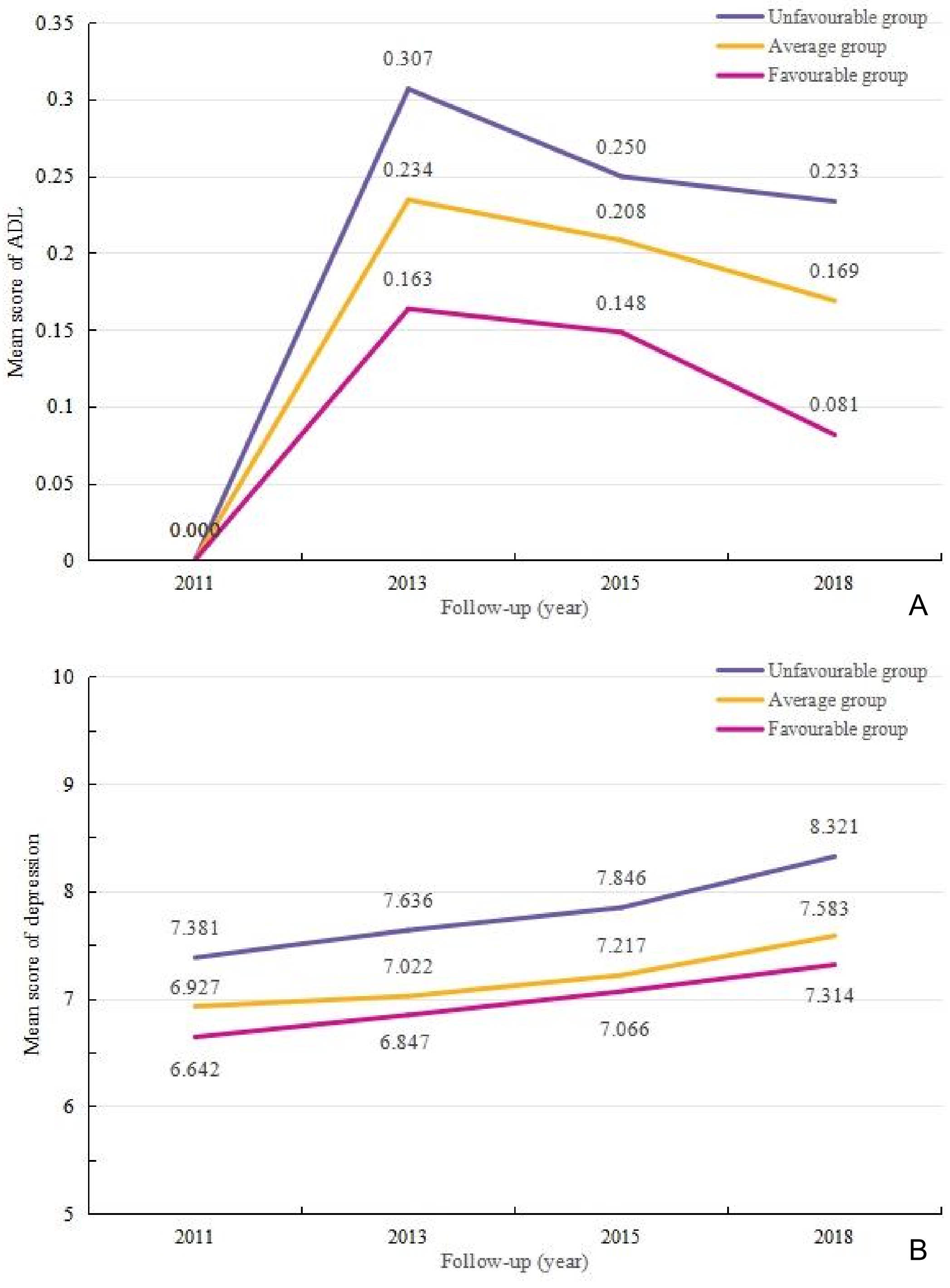

Supplementary figure 3. Longitudinal change in mean scores among different lifestyle groups over 7 years. A: mean score of ADL scale; B: mean score of depression scale.

STROBE Statement—Checklist of items that should be included in reports of ***cohort studies***

	Item No	Recommendation	Page No
**Title and abstract**	1	(*a*) Indicate the study's design with a commonly used term in the title or the abstract	1
		(*b*) Provide in the abstract an informative and balanced summary of what was done and what was found	1-2
**Introduction**			
Background/rationale	2	Explain the scientific background and rationale for the investigation being reported	2-3
Objectives	3	State specific objectives, including any prespecified hypotheses	3
**Methods**			
Study design	4	Present key elements of study design early in the paper	3-4
Setting	5	Describe the setting, locations, and relevant dates, including periods of recruitment, exposure, follow-up, and data collection	3-4
Participants	6	(*a*) Give the eligibility criteria, and the sources and methods of selection of participants. Describe methods of follow-up	3-4
		(*b*) For matched studies, give matching criteria and number of exposed and unexposed	/
Variables	7	Clearly define all outcomes, exposures, predictors, potential confounders, and effect modifiers. Give diagnostic criteria, if applicable	4-7
Data sources/ measurement	8*	For each variable of interest, give sources of data and details of methods of assessment (measurement). Describe comparability of assessment methods if there is more than one group	4-7
Bias	9	Describe any efforts to address potential sources of bias	4-7
Study size	10	Explain how the study size was arrived at	7
Quantitative variables	11	Explain how quantitative variables were handled in the analyses. If applicable, describe which groupings were chosen and why	7
Statistical methods	12	(*a*) Describe all statistical methods, including those used to control for confounding	7-8
		(*b*) Describe any methods used to examine subgroups and interactions	7-8
		(*c*) Explain how missing data were addressed	7
		(*d*) If applicable, explain how loss to follow-up was addressed	8
		(*e*) Describe any sensitivity analyses	8
**Results**			
Participants	13*	(a) Report numbers of individuals at each stage of study—eg numbers potentially eligible, examined for eligibility, confirmed eligible, included in the study, completing follow-up, and analysed	4
		(b) Give reasons for non-participation at each stage	4
		(c) Consider use of a flow diagram	4
Descriptive data	14*	(a) Give characteristics of study participants (eg demographic, clinical, social) and information on exposures and potential confounders	9

		(b) Indicate number of participants with missing data for each variable of interest	7
		(c) Summarise follow-up time (eg, average and total amount)	9
Outcome data	15*	Report numbers of outcome events or summary measures over time	9
Main results	16	(*a*) Give unadjusted estimates and, if applicable, confounder-adjusted estimates and their precision (eg, 95% confidence interval). Make clear which confounders were adjusted for and why they were included	9-11
		(*b*) Report category boundaries when continuous variables were categorized	4-6
		(*c*) If relevant, consider translating estimates of relative risk into absolute risk for a meaningful time period	/
Other analyses	17	Report other analyses done—eg analyses of subgroups and interactions, and sensitivity analyses	10-11
**Discussion**			
Key results	18	Summarise key results with reference to study objectives	12
Limitations	19	Discuss limitations of the study, taking into account sources of potential bias or imprecision. Discuss both direction and magnitude of any potential bias	15
Interpretation	20	Give a cautious overall interpretation of results considering objectives, limitations, multiplicity of analyses, results from similar studies, and other relevant evidence	12-15
Generalisability	21	Discuss the generalisability (external validity) of the study results	12-14
**Other information**			
Funding	22	Give the source of funding and the role of the funders for the present study and, if applicable, for the original study on which the present article is based	16

*Give information separately for exposed and unexposed groups.

Note: An Explanation and Elaboration article discusses each checklist item and gives methodological background and published examples of transparent reporting. The STROBE checklist is best used in conjunction with this article (freely available on the Web sites of PLoS Medicine at http://www.plosmedicine.org/, Annals of Internal Medicine at http://www.annals.org/, and Epidemiology at http://www.epidem.com/). Information on the STROBE Initiative is available at http://www.strobe-statement.org.
